# Supplementary material for: Facilitators of and barriers to healthcare providers’ adoption of harm reduction in cannabis use: a scoping review protocol
Source: BMJ Open. 2024 Apr 5;14(4):e078427. doi: 10.1136/bmjopen-2023-078427 (PMC11002399; doi:10.1136/bmjopen-2023-078427)
Supplement: Supplementary data [file bmjopen-2023-078427supp001.pdf]

Facilitators of and barriers to healthcare providers' adoption of harm reduction in cannabis use: a scoping review protocol

Roula Haddad, Christian Dagenais, Christophe Huynh, Jean-Sébastien Fallu

Supplemental Appendix: Search strategies executed on all databases

Appendix 1: Medline Search Strategy

#	Concept	Equations	Results
1	Harm reduction	Harm Reduction/	3898
2		Risk Reduction Behavior/	14132
3		(protective adj2 strateg*).ab,kf,ti.	2818
4		((reduc* or minimi*) adj5 (harm? or harmful or risk?)).ab,kf,ti.	239459
5		1 or 2 or 3 or 4	251821
6	Clinicians	exp Health Personnel/	588634
7		Social Workers/	971
8		Counselors/	541
9		exp Health Occupations/	1819039
10		exp Allied Health Occupations/	52626
11		exp Allied Health Personnel/	53169
12		(worker? or psychoeducator? or psycho-educator? or educator? or nurse? or criminologist? or psychologist? or clinician? or practitioner? or physician? or professional? or provider? or co?nselor or co?nselors or caregiver? or giver? or therapist? or psychotherapist? or staff? or personnel? or employee? or doctor?).ab,kf,ti.	2002544
13		6 or 7 or 8 or 9 or 10 or 11 or 12	3625104
14	Cannabis	Cannabis/	12307
15		"Marijuana Use"/	1689
16		Marijuana Abuse/	6905
17		Marijuana Smoking/	5425
18		(mari?uana or cannabis or hashish or Pot or weed or tetrahydrocannabinol or THC or CDB or cannabidiol or cannabinoid?).ab,kf,ti.	100005
19		14 or 15 or 16 or 17 or 18	102988
20	Strategies	("strateg*" or "approach*" or intervention? or prevent* or practice? or service? or "method*" or technique? or tactic? or co?nseling or treatment? or program? or "guide*").ab,kf,ti.	15074452
21	Combination of 3 concepts	5 and 13 and 19	272
22	Limit date	limit 21 to yr="1990 -Current"	272
23	Limit language	limit 22 to (english or french)	263
24	Filter OECD		
25	TOTAL	23 not 24	249
22	Combination of 4 concepts	5 and 13 and 19 and 20	251

Appendix 2: PsycINFO

(((((((title: (reduc*))) OR ((title: (minimi*)))) NEAR/5 (((title: (harm))) OR ((title: (harms))) OR ((title: (harmful))) OR ((title: (risk))) OR ((title: (risks)))) OR (((abstract: (reduc*))) OR ((abstract: (minimi*)))) NEAR/5 (((abstract: (harm))) OR ((abstract: (harms))) OR ((abstract: (harmful))) OR ((abstract: (risk))) OR ((abstract: (risks)))) OR (((Keywords: (reduc*))) OR ((Keywords: (minimi*)))) NEAR/5 (((Keywords: (harm))) OR ((Keywords: (harms))) OR ((Keywords: (harmful))) OR ((Keywords: (risk))) OR ((Keywords: (risks))))) OR ((((title: (protective NEAR/2 strateg*)))) OR (((abstract: (protective NEAR/2 strateg*)))) OR (((Keywords: (protective NEAR/2 strateg*))))) OR ((((MeSH: (Risk Reduction Behavior))))) OR ((((MeSH: (Harm Reduction)))))) AND (((((MeSH: (Health Personnel))))) OR ((((MeSH: (Social Workers))))) OR ((((MeSH: (Counselors))))) OR ((((MeSH: (Health Occupations))))) OR ((((MeSH: (Allied Health Occupations))))) OR ((((MeSH: (Allied Health Personnel))))) OR ((((Keywords: (worker*))) OR ((Keywords: (psychoeducator*))) OR ((Keywords: (psycho-educator*))) OR ((Keywords: (educator*))) OR ((Keywords: (nurse*))) OR ((Keywords: (criminologist*))) OR ((Keywords: (psychologist*))) OR ((Keywords: (clinician*))) OR ((Keywords: (practitioner*))) OR ((Keywords: (physician*))) OR ((Keywords: (professional*))) OR ((Keywords: (provider*))) OR ((Keywords: (conselor))) OR ((Keywords: (conselors))) OR ((Keywords: (counselor))) OR ((Keywords: (counselers))) OR ((Keywords: (caregiver*))) OR ((Keywords: (giver*))) OR ((Keywords: (therapist*))) OR ((Keywords: (psychotherapist*))) OR ((Keywords: (staff*))) OR ((Keywords: (personnel*))) OR ((Keywords: (employee*))) OR ((Keywords: (doctor*)))) OR (((abstract: (worker*))) OR ((abstract: (psychoeducator*))) OR ((abstract: (psycho-educator*))) OR ((abstract: (educator*))) OR ((abstract: (nurse*))) OR ((abstract: (criminologist*))) OR ((abstract: (psychologist*))) OR ((abstract: (clinician*))) OR ((abstract: (practitioner*))) OR ((abstract: (physician*))) OR ((abstract: (professional*))) OR ((abstract: (provider*))) OR ((abstract: (conselor))) OR ((abstract: (conselors))) OR ((abstract: (counselor))) OR ((abstract: (counselers))) OR ((abstract: (caregiver*))) OR ((abstract: (giver*))) OR ((abstract: (therapist*))) OR ((abstract: (psychotherapist*))) OR ((abstract: (staff*))) OR ((abstract: (personnel*))) OR ((abstract: (employee*))) OR ((abstract: (doctor*)))))) AND (((((Keywords: (marijuana))) OR ((Keywords: (marihuana))) OR ((Keywords: (cannabis))) OR ((Keywords: (hashish))) OR ((Keywords: (Pot))) OR ((Keywords: (weed))) OR ((Keywords: (tetrahydrocannabinol))) OR ((Keywords: (THC))) OR ((Keywords: (CDB))) OR ((Keywords: (cannabidiol))) OR ((Keywords: (cannabinoid))) OR ((Keywords: (cannabinoids)))) OR (((abstract: (marijuana))) OR ((abstract: (marihuana))) OR ((abstract: (cannabis))) OR ((abstract: (hashish))) OR ((abstract: (Pot))) OR ((abstract: (weed))) OR ((abstract: (tetrahydrocannabinol))) OR ((abstract: (THC))) OR ((abstract: (CDB))) OR ((abstract: (cannabidiol))) OR ((abstract: (cannabinoid))) OR ((abstract: (cannabinoids))))) OR ((((MeSH: (Marijuana Smoking))))) OR ((((MeSH: (Marijuana Abuse))))) OR ((((MeSH: ("Marijuana Use"))))) OR ((((MeSH: (Cannabis))))))) AND ((Year: [1990 TO 9999]))) NOT ((((Keywords: (afghanistan/)) OR (Keywords: (africa/)) OR (Keywords: (africa, northern/)) OR (Keywords: (africa, central/)) OR (Keywords: (africa, eastern/)) OR (Keywords: ("africa south of the sahara" /)) OR (Keywords: (africa, southern/)) OR (Keywords: (africa, western/)) OR (Keywords: (albania/)) OR (Keywords: (algeria/)) OR (Keywords: (andorra/)) OR (Keywords: (angola/)) OR (Keywords: ("antigua and barbuda" /)) OR (Keywords: (argentina/)) OR (Keywords: (armenia/)) OR (Keywords: (azerbaijan/)) OR (Keywords: (bahamas/)) OR (Keywords: (bahrain/)) OR (Keywords: (bangladesh/)) OR (Keywords: (barbados/)) OR (Keywords: (belize/)) OR (Keywords: (benin/)) OR (Keywords: (bhutan/)) OR (Keywords: (bolivia/)) OR (Keywords: (borneo/)) OR (Keywords: ("bosnia and herzegovina" /)) OR

(Keywords: (botswana/)) OR (Keywords: (brazil/)) OR (Keywords: (brunei/)) OR (Keywords: (bulgaria/)) OR (Keywords: (burkina faso/)) OR (Keywords: (burundi/)) OR (Keywords: (cabo verde/)) OR (Keywords: (cambodia/)) OR (Keywords: (cameroon/)) OR (Keywords: (central african republic/)) OR (Keywords: (chad/)) OR (Keywords: (exp china/)) OR (Keywords: (comoros/)) OR (Keywords: (congo/)) OR (Keywords: (cote d'ivoire/)) OR (Keywords: (croatia/)) OR (Keywords: (cuba/)) OR (Keywords: ("democratic republic of the congo" /)) OR (Keywords: (cyprus/)) OR (Keywords: (djibouti/)) OR (Keywords: (dominica/)) OR (Keywords: (dominican republic/)) OR (Keywords: (ecuador/)) OR (Keywords: (egypt/)) OR (Keywords: (el salvador/)) OR (Keywords: (equatorial guinea/)) OR (Keywords: (eritrea/)) OR (Keywords: (eswatini/)) OR (Keywords: (ethiopia/)) OR (Keywords: (fiji/)) OR (Keywords: (gabon/)) OR (Keywords: (gambia/)) OR (Keywords: ("georgia (republic)" /)) OR (Keywords: (ghana/)) OR (Keywords: (grenada/)) OR (Keywords: (guatemala/)) OR (Keywords: (guinea/)) OR (Keywords: (guinea-bissau/)) OR (Keywords: (guyana/)) OR (Keywords: (haiti/)) OR (Keywords: (honduras/)) OR (Keywords: (independent state of samoa/)) OR (Keywords: (exp india/)) OR (Keywords: (indian ocean islands/)) OR (Keywords: (indochina/)) OR (Keywords: (indonesia/)) OR (Keywords: (iran/)) OR (Keywords: (iraq/)) OR (Keywords: (jamaica/)) OR (Keywords: (jordan/)) OR (Keywords: (kazakhstan/)) OR (Keywords: (kenya/)) OR (Keywords: (kosovo/)) OR (Keywords: (kuwait/)) OR (Keywords: (kyrgyzstan/)) OR (Keywords: (laos/)) OR (Keywords: (lebanon/)) OR (Keywords: (liechtenstein/)) OR (Keywords: (lesotho/)) OR (Keywords: (liberia/)) OR (Keywords: (libya/)) OR (Keywords: (madagascar/)) OR (Keywords: (malaysia/)) OR (Keywords: (malawi/)) OR (Keywords: (mali/)) OR (Keywords: (malta/)) OR (Keywords: (mauritania/)) OR (Keywords: (mauritius/)) OR (Keywords: (mekong valley/)) OR (Keywords: (melanesia/)) OR (Keywords: (micronesia/)) OR (Keywords: (monaco/)) OR (Keywords: (mongolia/)) OR (Keywords: (montenegro/)) OR (Keywords: (morocco/)) OR (Keywords: (mozambique/)) OR (Keywords: (myanmar/)) OR (Keywords: (namibia/)) OR (Keywords: (nepal/)) OR (Keywords: (nicaragua/)) OR (Keywords: (niger/)) OR (Keywords: (nigeria/)) OR (Keywords: (oman/)) OR (Keywords: (pakistan/)) OR (Keywords: (palau/)) OR (Keywords: (exp panama/)) OR (Keywords: (papua new guinea/)) OR (Keywords: (paraguay/)) OR (Keywords: (peru/)) OR (Keywords: (philippines/)) OR (Keywords: (qatar/)) OR (Keywords: ("republic of belarus" /)) OR (Keywords: ("republic of north macedonia" /)) OR (Keywords: (romania/)) OR (Keywords: (exp russia/)) OR (Keywords: (rwanda/)) OR (Keywords: ("saint kitts and nevis" /)) OR (Keywords: (saint lucia/)) OR (Keywords: ("saint vincent and the grenadines" /)) OR (Keywords: ("sao tome and principe" /)) OR (Keywords: (saudi arabia/)) OR (Keywords: (serbia/)) OR (Keywords: (sierra leone/)) OR (Keywords: (senegal/)) OR (Keywords: (seychelles/)) OR (Keywords: (singapore/)) OR (Keywords: (somalia/)) OR (Keywords: (south africa/)) OR (Keywords: (south sudan/)) OR (Keywords: (sri lanka/)) OR (Keywords: (sudan/)) OR (Keywords: (suriname/)) OR (Keywords: (syria/)) OR (Keywords: (taiwan/)) OR (Keywords: (tajikistan/)) OR (Keywords: (tanzania/)) OR (Keywords: (thailand/)) OR (Keywords: (timor-leste/)) OR (Keywords: (togo/)) OR (Keywords: (tonga/)) OR (Keywords: ("trinidad and tobago" /)) OR (Keywords: (tunisia/)) OR (Keywords: (turkmenistan/)) OR (Keywords: (uganda/)) OR (Keywords: (ukraine/)) OR (Keywords: (united arab emirates/)) OR (Keywords: (uruguay/)) OR (Keywords: (uzbekistan/)) OR (Keywords: (vanuatu/)) OR (Keywords: (venezuela/)) OR (Keywords: (vietnam/)) OR (Keywords: (west indies/)) OR (Keywords: (yemen/)) OR (Keywords: (zambia/)) OR (Keywords: (zimbabwe/)))) AND Any Field: - (((Keywords: (australasia/)) OR (Keywords: (exp australia/)) OR (Keywords: (austria/)) OR (Keywords: (baltic states/)) OR (Keywords: (belgium/)) OR (Keywords: (exp canada/)) OR (Keywords: (chile/)) OR (Keywords: (colombia/)) OR (Keywords: (costa rica/)) OR

(Keywords: (czech republic/)) OR (Keywords: (exp denmark/)) OR (Keywords: (estonia/)) OR (Keywords: (europe/)) OR (Keywords: (finland/)) OR (Keywords: (exp france/)) OR (Keywords: (exp germany/)) OR (Keywords: (greece/)) OR (Keywords: (hungary/)) OR (Keywords: (iceland/)) OR (Keywords: (ireland/)) OR (Keywords: (israel/)) OR (Keywords: (exp italy/)) OR (Keywords: (exp japan/)) OR (Keywords: (korea/)) OR (Keywords: (latvia/)) OR (Keywords: (lithuania/)) OR (Keywords: (luxembourg/)) OR (Keywords: (mexico/)) OR (Keywords: (netherlands/)) OR (Keywords: (new zealand/)) OR (Keywords: (north america/)) OR (Keywords: (exp norway/)) OR (Keywords: (poland/)) OR (Keywords: (portugal/)) OR (Keywords: (exp "republic of korea" /)) OR (Keywords: ("scandinavian and nordic countries" /)) OR (Keywords: (slovakia/)) OR (Keywords: (slovenia/)) OR (Keywords: (spain/)) OR (Keywords: (sweden/)) OR (Keywords: (switzerland/)) OR (Keywords: (turkey/)) OR (Keywords: (exp united kingdom/)) OR (Keywords: (exp united states/)) OR (Keywords: (European Union/)) OR (Keywords: (Developed Countries/)))))

Appendix 3: CINAHL

#	Question	Results
S1	MW "harm# reduction#"	4,889
S2	MW "risk# reduction#"	0
S3	TI protective N2 strateg* OR AB protective N2 strateg* OR SU protective N2 strateg*	1,167
S4	SU ((reduc* or minimi*) N5 (harm# or harmful or risk#)) OR TI ((reduc* or minimi*) N5 (harm# or harmful or risk#)) OR AB ((reduc* or minimi*) N5 (harm# or harmful or risk#))	96,037
S5	S1 OR S2 OR S3 OR S4	97,005
S6	MW "Health Personnel#"	112,080
S7	MW "Social Worker#"	11,786
S8	MW Counselors	4,471
S9	MW "Health Occupation#"	5,719
S10	MW "Allied Health Occupation#"	0
S11	MW "Allied Health Personnel#"	4,781
S12	TI (worker or psychoeducator or psycho-educator or educator or nurse or criminologist or psychologist or clinician or practitioner or physician or professional or provider or counse#lor or counse#lors or caregiver or giver or therapist or psychotherapist or staff or personnel or employee or doctor) OR AB (worker or psychoeducator or psycho-educator or educator or nurse or criminologist or psychologist or clinician or practitioner or physician or professional or provider or counse#lor or counse#lors or caregiver or giver or therapist or psychotherapist or staff or personnel or employee or doctor) OR SU (worker or psychoeducator or psycho-educator or educator or nurse or criminologist or psychologist or clinician or practitioner or physician or professional or provider or counse#lor or counse#lors or caregiver or giver or therapist or psychotherapist or staff or personnel or employee or doctor)	1,762,879
S13	S6 OR S7 OR S8 OR S9 OR S10 OR S11 OR S12	1,765,867

S14	MW Cannabis	11,138
S15	MW Marijuana	2,219
S16	TI (mari#uana or cannabis or hashish or Pot or weed or tetrahydrocannabinol or THC or CDB or cannabidiol or cannabinoid#) OR AB (mari#uana or cannabis or hashish or Pot or weed or tetrahydrocannabinol or THC or CDB or cannabidiol or cannabinoid#) OR SU (mari#uana or cannabis or hashish or Pot or weed or tetrahydrocannabinol or THC or CDB or cannabidiol or cannabinoid#)	26,526
S17	S14 OR S15 OR S16	26,526
S18	S5 AND S13 AND S17	147
S19	S5 AND S13 AND S17	147
S20	S5 AND S13 AND S17	147
S21	S5 AND S13 AND S17	72
S22	SU afghanistan/ or africa/ or africa, northern/ or africa, central/ or africa, eastern/ or "africa south of the sahara"/ or africa, southern/ or africa, western/ or albania/ or algeria/ or andorra/ or angola/ or "antigua and barbuda"/ or argentina/ or armenia/ or azerbaijan/ or bahamas/ or bahrain/ or bangladesh/ or barbados/ or belize/ or benin/ or bhutan/ or bolivia/ or borneo/ or "bosnia and herzegovina"/ or botswana/ or brazil/ or brunei/ or bulgaria/ or burkina faso/ or burundi/ or cabo verde/ or cambodia/ or cameroon/ or central african republic/ or chad/ or exp china/ or comoros/ or congo/ or cote d'ivoire/ or croatia/ or cuba/ or "democratic republic of the congo"/ or cyprus/ or djibouti/ or dominica/ or dominican republic/ or ecuador/ or egypt/ or el salvador/ or equatorial guinea/ or eritrea/ or eswatini/ or ethiopia/ or fiji/ or gabon/ or gambia/ or "georgia (republic)"/ or ghana/ or grenada/ or guatemala/ or guinea/ or guinea-bissau/ or guyana/ or haiti/ or honduras/ or independent state of samoa/ or exp india/ or indian ocean islands/ or indochina/ or indonesia/ or iran/ or iraq/ or jamaica/ or jordan/ or kazakhstan/ or kenya/ or kosovo/ or kuwait/ or kyrgyzstan/ or laos/ or lebanon/ or liechtenstein/ or lesotho/ or liberia/ or libya/ or madagascar/ or malaysia/ or malawi/ or	159,916

	mali/ or malta/ or mauritania/ or mauritius/ or mekong valley/ or melanesia/ or micronesia/ or monaco/ or mongolia/ or montenegro/ or morocco/ or mozambique/ or myanmar/ or namibia/ or nepal/ or nicaragua/ or niger/ or nigeria/ or oman/ or pakistan/ or palau/ or exp panama/ or papua new guinea/ or paraguay/ or peru/ or philippines/ or qatar/ or "republic of belarus"/ or "republic of north macedonia"/ or romania/ or exp russia/ or rwanda/ or "saint kitts and nevis"/ or saint lucia/ or "saint vincent and the grenadines"/ or "sao tome and principe"/ or saudi arabia/ or serbia/ or sierra leone/ or senegal/ or seychelles/ or singapore/ or somalia/ or south africa/ or south sudan/ or sri lanka/ or sudan/ or suriname/ or syria/ or taiwan/ or tajikistan/ or tanzania/ or thailand/ or timor-leste/ or togo/ or tonga/ or "trinidad and tobago"/ or tunisia/ or turkmenistan/ or uganda/ or ukraine/ or united arab emirates/ or uruguay/ or uzbekistan/ or vanuatu/ or venezuela/ or vietnam/ or west indies/ or yemen/ or zambia/ or zimbabwe/	
S23	SU australasia/ or exp australia/ or austria/ or baltic states/ or belgium/ or exp canada/ or chile/ or colombia/ or costa rica/ or czech republic/ or exp denmark/ or estonia/ or europe/ or finland/ or exp france/ or exp germany/ or greece/ or hungary/ or iceland/ or ireland/ or israel/ or exp italy/ or exp japan/ or korea/ or latvia/ or lithuania/ or luxembourg/ or mexico/ or netherlands/ or new zealand/ or north america/ or exp norway/ or poland/ or portugal/ or exp "republic of korea"/ or "scandinavian and nordic countries"/ or slovakia/ or slovenia/ or spain/ or sweden/ or switzerland/ or turkey/ or exp united kingdom/ or exp united states/ OR European Union/ OR Developed Countries/	158,684
S24	S22 NOT S23	154,938
S25	S21 NOT S24	66

Appendix 4: Web of Science

#	Question	Results
#1	TS=(harm reduction OR risk reduction behavior OR (protective NEAR/2 strateg*) OR ((reduc* OR minimi*) NEAR/5 (harm OR harms OR harmful OR risk OR risks)))	329,523
#2	TS=(cannabis OR marijuana OR mari$uana OR hashish OR pot OR weed OR tetrahydrocannabinol OR THC OR CDB OR cannabidiol OR cannabinoid$)	305,377
#3	TS=(health personnel OR social worker$ OR counselor$ OR health occupation$ OR worker$ OR psychoeducator$ OR psycho-educator$ OR nurse$ OR criminologist$ OR psychologist$ OR clinician$ OR practitioner$ OR physician$ OR professional$ OR provider$ OR co$nselor OR co$nselors OR caregiver$ OR giver$ OR therapist$ OR psychotherapist$ OR staff$ OR personnel$ OR employee$ OR doctor$)	2,593,408
#4	#1 AND #2 AND #3	331
#5	#4 and English or French (Languages)	324
#6	#5 and [Countries/Regions filter: pays de l'OCDE]	300

Appendix 5: Embase

1	Harm Reduction/	8189
2	Risk Reduction Behavior/	115695
3	(protective adj2 strateg*).ab,kf,ti.	3987
4	((reduc* or minimi*) adj5 (harm? or harmful or risk?)).ab,kf,ti.	345174
**5**	1 or 2 or 3 or 4	414447
6	exp Health Personnel/	1843814
7	Social Workers/	12409
8	Counselors/	3692
9	exp Health Occupations/	24456
10	exp Allied Health Occupations/	392205
11	exp Allied Health Personnel/	567029
12	(worker? or (psychoeducator? or psycho-educator?) or educator? or nurse? or criminologist? or psychologist? or clinician? or practitioner? or physician? or professional? or provider? or co?nselor or co?nselors or caregiver? or giver? or therapist? or psychotherapist? or staff? or personnel? or employee? or doctor?).ab,kf,ti.	2640146
**13**	6 or 7 or 8 or 9 or 10 or 11 or 12	3761559
14	Cannabis/	40937
15	"Marijuana Use"/	12636
16	Marijuana Abuse/	6445
17	Marijuana Smoking/	4026
18	(mari?uana or cannabis or has?hish or Pot or weed or tetrahydrocannabinol or THC or CDB or cannabidiol or cannabinoid?).ab,kf,ti.	129932
**19**	14 or 15 or 16 or 17 or 18	142957
20	5 and 13 and 19	569
21	limit 32 to yr="1990 -Current"	569
22	limit 21 to (english or french)	560
23	Filtre OCDE	520

Appendix 6: Sociological Abstracts

(((ti(health personnel OR social worker OR social workers OR counselor OR counselors OR conselor OR conselors OR health occupation OR health occupations OR worker* OR psychoeducator* OR psycho-educator* OR educator* OR nurse* OR criminologist* OR psychologist* OR clinician* OR professional* OR provider* OR caregiver* OR giver* OR therapist* OR psychotherapist* OR staff* OR personnel* OR employee* OR doctor*) OR ab(health personnel OR social worker OR social workers OR counselor OR counselors OR conselor OR conselors OR health occupation OR health occupations OR worker* OR psychoeducator* OR psycho-educator* OR educator* OR nurse* OR criminologist* OR psychologist* OR clinician* OR professional* OR provider* OR caregiver* OR giver* OR therapist* OR psychotherapist* OR staff* OR personnel* OR employee* OR doctor*) OR su(health personnel OR social worker OR social workers OR counselor OR counselors OR conselor OR conselors OR health occupation OR health occupations OR worker* OR psychoeducator* OR psycho-educator* OR educator* OR nurse* OR criminologist* OR psychologist* OR clinician* OR professional* OR provider* OR caregiver* OR giver* OR therapist* OR psychotherapist* OR staff* OR personnel* OR employee* OR doctor*)) AND (ti(cannabis OR marijuana OR mari*uana OR hashish OR pot OR weed OR tetrahydrocannabinol OR THC OR CDB OR cannabidiol OR cannabinoid*) OR ab(cannabis OR marijuana OR mari*uana OR hashish OR pot OR weed OR tetrahydrocannabinol OR THC OR CDB OR cannabidiol OR cannabinoid*) OR su(cannabis OR marijuana OR mari*uana OR hashish OR pot OR weed OR tetrahydrocannabinol OR THC OR CDB OR cannabidiol OR cannabinoid*)) AND ((ti((reduc* OR minimi*) NEAR/5 (harm OR harms OR harmful OR risk OR risks)) OR ab((reduc* OR minimi*) NEAR/5 (harm OR harms OR harmful OR risk OR risks)) OR su((reduc* OR minimi*) NEAR/5 (harm OR harms OR harmful OR risk OR risks))) OR (ti(protective NEAR/2 strateg*) OR ab(protective NEAR/2 strateg*) OR su(protective NEAR/2 strateg*)))) AND la.exact("ENG")) AND yr(1990-2029)

Appendix 7: Google Scholar

cannabis intitle:nurse OR intitle:clinician OR intitle:practitioner OR intitle:physician OR intitle:caregiver OR intitle:giver OR intitle:doctor "harm reduction" = 105

cannabis intitle:nurses OR intitle:clinicians OR intitle:practitioners OR intitle:physicians OR intitle:caregivers OR intitle:givers OR intitle:doctors "harm reduction" = 242

cannabis intitle:psychoeducator OR intitle:educator OR intitle:therapist OR intitle:psychotherapist OR intitle:criminologist OR intitle:psychologist "harm reduction" = 19

cannabis intitle:psychoeducators OR intitle:educators OR intitle:therapists OR intitle:psychotherapists OR intitle:criminologists OR intitle:psychologists "harm reduction" = 53

cannabis intitle:worker OR intitle:staff OR intitle:personnel OR intitle:employee OR intitle:professional OR intitle:provider OR intitle:counselor OR intitle:conselor "harm reduction" = 136

cannabis intitle: workers OR intitle:staffs OR intitle:personnels OR intitle:employees OR intitle:professionals OR intitle:providers OR intitle:counselors OR intitle:conselors "harm reduction" = 5

cannabis intitle:infirmier OR intitle:médecin OR intitle:clinicien OR intitle:psychoéducateur OR intitle:éducateur OR intitle:thérapeute OR intitle:psychothérapeute OR intitle:criminologue OR intitle:psychologue “reduction des méfaits” = 1

cannabis intitle:travailleur OR intitle:personnel OR intitle:employé OR intitle:professionnel OR intitle:conseiller OR intitle:intervenant “reduction des méfaits” = 5

cannabis intitle:infirmiers OR intitle:médecins OR intitle:cliniciens OR intitle:psychoéducateurs OR intitle:éducateurs OR intitle:thérapeutes OR intitle:psychothérapeutes OR intitle:criminologues OR intitle:psychologues “reduction des méfaits” = 3

cannabis intitle:travailleurs OR intitle:personnels OR intitle:employés OR intitle:professionnels OR intitle:conseillers OR intitle:intervenants “reduction des méfaits” = 12

cannabis intitle:infirmière OR intitle:cliniciennne OR intitle:psychoéducatrice OR intitle:éducatrice OR intitle:criminologue OR intitle:psychologue “reduction des méfaits” = 2

cannabis intitle:travailleuse OR intitle:employée OR intitle:professionnelle OR intitle:conseillère OR intitle:intervenante “reduction des méfaits” = 1

cannabis intitle:infirmières OR intitle:cliniciennes OR intitle:psychoéducatrices OR intitle:éducatrices OR intitle:criminologues OR intitle:psychologues “reduction des méfaits” = 2

cannabis intitle:travailleuses OR intitle:employées OR intitle:professionnelles OR intitle:conseillères OR intitle:intervenantes “reduction des méfaits” = 2

marijuana intitle:nurse OR intitle:clinician OR intitle:practitioner OR intitle:physician OR intitle:caregiver OR intitle:giver OR intitle:doctor "harm reduction" = 139

marijuana intitle:nurses OR intitle:clinicians OR intitle:practitioners OR intitle:physicians OR intitle:caregivers OR intitle:givers OR intitle:doctors "harm reduction" = 238

marijuana intitle:psychoeducator OR intitle:educator OR intitle:therapist OR intitle:psychotherapist OR intitle:criminologist OR intitle:psychologist "harm reduction" = 19

marijuana intitle:psychoeducators OR intitle:educators OR intitle:therapists OR intitle:psychotherapists OR intitle:criminologists OR intitle:psychologists "harm reduction" = 48

marijuana intitle:worker OR intitle:staff OR intitle:personnel OR intitle:employee OR intitle:professional OR intitle:provider OR intitle:counselor OR intitle:conselor "harm reduction" = 140

marijuana intitle:workers OR intitle:staffs OR intitle:personnels OR intitle:employees OR intitle:professionals OR intitle:providers OR intitle:counselors OR intitle:conselors "harm reduction" = 573

marijuana intitle:infirmier OR intitle:médecin OR intitle:clinicien OR intitle:psychoéducateur OR intitle:éducateur OR intitle:thérapeute OR intitle:psychothérapeute OR intitle:criminologue OR intitle:psychologue “reduction des méfaits” = 1

marijuana intitle:travailleur OR intitle:personnel OR intitle:employé OR intitle:professionnel OR intitle:conseiller OR intitle:intervenant “reduction des méfaits” = 5

marijuana intitle:infirmiers OR intitle:médecins OR intitle:cliniciens OR intitle:psychoéducateurs OR intitle:éducateurs OR intitle:thérapeutes OR intitle:psychothérapeutes OR intitle:criminologues OR intitle:psychologues “reduction des méfaits” = 3

marijuana intitle:travailleurs OR intitle:personnels OR intitle:employés OR intitle:professionnels OR intitle:conseillers OR intitle:intervenants “reduction des méfaits” = 12

marijuana intitle:infirmière OR intitle:cliniciennne OR intitle:psychoéducatrice OR intitle:éducatrice OR intitle:criminologue OR intitle:psychologue “reduction des méfaits” = 2

marijuana intitle:travailleuse OR intitle:employée OR intitle:professionnelle OR intitle:conseillère OR intitle:intervenante “reduction des méfaits” = 1

marijuana intitle:infirmières OR intitle:cliniciennes OR intitle:psychoéducatrices OR intitle:éducatrices OR intitle:criminologues OR intitle:psychologues “reduction des méfaits” = 2

marijuana intitle:travailleuses OR intitle:employées OR intitle:professionnelles OR intitle:conseillères OR intitle:intervenantes “reduction des méfaits” = 2

Appendix 8: Google Web

cannabis intitle:nurse OR intitle:clinician OR intitle:practitioner OR intitle:physician OR intitle:caregiver OR intitle:giver OR intitle:doctor "harm reduction" filetype:pdf =

cannabis intitle:nurses OR intitle:clinicians OR intitle:practitioners OR intitle:physicians OR intitle:caregivers OR intitle:givers OR intitle:doctors "harm reduction" filetype:pdf =

cannabis intitle:psychoeducator OR intitle:educator OR intitle:therapist OR intitle:psychotherapist OR intitle:criminologist OR intitle:psychologist "harm reduction" filetype:pdf =

cannabis intitle:psychoeducators OR intitle:educators OR intitle:therapists OR intitle:psychotherapists OR intitle:criminologists OR intitle:psychologists "harm reduction" filetype:pdf =

cannabis intitle:worker OR intitle:staff OR intitle:personnel OR intitle:employee OR intitle:professional OR intitle:provider OR intitle:counselor OR intitle:conselor "harm reduction" filetype:pdf =

cannabis intitle: workers OR intitle:staffs OR intitle:personnels OR intitle:employees OR intitle:professionals OR intitle:providers OR intitle:counselors OR intitle:conselors "harm reduction" filetype:pdf =

cannabis intitle:infirmier OR intitle:médecin OR intitle:clinicien OR intitle:psychoéducateur OR intitle:éducateur OR intitle:thérapeute OR intitle:psychothérapeute OR intitle:criminologue OR intitle:psychologue “reduction des méfaits” filetype:pdf =

cannabis intitle:travailleur OR intitle:personnel OR intitle:employé OR intitle:professionnel OR intitle:conseiller OR intitle:intervenant “reduction des méfaits” filetype:pdf =

cannabis intitle:infirmiers OR intitle:médecins OR intitle:cliniciens OR intitle:psychoéducateurs OR intitle:éducateurs OR intitle:thérapeutes OR intitle:psychothérapeutes OR intitle:criminologues OR intitle:psychologues “reduction des méfaits” filetype:pdf =

cannabis intitle:travailleurs OR intitle:personnels OR intitle:employés OR intitle:professionnels OR intitle:conseillers OR intitle:intervenants “reduction des méfaits” filetype:pdf =

cannabis intitle:infirmière OR intitle:clinicienne OR intitle:psychoéducatrice OR intitle:éducatrice OR intitle:criminologue OR intitle:psychologue “reduction des méfaits” filetype:pdf =

cannabis intitle:travailleuse OR intitle:employée OR intitle:professionnelle OR intitle:conseillère OR intitle:intervenante “reduction des méfaits” filetype:pdf =

cannabis intitle:infirmières OR intitle:cliniciennes OR intitle:psychoéducatrices OR intitle:éducatrices OR intitle:criminologues OR intitle:psychologues “reduction des méfaits” filetype:pdf =

cannabis intitle:travailleuses OR intitle:employées OR intitle:professionnelles OR intitle:conseillères OR intitle:intervenantes “reduction des méfaits” filetype:pdf =

marijuana intitle:nurse OR intitle:clinician OR intitle:practitioner OR intitle:physician OR intitle:caregiver OR intitle:giver OR intitle:doctor "harm reduction" filetype:pdf =

marijuana intitle:nurses OR intitle:clinicians OR intitle:practitioners OR intitle:physicians OR intitle:caregivers OR intitle:givers OR intitle:doctors "harm reduction" filetype:pdf =

marijuana intitle:psychoeducator OR intitle:educator OR intitle:therapist OR intitle:psychotherapist OR intitle:criminologist OR intitle:psychologist "harm reduction" filetype:pdf =

marijuana intitle:psychoeducators OR intitle:educators OR intitle:therapists OR intitle:psychotherapists OR intitle:criminologists OR intitle:psychologists "harm reduction" filetype:pdf =

marijuana intitle:worker OR intitle:staff OR intitle:personnel OR intitle:employee OR intitle:professional OR intitle:provider OR intitle:counselor OR intitle:conselor "harm reduction" filetype:pdf =

marijuana intitle:workers OR intitle:staffs OR intitle:personnels OR intitle:employees OR intitle:professionals OR intitle:providers OR intitle:counselors OR intitle:conselors "harm reduction" filetype:pdf =

marijuana intitle:infirmier OR intitle:médecin OR intitle:clinicien OR intitle:psychoéducateur OR intitle:éducateur OR intitle:thérapeute OR intitle:psychothérapeute OR intitle:criminologue OR intitle:psychologue “reduction des méfaits” filetype:pdf =

marijuana intitle:travailleur OR intitle:personnel OR intitle:employé OR intitle:professionnel OR intitle:conseiller OR intitle:intervenant “reduction des méfaits” filetype:pdf =

marijuana intitle:infirmiers OR intitle:médecins OR intitle:cliniciens OR intitle:psychoéducateurs OR intitle:éducateurs OR intitle:thérapeutes OR intitle:psychothérapeutes OR intitle:criminologues OR intitle:psychologues “reduction des méfaits” filetype:pdf =

marijuana intitle:travailleurs OR intitle:personnels OR intitle:employés OR intitle:professionnels OR intitle:conseillers OR intitle:intervenants “reduction des méfaits” filetype:pdf =

marijuana intitle:infirmière OR intitle:cliniciennne OR intitle:psychoéducatrice OR intitle:éducatrice OR intitle:criminologue OR intitle:psychologue “reduction des méfaits” filetype:pdf =

marijuana intitle:travailleuse OR intitle:employée OR intitle:professionnelle OR intitle:conseillère OR intitle:intervenante “reduction des méfaits” filetype:pdf =

marijuana intitle:infirmières OR intitle:cliniciennes OR intitle:psychoéducatrices OR intitle:éducatrices OR intitle:criminologues OR intitle:psychologues “reduction des méfaits” filetype:pdf =

marijuana intitle:travailleuses OR intitle:employées OR intitle:professionnelles OR intitle:conseillères OR intitle:intervenantes “reduction des méfaits” filetype:pdf =

Appendix 9: BASE

tit:(nurse* clinician* practitioner* physician* caregiver* giver* doctor* psychoeducator* educator* therapist* psychotherapist* criminologist* psychologist* worker* staff* personnel* employee* professional* provider* counselor*) subj:(cannabis marijuana) subj:"harm reduction" = 4

tit:(infirmier* OU médecin* OU clinicien* OU psychoéducat* OU éducat* OU criminologue* OU psychologue* OU travailleu* OU employé* OU professionnel* OU conseill* OU intervenant*) subj:cannabis subj:marijuana subj:"réduction des méfaits" = 0

Appendix 10: Érudit

(Titre, résumé, mots-clés : harm reduction OU harm minimisation OU risk reduction OU risks reduction OU risk minimisation OU réduction des méfaits OU réduction des risques) ET (Titre, résumé, mots-clés : cannabis OU marijuana) ET (Titre, résumé, mots-clés : nurse* OU clinician* OU practitioner* OU physician* OU caregiver* OU giver* OU doctor* OU psychoeducator* OU educator* OU therapist* OU psychotherapist* OU criminologist* OU psychologist* OU worker* OU staff* OU personnel* OU employee* OU professional* OU provider* OU counselor* OU infirmier* OU médecin* OU clinicien* OU psychoéducat* OU éducat* OU criminologue* OU psychologue* OU travailleu* OU employé* OU professionnel* OU conseill* OU intervenant*) = 27
