# Supplementary material for: Facilitators of and barriers to healthcare providers’ adoption of harm reduction in cannabis use: a scoping review protocol
Source: BMJ Open. 2024 Apr 5;14(4):e078427. doi: 10.1136/bmjopen-2023-078427 (PMC11002399; doi:10.1136/bmjopen-2023-078427)
Supplement: Author's manuscript [file bmjopen-2023-078427.draft_revisions.pdf]

BMJ Open

BMJ Open is committed to open peer review. As part of this commitment we make the peer review history of every article we publish publicly available.

When an article is published we post the peer reviewers' comments and the authors' responses online. We also post the versions of the paper that were used during peer review. These are the versions that the peer review comments apply to.

The versions of the paper that follow are the versions that were submitted during the peer review process. They are not the versions of record or the final published versions. They should not be cited or distributed as the published version of this manuscript.

BMJ Open is an open access journal and the full, final, typeset and author-corrected version of record of the manuscript is available on our site with no access controls, subscription charges or pay-per-view fees (http://bmjopen.bmj.com).

If you have any questions on BMJ Open's open peer review process please email info.bmjopen@bmj.com

BMJ Open

Facilitators and barriers to healthcare providers' adoption of the harm reduction approach to cannabis use: a scoping review protocol

Journal:	*BMJ Open*
Manuscript ID	bmjopen-2023-078427
Article Type:	Protocol
Date Submitted by the Author:	01-Aug-2023

Complete List of Authors:	Haddad, Roula; University of Montreal, Psychology<br>Dagenais, Christian; University of Montreal, Psychology<br>Huynh, Christophe ; University of Montreal, Psychiatry and addictology<br>Fallu, Jean-Sébastien; University of Montreal, Psychoeducation

Keywords:	PUBLIC HEALTH, MENTAL HEALTH, Substance misuse < PSYCHIATRY

SCHOLARONE™
Manuscripts

Facilitators and barriers to healthcare providers' adoption of the harm reduction approach to cannabis use: a scoping review protocol

Roula Haddad[1], Christian Dagenais[1], Christophe Huynh[2], Jean-Sébastien Fallu[3]

[1] Department of Psychology, University of Montreal

[2] Department of Psychiatry and Addictology, University of Montreal

[3] School of Psychoeducation, University of Montreal

Correspondence to Roula Haddad; roula.haddad@umontreal.ca

ABSTRACT

Introduction. The high prevalence of cannabis use and the potential for negative effects indicate the need for effective prevention strategies and treatment of people who use cannabis. Studies show that the harm reduction (HR) approach to cannabis use is effective in minimizing the harmful consequences of the substance. However, health professionals often misunderstand it and resist its adoption due to various obstacles and barriers. To our knowledge, to date there has been no review of the scientific literature on the factors that facilitate or hinder practitioners' adoption of HR in cannabis use. To fill this gap, we aim to identify, through a scoping review, facilitators and barriers to healthcare providers' adoption of HR in cannabis use in Organisation for Economic Co-operation and Development countries.

Methods and analysis. Our methodology will be guided by the six-step model initially proposed by Arksey and O'Malley (2005). Our search strategy will be executed on different databases and the grey literature will also be searched. Key journals related to the field of study and reference lists of selected articles will be searched manually to identify any publications missed by the electronic searches. Relevant systematic reviews will be scanned to identify the studies included. Finally, the search strategy will be supplemented by contacting authors, existing networks, and organizations. All study designs will be included, and the selected studies will be those presenting factors that facilitate or hinder healthcare providers' adoption of HR in cannabis use.

For peer review only

Ethics and dissemination. Ethics approval is not required. The results will be disseminated through various activities and will be used to develop and implement a knowledge translation process among healthcare practitioners working in addiction rehabilitation services in Quebec with adolescents and young adults. This process will mainly aim to change practitioners' practices and improve their adoption of HR in cannabis use.

Strengths and limitations of this study

- To our knowledge, this scoping review will be the first to identify factors that facilitate or hinder healthcare providers' adoption of HR in cannabis use. Other reviews have studied other HR interventions among practitioners with the adult population.
- The search strategy was co-developed by two information management specialists in the addiction and knowledge translation fields.
- Two reviewers will independently select the studies to be included in the scoping review.
- Even though not mandatory, the methods employed in the selected studies will be subject to a quality assessment using the Mixed Methods Appraisal Tool grid.
- The included studies will be limited to those published in French or English.

Key words: *harm reduction; cannabis; knowledge translation; healthcare*

For peer review only

INTRODUCTION

Cannabis use

Psychoactive substances (e.g., cannabis, alcohol, nicotine) are defined as substances whose use affects mental processes (e.g., perception, cognition, emotions, and mood) and behaviors without necessarily leading to addiction (1). After tobacco and alcohol, cannabis represents the third most consumed psychoactive substance globally among adults and youth (2-4). Since the 2000s, rates of cannabis use in Canada and Quebec have been declining (5). Despite this change, Canada is still among the developed countries with the highest rates of cannabis use among adolescents and young adults, with high prevalence in Quebec and multiple patterns of use (e.g., smoking, eating, vaporizing, vaping) (2, 5-7). In 2022, 27% of those 16 years of age and older reported using cannabis in the 12 months prior to the study (8). Among young people with psychosocial adjustment difficulties admitted to Quebec's foster youth centers, 78% have used cannabis at some time, and nearly half presented a problematic use (5, 9). Cannabis use does not necessarily lead to dependence due to its limited addictive power, infrequent use by most people who use it, and minimal consequences at low rates of use (10, 11). However, harm may sometimes occur at the personal (e.g., decreased academic performance), health (e.g., overuse of drugs and deterioration of mental health), behavioral (e.g., delinquency and risky behaviors), psychosocial, and legal levels (3, 4, 11-13). These consequences, along with the high prevalence of cannabis use, make it essential to implement intervention programs among young people who use cannabis (5).

Abstinence approach

To address this reality, prevention and treatment programs have been developed based on the abstinence approach, which entails total elimination of substance use (10, 14). The emergence of these programs has been also influenced by policies such as the "War on Drugs" (15). The abstinence approach forms the basis of most programs developed to prevent or treat problematic substance use among adolescents and young adults and has been applied with at-risk or marginalized populations (e.g., youth in the foster care system) (13). Despite its potential to decrease the frequency or amount of substance use, the abstinence approach with youth has been criticized for various reasons (13). First, it does not provide them with the necessary skills to identify and mitigate the harms associated with their use (13, 16). Second, the abstinence approach focuses on the negative consequences of use through strategies that evoke fear in people who use

For peer review only

psychoactive substances and bypasses key factors contributing to substance use, such as peer pressure and social context (12, 13). Third, the risks of relapse and dropout among adolescents and youth in these programs are also found to be high: only one-third of this population manages to eliminate any kind of substance use during treatment, leaving a significant number for whom this goal remains unattainable (12, 17). Given these limitations of abstinence-oriented programs, other alternative and more flexible treatments that are acceptable to youth, such as harm reduction (HR), are essential (17, 18, 19).

Harm reduction (HR)

Description

HR in cannabis use aims to minimize the harmful consequences of the substance at the individual, psychological, legal, and social levels among users (12, 17, 18). It offers a public health framework based on values of pragmatism and humanism, as it does not view substance use through a moral lens, but as an inevitable societal fact of long standing (3, 10, 20, 21). Whether among adults or adolescents, elimination of substance use is unrealistic at the population level and should be the individual's choice without being imposed, as it represents an unwanted and impractical goal for some (e.g., recreational/occasional users, or in cases of dual diagnosis combining psychiatric and substance use disorders) (16-18, 22, 23). HR also seeks to equip people who use substances to make responsible and rational decisions and learn ways to reduce the negative consequences associated with use (10-12, 16, 18, 21). To this end, it clarifies the notion of safe substance use that is determined by the interaction of three components: the individual (height, weight, gender, physical and mental health status, state of mind, etc.); the drug (quantity, frequency of use, tolerance to the product, combination with other products, quality, etc.); and the setting (location, time of day, interpersonal relationships, conflicts, laws, etc.) (3, 12, 16). Unlike adults, adolescents and young adults are often referred for treatment by their parents, school, or the juvenile justice system, which may limit their internal motivation to change their substance use behaviors (12, 18). In such cases, HR takes into account their personal characteristics (impulsivity, sensation-seeking, etc.) and addresses their ambivalence about stopping substance use, feelings of failure upon relapse, decision-making, goal-setting, engagement in treatment, social skills, emotional regulation, etc. (12).

For peer review only

HR efficacy

Interventions based on HR for non-injected drugs have been most widely studied among adults (for example, in housing programs among people diagnosed with substance use disorders and psychiatric disabilities) and have shown promising results in decreasing the negative consequences associated with substance use (12, 13, 17). However, among adolescents and young adults, the focus is more on school-based programs that combine preventive strategies, early interventions, and HR practices to reduce substance use (4, 6). Additionally, most studies have focused on alcohol, and few have focused on cannabis or the implementation and effectiveness of intensive treatment based on HR in this population (4, 6, 12). Indeed, current school-based HR programs (e.g., SHAHRP in the United Kingdom and SCIDUA in Canada) are effective in developing safer attitudes toward substance use and reducing negative consequences related to use (13, 24, 25). A study by the Academic Institute on Addictions (*Institut universitaire sur les dépendances*) in Quebec as part of the IP-Youth project confirms that "effective early interventions targeting college and university students with at-risk cannabis use" are those that reduce the harms associated with cannabis use (e.g., Computerized Brief Intervention for Cannabis, eCHECKUP TO GO - Marijuana) (26-29). As a result, researchers have suggested integrating HR into interventions targeting adolescents and young adults (28).

Acceptability of harm reduction

Despite its proven effectiveness, the acceptability of HR among healthcare providers remains controversial, and various factors facilitate or hinder its adoption (30, 31). MacCoun (32) showed that, among practitioners who did not adopt an HR approach to substance use (i.e., not limited to cannabis), some had based their decision on moral grounds, regardless of its effectiveness. Various barriers limit its use by practitioners. First, ambiguities in its conceptualization play an influential role; for example, some practioners perceive HR as sending the wrong message, i.e., one of tolerating or even encouraging substance use (16, 17, 20, 33). Some do not perceive total cessation of substance use as a legitimate goal that could be achieved through HR (34). As well, there is often confusion between reducing use (frequency, quantity, etc.) and reducing harm (modifying consumption practices, such as contexts and mixtures, to reduce consequences) (20). These misconceptions point to the need for awareness-raising, training, and supervision of practitioners interested in this approach (17). Second, the adoption of HR can be hindered by ethical dilemmas,

For peer review only

as well as by issues related to the personal and collective values of healthcare workers and the therapeutic model of abstinence (20). Indeed, it runs counter to traditional treatments by tolerating risky behaviors and accepting that HR in drug use is a legitimate outcome (20, 33). Practitioners may also fear the emergence of legal, social, and health problems among their clients (17). Third, its adoption may also be limited by contextual barriers, such as lack of funding, stigma that undermines demand for care, resistance from local jurisdictions, and lack of services and trained personnel, particularly in the mental health sector (30).

However, several factors seen as HR benefits have been found to facilitate implementation by healthcare providers, such as: broadening the spectrum of acceptable goals, improving clients' decision-making skills, creating positive and quality relationships, and managing relapses (17). A study by Sharp et al. (2020) showed that clarifying the positive impacts of HR at the community level (e.g., safety) and ensuring the availability of resources could increase the likelihood of its adoption.

Purpose

Despite its proven effectiveness, HR remains under-implemented, especially among youth, due to health professionals' resistance (20, 30). However, to date there has been no review of the scientific literature that identifies facilitators and barriers to the adoption of HR in cannabis use. To fill this gap, we aim to identify, through a scoping review, facilitators and barriers to healthcare providers' adoption of HR in cannabis use.

METHODS AND ANALYSIS

This study will follow the methodological steps of scoping reviews (35). This type of review has become more prevalent in recent years and is a type of knowledge synthesis review (35, 36). There is no universal definition for scoping reviews; however, a variety of factors distinguish them from other types of knowledge synthesis (37). First, scoping reviews address broad research questions and include studies with different designs and multiple sources of evidence to provide an overview of the available knowledge around a concept (38). A systematic review following Cochrane standards, on the other hand, explores more specific research questions based on detailed inclusion and exclusion criteria (39-41). Second, while assessment of the methodological quality of included studies is recommended for scoping reviews, it is not mandatory, whereas assessment of the risk

For peer review only

of bias of included studies is required for Cochrane-type systematic reviews (39, 41). Researchers undertake scoping reviews for a variety of reasons: 1) to review research activity in a given area; 2) to determine the feasibility and appropriateness of conducting a systematic review based on Cochrane standards; 3) to summarize and disseminate the results of existing research on a topic; and/or 4) to identify a gap in the literature and draw conclusions regarding a topic (38). Our methodological choice is underpinned by three of these reasons: once the research activity around the topic has been consulted, the findings will be summarized and used to support a second study aimed at disseminating knowledge to practitioners through a knowledge translation process. This will also allow us to identify gaps in the literature and draw conclusions related to the topic.

Arksey and O'Malley (2005) were the first to propose a six-step model for conducting scoping reviews. Our methodology will be guided by this model, which was later refined by Levac et al. (2010) and revised by members of the Johanna Briggs Institute (39). The six stages we will follow are:

- Stage 1: Determining the research question and the objective
- Stage 2: Identifying relevant studies
- Stage 3: Selecting studies
- Stage 4: Charting the data
- Stage 5: Collating, summarizing, and reporting the results
- Stage 6: Conducting a consultation exercise

The research protocol will be reported using the Preferred Reporting Items for Systematic Reviews and Meta-Analyses Extension for Scoping Reviews (PRISMA-ScR) grid (36). This grid is an extension of the PRISMA grid originally developed for Cochrane-type systematic reviews and helps to ensure the transparency and reproducibility of the study (36, 39).

Stage 1: Determining the research question and the objective

Arksey and O'Malley's (2005) model suggests that scoping reviews should begin not only with identifying research questions, but also clarifying the resulting objectives (37, 39). Our scoping review is exploratory in nature and aims to identify facilitators and barriers to healthcare providers' adoption of HR in cannabis use in OECD countries. Based on the Population–Concept–Context (PCC) model, which allows the broad scope of the study to be respected without specifying restrictive inclusion criteria (39), we formulated the research question: What factors influence

For peer review only

providers (population) in the healthcare field (context) to adopt HR in cannabis use (concept)? Specific research questions associated with the components of the PCC model were also identified.

- Question 1, related to the concept component: What is the definition of HR in cannabis use?
- Question 2, related to the concept and context components: What are the facilitators and barriers to healthcare providers' adoption of HR in cannabis use?
- Question 3, related to the population component: Who are the clientele of the providers identified in the studies (e.g., adolescents and young adults, pregnant women, individuals with psychotic disorders, etc.)?
- Question 4, related to the concept component: What are the gaps in the literature and the future research needs?

Stage 2: Identifying relevant studies

Search strategy

The search strategy was developed through an iterative process. A senior librarian at the Quebec Addiction Library (*Bibliothèque québécoise sur les dépendances*) first developed three search strategies with different concepts and ran them on the Medline database. We consulted the first 50 results of each strategy and chose the one that grouped key terms related to the following concepts: harm reduction, clinicians, and cannabis (see Supplemental Appendix 1). The search strategy was then reviewed by a second information professional working in the RENARD Team for Knowledge Translation who, in turn, adapted it to the selected databases. The final search strategy executed on all the databases was then validated by the RENARD Team information specialist. The Peer Review of Electronic Search Strategies (PRESS) tool served as a guide for the librarians in this process (42). The search strategy executed on Medline and adapted for the other selected databases is presented in Supplemental Appendix 1.

Information sources

To identify relevant published and unpublished studies for inclusion in the study, various sources of information will be reviewed (35, 38, 39). With the guidance of the two librarians, the search strategy will be executed on the leading health and intervention databases: Medline, PsycINFO, CINAHL, Web of Science, Embase, and Sociological Abstracts. To explore the grey literature

(e.g., theses, research reports, etc.), the search strategy will be adapted to the Google Web and Google Scholar search engines, as well as the Érudit (French database) and BASE databases. All documents identified will be entered into Zotero software for the research team members to access. Key journals related to the field of study (e.g., *Harm Reduction Journal*; *International Journal of Drug Policy*) and reference lists of selected articles will be manually searched to identify any publications missed by the electronic searches. Systematic reviews will be scanned to identify studies included. Finally, the search strategy will be supplemented by contacting authors, existing networks, and organizations.

Stage 3: Study selection

After running the search strategy on the selected databases and completing the second stage, all identified duplicates will be removed. The remaining documents will then be entered into Covidence software. Two reviewers will independently select relevant documents for inclusion by reading the titles and abstracts of the identified studies. Their decisions will be based on the specified inclusion and exclusion criteria. They will meet regularly to resolve selection conflicts and refine the eligibility criteria as needed. After this first step, the inter-rater agreement between the reviewers will be calculated. The documents selected and deemed potentially relevant will then be the subject of the second step, the full-text reading. Again, the two reviewers will independently record their choices on Covidence and resolve any new conflicts, and the inter-rater agreement for this step will then be calculated. A third reviewer will be called upon as needed for any conflict resolution. This will complete the document selection stage, whose steps will be presented in a PRISMA diagram (43).

Inclusion and exclusion criteria

Inclusion and exclusion criteria have been specified and will be fine-tuned as needed to select relevant studies (Table 1). Using this process, we will select empirical studies of quantitative, qualitative, or mixed designs, including from the grey literature, and will focus on projects based on identifying factors that facilitate or hinder healthcare providers' adoption of HR in cannabis use. To facilitate comparison and generalization of the results to the Quebec context, the review will be limited to studies conducted in any of the 38 Organisation for Economic Co-operation and Development (OECD) countries. Articles published from 1990 onwards will be included, as it was

For peer review only

early in that decade that HR gained international prominence and its scope of application began to expand. Papers not meeting these inclusion criteria will be excluded. While systematic reviews will be excluded to avoid duplication and ensure equal representation of the selected papers, their reference lists will nevertheless be examined to identify additional relevant studies.

Table 1. Inclusion and exclusion criteria

Criteria	Inclusion criteria	Exclusion criteria
**Type of study**	- Empirical study: quantitative, qualitative, or mixed	- Study that does not present empirical results (e.g., theoretical study, conceptual framework, etc.) or knowledge review (e.g., systematic or literature review)<br>- Interviews
**Type of documents**	- Peer-reviewed scientific articles, research reports, dissertations, theses	- Books and practice guides
**Conceptual framework**	- Harm reduction (HR) in cannabis use<br>- Cannabis risk reduction<br>- Non-abstinence in cannabis use	- Another conceptual framework
**Objective**	- Identification of factors[1] facilitating or hindering practitioners' adoption of the HR approach[2] in cannabis use	- Evaluation of the effectiveness of interventions based on HR<br>OR<br>- Stakeholder perceptions of the use of cannabis as an HR strategy to circumvent the effects of other drugs<br>OR<br>- Attitudes toward decriminalization of cannabis
**Psychoactive substance being studied**	- Marijuana, hashish, or cannabis for non-medical purposes<br>- "Drug" if cannabis is part of its conceptualization in the study	- Any substance other than marijuana, hashish, or non-medical cannabis (e.g., tobacco, alcohol, medical cannabis, MDMA, Ecstasy)<br>- Study that focuses on "performance and image enhancing drugs" or "crack" or "new psychoactive substances"
**Target population**	- Practitioners[3] working in the health field<br>- Pre-practitioners (in training to become practitioners)	- People who use cannabis[4]

**Country of study**	- OECD countries	- Other countries
**Publication date**	- From 1990 onwards	- Before 1990
**Language**	- French and/or English	- Languages other than French or English or text not available

[1] "Factors" include perceptions, beliefs, facilitators, obstacles, oppositions, attitudes, opinions, barriers, biases, motivations, preferences, determinants, incentives, influences, and perspectives on the adoption of HR in cannabis use, as well as its acceptability and receptivity.

[2] "Approach" refers to strategies, interventions, practices, services, methods, techniques, treatments, programs, or guides for the HR approach in cannabis use.

[3] "Practitioners" includes healthcare personnel, professionals, or practitioners, allied healthcare personnel, professionals, or practitioners, social workers, counsellors, psychoeducators, educators, nurses, criminologists, psychologists, clinicians, caregivers, therapists, psychotherapists, and physicians.

[4] Studies addressing the views of people who use cannabis regarding HR or its adoption by practitioners will be excluded.

Stage 4: Charting the data

To analyze the selected studies on a common basis, specific variables of interest will be identified based on the research questions (38). These will form the components of summary sheets that will be developed in Microsoft Excel and used to extract results (Table 2). This method is an analytical descriptive recording of the data (38, 39, 44). The first author (RH) will extract the data from the included studies and create the summary sheets. The research supervisor (CD) will validate the summary sheets throughout the process and ensure their alignment with the research questions (39). At this stage, the quality of the methods employed in the selected studies will be assessed using the Mixed Methods Appraisal Tool grid (45).

Table 2. Summary sheets

General variables	Specific variables
**General characteristics of the study**	Study title<br>Author(s)<br>Language of publication<br>Date of publication<br>Period of publication<br>Journal<br>Type of article<br>Full reference

For peer review only

	Country of study<br>Psychoactive substance under study<br>Legal status of cannabis in country of study
**Introduction**	Main concepts<br>Definition of the main concept: HR in cannabis use<br>Research question(s)<br>Objective(s)<br>Hypothesis
**Methodology**	Study design<br>Target population<br>Place of work of the target population<br>Inclusion criteria of participants<br>Recruitment method<br>Sample size<br>Country of origin of participants<br>Clientele of the population recruited<br>Data collection method<br>Analysis steps
**Results**	Sample presentation<br>Key findings: 1) facilitators and 2) barriers to practitioners' adoption of HR in cannabis use<br>Secondary outcomes or other results
**Conclusion**	Study strengths<br>Study limitations<br>Gaps in the literature and future research needs

Stage 5: Collating, summarizing, and reporting the results

Based on the eligibility criteria, studies deemed relevant will be collected, summarized, and reported. They will be subjected to: 1) a numerical analysis, and 2) a narrative organization encompassing a descriptive qualitative analysis (36, 38, 39). A numerical analysis of the scope, nature, and distribution of the included studies will be performed in relation to various characteristics: date of publication, country of origin of the studies, and type of document. Subsequently, a narrative organization of the results will be produced to identify the relationships between the data and the research questions. The summary sheets will be combined, tabulated, and synthesized, and will then be subjected to a descriptive qualitative analysis (Table 3).

For peer review only - http://bmjopen.bmj.com/site/about/guidelines.xhtml

Table 3. Narrative organization of the included studies

Data	Study 1	Study 2	Study …
Type of publication			
Date of publication			
Country of study			
Legal status of cannabis in the country of the study			
Definition of HR to cannabis use			
Design of the study			
Target population			
Place of work of the target population			
Clientele of the target population			
Data collection method			
Key findings:<br>- Facilitators or enabling conditions<br>- Barriers or adverse conditions			
Secondary outcomes			

Stage 6: Consultation exercise

Expert consultation is an optional step that promotes methodological rigor in scoping reviews (38). In this study, the project's supervisor and co-researchers will be solicited as consultants. Members of the RENARD team and researchers involved in the field of substance use and harm reduction will be consulted to help clarify findings and validate the resulting recommendations (38). Consultations will be conducted: 1) after preliminary results have been obtained, and 2) after analyses of the results have been completed.

ETHICS AND DISSEMINATION

To our knowledge, this is the first scoping review on factors that facilitate or hinder healthcare providers' adoption of HR in cannabis use. This study will provide a clear picture of the factors at play when adopting HR, and the results could potentially be generalizable to OECD countries. The present study is exempt from ethics approval because it involves no patient or personal data collection. The results are expected to be ready by March 2024. After completing the scoping review, we will be able to refine the knowledge translation plan that we aim to implement among practitioners working in addiction rehabilitation services in Quebec with adolescents (12–17 years)

For peer review only - http://bmjopen.bmj.com/site/about/guidelines.xhtml

and young adults (18–21 years) presenting psychosocial adjustment difficulties. The goal of our knowledge translation process will be mainly to change practitioners' practices and expand the adoption of HR in cannabis use.

Acknowledgments. We wish to thank Karine Bélanger, senior librarian at the *Bibliothèque québécoise sur les dépendances* in Quebec for developing the initial search strategy used in this scoping review. We also wish to thank Julie Desnoyers, the information specialist affiliated with the RENARD Team for Knowledge Translation at Université de Montréal, for validating the search strategy and adapting it to the selected databases. Finally, we want to thank Donna Riley for editing the manuscript.

Authors' contributions. RH, CD, CH, and JSF conceptualized the study. RH drafted the protocol. CD, CH, and JSF critically revised the manuscript. RH wrote the final draft manuscript, and all the authors approved it.

Funding statement. This work was supported by the *Ministère de la Santé et des Services sociaux* (MSSS), *Fonds de recherche du Québec – Santé* (FRQS), and *Fonds de recherche du Québec – Société et culture* (FRQSC) (Grant number: 2023-0PTR-322652).

Competing interests statement. None declared.

For peer review only

FULL REFERENCES

1. World Health Organization. Drugs (psychoactive) [online]; 2022. https://www.who.int/health-topics/drugs-psychoactive#tab=tab_1 (accessed 13 Jul 2023).

2. Adinoff B, Cooper ZD. Cannabis legalization: progress in harm reduction approaches for substance use and misuse. Am J Drug Alcohol Abuse 2019;45(6):707–12. doi: 10.1080/00952990.2019.1680683

3. Melamede R. Harm reduction-the cannabis paradox. Harm Reduct J 2005;2(1):17. doi: 10.1186/1477-7517-2-17

4. Jiloha RC. Prevention, early intervention, and harm reduction of substance use in adolescents. Indian J Psychiatry 2017;59(1):111–8. doi: 10.4103%2F0019-5545.204444

5. Gouvernement du Québec. Plan d'action interministériel en dépendances 2018-2028 – Prévenir, réduire et traiter les conséquences associées à la consommation de substances psychoactives, à la pratique de jeux de hasard et d'argent et à l'utilisation d'Internet [online]. 2018. https://publications.msss.gouv.qc.ca/msss/document-002078/ (accessed 13 Jul 2023).

6. Poulin C, Elliott D. Alcohol, tobacco and cannabis use among Nova Scotia adolescents: implications for prevention and harm reduction. CMAJ 1997;156(10):1387–93. PMID: **9164396**

7. Zuckermann AM, Gohari MR, Romano I, et al. Changes in cannabis use modes among Canadian youth across recreational cannabis legalization: data from the COMPASS prospective cohort study. Addict Behav 2021;122:107025. doi: 10.1016/j.addbeh.2021.107025

8. Government of Canada. Canadian Cannabis Survey 2022: Summary [online]. 2022. https://www.canada.ca/en/health-canada/services/drugs-medication/cannabis/research-data/canadian-cannabis-survey-2022-summary.html (accessed 13 Jul 2023).

9. Laventure M, Déry M, Pauzé R. Profils de consommation d'adolescents, garçons et filles, desservis par des centres jeunesse [Consumption profiles of adolescents (boys and girls) who receive services from youth centres]. Drogues Sante Soc 2008;7(2):9–45. doi: 10.7202/037564ar

10. Hathaway AD, Erickson PG. Drug reform principles and policy debates: harm reduction prospects for cannabis in Canada. J Drug Issues 2003;33(2):465–95. doi: 10.1177/002204260303300209

11. Swift W, Copeland J, Lenton S. Cannabis and harm reduction. Drug Alcohol Rev 2000;19(1):101–12. doi: 10.1080/09595230096200

12. Bagot KS, Kaminer Y. Harm reduction for youth in treatment for substance use disorders: one size does not fit all. Curr Addict Rep 2018;5(3):379–85. doi: 10.1007/s40429-018-0217-8

13. Jenkins EK, Slemon A, Haines-Saah RJ. Developing harm reduction in the context of youth substance use: insights from a multi-site qualitative analysis of young people's harm minimization strategies. Harm Reduct J 2017;14(1):53. doi: 10.1186/s12954-017-0180-z

For peer review only

14. Stockings E, Hall WD, Lynskey M, et al. Prevention, early intervention, harm reduction, and treatment of substance use in young people. Lancet Psychiatry 2016;3(3):280–96. doi: 10.1016/S2215-0366(16)00002-X

15. Buchanan J, Young L. The war on drugs – a war on drug users? Drugs (Abingdon Engl) 2000;7(4):409–22. doi: 10.1080/dep.7.4.409.422

16. Fallu J-S. Réduction des méfaits: histoire, concepts, pratiques, développements et enjeux [online]. Journée de la toxicomanie. Oral presentation, Journée de la toxicomanie, 23 Nov 2017; St-Liguori, Québec, Canada. https://www.reseautoxicomanie.com/wp-content/uploads/2017/12/Conférence-JS-Fallu_PNMVH-23-septembre-2017.pdf (accessed 13 Jul 2023).

17. Mancini MA, Linhorst DM, Broderick F, et al. Challenges to implementing the harm reduction approach. J Soc Work Pract Addict 2008;8(3):380–408. doi: 10.1080/15332560802224576

18. Karoll BR. Applying social work approaches, harm reduction, and practice wisdom to better serve those with alcohol and drug use disorders. J Soc Work 2010;10(3):263–81. doi: 10.1177/1468017310363635

19. Jenkins EK, Slemon A, Haines-Saah RJ. Developing harm reduction in the context of youth substance use: insights from a multi-site qualitative analysis of young people's harm minimization strategies. Harm Red J 2017;14(1):53. doi: 10.1186/s12954-017-0180-z

20. Fallu J-S, Brisson P. La réduction des méfaits liés à l'usage des drogues: historique, état des lieux, enjeux. In: Massé R, Mondou I, eds. Réduction des méfaits et tolérance en santé publique: enjeux éthiques et politique. Presses de l'Université Laval 2013:105–27.

21. Ritter A, Cameron J. A review of the efficacy and effectiveness of harm reduction strategies for alcohol, tobacco and illicit drugs. Drug Alcohol Rev 2006;25(6):611–24. doi: 10.1080/09595230600944529

22. Lau N, Sales P, Averill S, Murphy F, et al. A safer alternative: cannabis substitution as harm reduction. Drug Alcohol Rev 2015;34(6):654–9. doi: 10.1111/dar.12275

23. Tsemberis S, Gulcur L, Nakae M. Housing first, consumer choice, and harm reduction for homeless individuals with a dual diagnosis. Am J Public Health 2004;94(4):651–6. doi: 10.2105/AJPH.94.4.651

24. Poulin C, Nicholson J. Should harm minimization as an approach to adolescent substance use be embraced by junior and senior high schools?: empirical evidence from an integrated school- and community-based demonstration intervention addressing drug use among adolescents. Int J Drug Policy 2005;16(6):403–14. doi: 10.1016/j.drugpo.2005.11.001

25. McBride N, Farringdon F, Midford R, et al. Harm minimization in school drug education: Final results of the School Health and Alcohol Harm Reduction Project (SHAHRP). Addiction 2004;99(3):278–91. doi: 10.1111/j.1360-0443.2003.00620.x

26. Fischer B, Jones W, Shuper P, et al. 12-month follow-up of an exploratory 'brief intervention' for high-frequency cannabis users among Canadian university students. Subst Abuse Treat Prev Policy 2012;7(1):15. doi: 10.1186/1747-597X-7-15

For peer review only

27. Grossbard JR, Mastroleo NR, Kilmer JR, et al. Substance use patterns among first-year college students: secondary effects of a combined alcohol intervention. J Subst Abuse Treat 2010;39(4):384–90. doi: 10.1016/j.jsat.2010.07.001

28. Bertrand K, Blanchette-Martin N, Dufour M, et al. Projet IP-Jeunes : recenser et promouvoir les critères d'efficacité reconnus en intervention précoce auprès des jeunes présentant des comportements à risque quant à la consommation d'alcool et d'autres drogues, à la pratique des jeux de hasard et d'argent et à l'utilisation d'internet. Synthèse des résultats préliminaires, 2019-2020 [online]. IUD: Institut universitaire sur les dépendances; 2020. https://chairejeunesse.ca/documentation/projet-ip-jeunes-recenser-et-promouvoir-les-criteres-defficacite-reconnus-en-intervention-precoce-aupres-des-jeunes-presentant-des-comportements-a-risque-quant-a-la-consommation-da/ (accessed 13 Jul 2023).

29. Palfai TP, Saitz R, Winter M, et al. Web-based screening and brief intervention for student marijuana use in a university health center: pilot study to examine the implementation of eCHECKUP TO GO in different contexts. Addict Behav 2014;39(9):1346–52. doi: 10.1016/j.addbeh.2014.04.025

30. Sharp A, Barnett JT, Vroom EB. Community perceptions of harm reduction and its implications for syringe exchange policy. J Drug Issues 2020;50(4):507–23. doi: 10.1177/0022042620932289

31. Fallu J-S, Gilbert-Lambert MH, D'Arcy Dubois L, et al. Prévention de la consommation à risque ou problématique de substances psychoactives chez les jeunes hébergés en institution au Québec [online]. Centre intégré universitaire de santé et de services sociaux du Centre-Sud-de-l'Île-de-Montréal (Québec). Direction de l'enseignement universitaire et de la recherche. Institut universitaire sur les dépendances. 2022. https://iud.quebec/fr/etudes-et-recherche/programme-sur-lusage-et-les-dependances-aux-substances-puds/publications (accessed 13 Jul 2023).

32. MacCoun RJ. Toward a psychology of harm reduction. Am Psychol 1998;53(11):1199–1208. doi: 10.1037/0003-066X.53.11.1199

33. Mancini MA, Wyrick-Waugh W. Consumer and practitioner perceptions of the harm reduction approach in a community mental health setting. Community Ment Health J 2013;49(1):14–24. doi: 10.1007/s10597-011-9451-4

34. Koutroulis G. "That original tension". Negotiating abstinence in clinicians' accounts of harm reduction in nonresidential treatment of heroin withdrawal. J Subst Abuse Treat 2000;19(1):89–98. doi: 10.1016/S0740-5472(99)00087-2

35. Peters MDJ, Godfrey CM, Khalil H, et al. Guidance for conducting systematic scoping reviews. Int J Evid Based Healthc 2015;13(3):141–6. doi: 10.1097/XEB.0000000000000050

36. Tricco AC, Lillie E, Zarin W, et al. PRISMA extension for scoping reviews (PRISMA-ScR): checklist and explanation. Ann Intern Med 2018;169(7):467–73. doi: 10.7326/M18-0850

37. Levac D, Colquhoun H, O'Brien KK. Scoping studies: advancing the methodology. Implement Sci 2010;5(1):69. doi: 10.1186/1748-5908-5-69

38. Arksey H, O'Malley L. Scoping studies: towards a methodological framework. Int J Soc Res Methodol 2005;8(1):19–32. doi: 10.1080/1364557032000119616

For peer review only

39. Peters MDJ, Godfrey C, McInerney P, et al. Chapter 11: Scoping reviews. In: Aromataris E, Munn Z, eds. JBI Manual for Evidence Synthesis. JBI, 2020. doi: 10.46658/JBIMES-20-12

40. Peterson J, Pearce PF, Ferguson LA, et al. Understanding scoping reviews: definition, purpose, and process. J Am Assoc Nurse Pract 2017;29(1):12–6. doi: 10.1002/2327-6924.12380

41. Pollock D, Tricco AC, Peters MD, et al. Methodological quality, guidance, and tools in scoping reviews: a scoping review protocol. JBI Evid Synth 2022;20(4):1098–105. doi: 10.11124/JBIES-20-00570

42. McGowan J, Sampson M, Salzwedel DM, et al. PRESS peer review of electronic search strategies: 2015 guideline statement. J Clin Epidemiol 2016;75:40–6. doi: 10.1016/j.jclinepi.2016.01.021

43. Gedda M. Traduction française des lignes directrices PRISMA pour l'écriture et la lecture des revues systématiques et des méta-analyses [French translation of the PRISMA Reporting Guidelines for writing and reading systematic reviews and meta-analyses]. Kinesither Rev 2015;15(157):39–44. doi: 10.1016/j.kine.2014.11.004

44. Colquhoun HL, Levac D, O'Brien KK, et al. Scoping reviews: time for clarity in definition, methods, and reporting. J Clin Epidemiol 2014;67(12):1291–4. doi: 10.1016/j.jclinepi.2014.03.013

45. Hong QN, Pluye P, Fàbregues S, et al. Mixed methods appraisal tool (MMAT) version 2018 user guide. Registration of copyright (#1148552) [online]. Canadian Intellectual Property Office, Industry Canada; 2018. https://www.nccmt.ca/knowledge-repositories/search/232 (accessed 13 Jul 2023).

For peer review only

Appendix 1: Medline Search Strategy

Searched online: 10/10/2022

Search results: 251

#	Concept	Equations	Results
1	Harm reduction	Harm Reduction/	3898
2		Risk Reduction Behavior/	14132
3		(protective adj2 strateg*).ab,kf,ti.	2818
4		((reduc* or minimi*) adj5 (harm? or harmful or risk?)).ab,kf,ti.	239459
5		1 or 2 or 3 or 4	251821
6	Clinicians	exp Health Personnel/	588634
7		Social Workers/	971
8		Counselors/	541
9		exp Health Occupations/	1819039
10		exp Allied Health Occupations/	52626
11		exp Allied Health Personnel/	53169
12		(worker? or psychoeducator? or psycho-educator? or educator? or nurse? or criminologist? or psychologist? or clinician? or practitioner? or physician? or professional? or provider? or co?nselor or co?nselors or caregiver? or giver? or therapist? or psychotherapist? or staff? or personnel? or employee? or doctor?).ab,kf,ti.	2002544
13		6 or 7 or 8 or 9 or 10 or 11 or 12	3625104
14	Cannabis	Cannabis/	12307
15		"Marijuana Use"/	1689
16		Marijuana Abuse/	6905
17		Marijuana Smoking/	5425
18		(mari?uana or cannabis or hashish or Pot or weed or tetrahydrocannabinol or THC or CDB or cannabidiol or cannabinoid?).ab,kf,ti.	100005
19		14 or 15 or 16 or 17 or 18	102988
20	Strategies	("strateg*" or "approach*" or intervention? or prevent* or practice? or service? or "method*" or technique? or tactic? or co?nseling or treatment? or program? or "guide*").ab,kf,ti.	15074452
21	Combination of 3 concepts	5 and 13 and 19	272
22	Limit date	limit 21 to yr="1990 -Current"	272
23	Limit language	limit 22 to (english or french)	263
24	Filter OECD		
25	TOTAL	23 not 24	249
22	Combination of 4 concepts	5 and 13 and 19 and 20	251

For peer review only

Preferred Reporting Items for Systematic reviews and Meta-Analyses extension for Scoping Reviews (PRISMA-ScR) Checklist

SECTION	ITEM	PRISMA-ScR CHECKLIST ITEM	REPORTED ON PAGE #
**TITLE**			
Title	1	Identify the report as a scoping review.	1
**ABSTRACT**			
Structured summary	2	Provide a structured summary that includes (as applicable): background, objectives, eligibility criteria, sources of evidence, charting methods, results, and conclusions that relate to the review questions and objectives.	1
**INTRODUCTION**			
Rationale	3	Describe the rationale for the review in the context of what is already known. Explain why the review questions/objectives lend themselves to a scoping review approach.	3-6
Objectives	4	Provide an explicit statement of the questions and objectives being addressed with reference to their key elements (e.g., population or participants, concepts, and context) or other relevant key elements used to conceptualize the review questions and/or objectives.	6
**METHODS**			
Protocol and registration	5	Indicate whether a review protocol exists; state if and where it can be accessed (e.g., a Web address); and if available, provide registration information, including the registration number.	6-8
Eligibility criteria	6	Specify characteristics of the sources of evidence used as eligibility criteria (e.g., years considered, language, and publication status), and provide a rationale.	9-10
Information sources*	7	Describe all information sources in the search (e.g., databases with dates of coverage and contact with authors to identify additional sources), as well as the date the most recent search was executed.	8-9
Search	8	Present the full electronic search strategy for at least 1 database, including any limits used, such that it could be repeated.	8
Selection of sources of evidence†	9	State the process for selecting sources of evidence (i.e., screening and eligibility) included in the scoping review.	8
Data charting process‡	10	Describe the methods of charting data from the included sources of evidence (e.g., calibrated forms or forms that have been tested by the team before their use, and whether data charting was done independently or in duplicate) and any processes for obtaining and confirming data from investigators.	9
Data items	11	List and define all variables for which data were sought and any assumptions and simplifications made.	11-12
Critical appraisal of individual sources of evidence§	12	If done, provide a rationale for conducting a critical appraisal of included sources of evidence; describe the methods used and how this information was used in any data synthesis (if appropriate).	x
Synthesis of results	13	Describe the methods of handling and summarizing the data that were charted.	12

For peer review only - http://bmjopen.bmj.com/site/about/guidelines.xhtml

SECTION	ITEM	PRISMA-ScR CHECKLIST ITEM	REPORTED ON PAGE #
**RESULTS**			
Selection of sources of evidence	14	Give numbers of sources of evidence screened, assessed for eligibility, and included in the review, with reasons for exclusions at each stage, ideally using a flow diagram.	
Characteristics of sources of evidence	15	For each source of evidence, present characteristics for which data were charted and provide the citations.	
Critical appraisal within sources of evidence	16	If done, present data on critical appraisal of included sources of evidence (see item 12).	
Results of individual sources of evidence	17	For each included source of evidence, present the relevant data that were charted that relate to the review questions and objectives.	
Synthesis of results	18	Summarize and/or present the charting results as they relate to the review questions and objectives.	
**DISCUSSION**			
Summary of evidence	19	Summarize the main results (including an overview of concepts, themes, and types of evidence available), link to the review questions and objectives, and consider the relevance to key groups.	
Limitations	20	Discuss the limitations of the scoping review process.	
Conclusions	21	Provide a general interpretation of the results with respect to the review questions and objectives, as well as potential implications and/or next steps.	
**FUNDING**			
Funding	22	Describe sources of funding for the included sources of evidence, as well as sources of funding for the scoping review. Describe the role of the funders of the scoping review.	14

JBI = Joanna Briggs Institute; PRISMA-ScR = Preferred Reporting Items for Systematic reviews and Meta-Analyses extension for Scoping Reviews.
* Where *sources of evidence* (see second footnote) are compiled from, such as bibliographic databases, social media platforms, and Web sites.
† A more inclusive/heterogeneous term used to account for the different types of evidence or data sources (e.g., quantitative and/or qualitative research, expert opinion, and policy documents) that may be eligible in a scoping review as opposed to only studies. This is not to be confused with *information sources* (see first footnote).
‡ The frameworks by Arksey and O'Malley (6) and Levac and colleagues (7) and the JBI guidance (4, 5) refer to the process of data extraction in a scoping review as data charting.
§ The process of systematically examining research evidence to assess its validity, results, and relevance before using it to inform a decision. This term is used for items 12 and 19 instead of "risk of bias" (which is more applicable to systematic reviews of interventions) to include and acknowledge the various sources of evidence that may be used in a scoping review (e.g., quantitative and/or qualitative research, expert opinion, and policy document).

From: Tricco AC, Lillie E, Zarin W, O'Brien KK, Colquhoun H, Levac D, et al. PRISMA Extension for Scoping Reviews (PRISMAScR): Checklist and Explanation. Ann Intern Med. 2018;169:467–473. doi: 10.7326/M18-0850.

For peer review only
For peer review only - http://bmjopen.bmj.com/site/about/guidelines.xhtml

Facilitators of and barriers to healthcare providers' adoption of harm reduction in cannabis use: a scoping review protocol

Journal:	*BMJ Open*
Manuscript ID	bmjopen-2023-078427.R1
Article Type:	Protocol
Date Submitted by the Author:	28-Feb-2024

Complete List of Authors:	Haddad, Roula; Université de Montréal Faculté des Arts et des Sciences, Department of Psychology<br>Dagenais, Christian; Université de Montréal Faculté des Arts et des Sciences, Department of Psychology<br>Huynh, Christophe ; Institut Universitaire sur les Dépendances, Centre intégré universitaire de santé et de services sociaux du Centre-Sud-de-l'Île-de-Montréal ; Université de Montreal Département de Psychiatrie<br>Fallu, Jean-Sébastien; University of Montreal, Psychoeducation; Centre intégré universitaire de santé et de services sociaux du Centre-Sud-de-l'Île-de-Montréal du Québec, Centre de recherche en santé publique (CReSP)

**Primary Subject Heading**:	Addiction
Secondary Subject Heading:	Public health, Research methods
Keywords:	PUBLIC HEALTH, MENTAL HEALTH, Substance misuse < PSYCHIATRY, Health Services

SCHOLARONE™
Manuscripts

Facilitators of and barriers to healthcare providers' adoption of harm reduction in cannabis use: a scoping review protocol

Roula Haddad[1], Christian Dagenais[1], Christophe Huynh[2,3], Jean-Sébastien Fallu[2,4,5]

[1] Department of Psychology, Université de Montréal

[2] University Institute on Addictions, Centre intégré universitaire de santé et de services sociaux du Centre-Sud-de-l'Île-de-Montréal

[3] Department of Psychiatry and Addictology, Université de Montréal

[4] School of Psychoeducation, Université de Montréal

[5] Centre de recherche en santé publique (CReSP), Centre intégré universitaire de santé et de services sociaux du Centre-Sud-de-l'Île-de-Montréal

Correspondence to Roula Haddad; roula.haddad@umontreal.ca

ABSTRACT

Introduction. The high prevalence of cannabis use and the potential for negative effects indicate the need for effective prevention strategies and treatment of people who use cannabis (PWUC). Studies show that harm reduction (HR) in cannabis use is effective in minimizing the harmful consequences of the substance. However, health professionals often misunderstand it and resist its adoption due to various obstacles. To our knowledge, there has been no review of the scientific literature on the factors that facilitate or hinder practitioners' adoption of HR in cannabis use. To fill this gap, we aim to identify, through a scoping review, facilitators and barriers to healthcare providers' adoption of HR in cannabis use in Organisation for Economic Cooperation and Development (OECD) countries.

Methods and analysis. Our methodology will be guided by the six-step model initially proposed by Arksey and O'Malley (2005). The search strategy will be executed on different databases (Medline, PsycINFO, CINAHL, Web of Science, Embase, Sociological Abstracts, Érudit, BASE, Google Web, and Google Scholar) and will cover articles published between 1990 and October 2022. Empirical studies published in French or English in an OECD country and identifying factors

For peer review only

that facilitate or hinder healthcare providers' adoption of HR in cannabis use, will be included. Reference lists of the selected articles as well as relevant systematic reviews will be scanned to identify any missed publications by the electronic searches.

Ethics and dissemination. Ethics approval is not required. The results will be disseminated through various activities (e.g., publication in peer-reviewed journals, conferences, webinars, and knowledge translation activities). The results will also allow us to conduct a future study aiming to develop and implement a knowledge translation process among healthcare practitioners working with youth in Quebec in order to enhance their adoption of HR in cannabis use.

Strengths and limitations of this study

- The search strategy was co-developed by two information management specialists in the addiction and knowledge translation fields.
- The search strategy will aim to retrieve published articles on several health databases and unpublished studies found in the grey literature.
- Two reviewers will independently select the studies to be included in the scoping review throughout the entire study selection stage.
- The included studies will be limited to those published in French or English.
- The included studies will be limited to those published in OECD countries.

Key words: *harm reduction; cannabis; knowledge translation; healthcare*

For peer review only

INTRODUCTION

Cannabis use

Psychoactive substances (e.g., cannabis, alcohol, nicotine) are defined as substances whose use affects mental processes (e.g., perception, cognition, emotions, mood) and behaviors without necessarily leading to addiction (1). After tobacco and alcohol, cannabis represents the third most consumed psychoactive substance globally among adults and youth (2-5). Canada remains among the developed countries with the highest rates of cannabis use among young people and adults, with high prevalence in Quebec and multiple patterns of use (e.g., smoking, eating, vaporizing, vaping) (2, 5-9). In 2023, 26% of those 16 years of age and older reported using cannabis in the 12 months prior to the study, with a higher prevalence among those aged between 20 to 24 years old (10).

Cannabis use does not necessarily lead to dependence due to several factors such as infrequency in use, consumption of cannabis with low rates of δ-9-tetrahydrocannabinol (THC), availability, legality, and social acceptability (9, 11-15). The lifetime cumulative probability of transitioning from use to dependence was found to be the lowest for people who use cannabis (PWUC) (8.9%) compared to other substances such as nicotine (67.5%), alcohol (22.7%), and cocaine (20.9%) (15). Even with long-term exposure to cannabis, PWUC do not necessarily develop severe problems or a cannabis-dependence (5, 15, 16).

However, PWUC intensively or at-risk populations (i.e., pregnant persons, people presenting respiratory problems, a mental health comorbidity, having an early onset of continued tobacco use or concurrently smoking tobacco and cannabis) may develop a cannabis use disorder or experience harm at several levels (5, 12, 16-19). These harms may include a decreased academic or professional performance, a cognitive impairment, a deterioration of mental health (e.g., development of psychosis or depression), an increased occurrence of risky behaviors (e.g., cannabis-impaired driving), etc. (3-5, 12, 14, 18, 20-22). It is important to specify that a severe cannabis-induced mental health condition (e.g., psychosis) might occur among only 2% of PWUC (5). Despite the minimal probability of leading to potentially serious adverse consequences, the high prevalence of cannabis use as well as the potential harms that might be experienced, make it essential to implement effective intervention programs among PWUC (7, 12, 15, 17).

For peer review only

Abstinence approach

To address this reality, prevention and treatment programs based on the abstinence approach (i.e., total elimination of cannabis use), have been widely implemented (11, 13). The emergence of these programs has been also influenced by policies such as the "War on Drugs" (23). The abstinence-based model forms the basis of many programs developed to prevent or treat problematic cannabis use and has been applied with at-risk or marginalized populations (e.g., youth in the foster care system) (21, 24). Despite its potential to decrease the frequency or amount of substance use, the abstinence approach presents limited evidence to support its effectiveness and has been criticized for various reasons (21, 24-26). First, it does not provide PWUC with the necessary skills to identify and mitigate the harms associated with their use (21, 27). Second, the abstinence approach tends to focus more on the negative consequences of use through strategies that evoke fear, without necessarily taking into consideration the social context of cannabis use (20, 21). Third, the risks of relapse and dropout in these programs are also found to be high, leaving a significant number of PWUC for whom this goal remains unattainable (20, 28). Given these limitations of abstinence-oriented programs, other alternative and more flexible treatments, such as harm reduction (HR), are essential to reduce and mitigate cannabis-related harms (14, 21, 28, 29).

Harm reduction (HR)

Description

HR in cannabis use aims to minimize the harmful consequences of the substance at the individual, psychological, legal, and social levels among PWUC (19, 20, 28, 29). It offers a public health framework based on values of pragmatism and humanism, as it does not view substance use through a moral lens, but as an inevitable societal fact of long-standing (4, 11, 30, 31). Whether among adults or adolescents, elimination of substance use is unrealistic at the population level and should be the individual's choice without being imposed, as it represents an unwanted and impractical goal for some (e.g., in case of recreational or occasional use, in case of dual diagnosis combining psychiatric and substance use disorders) (27-29, 32, 33). HR in cannabis use also seeks to equip PWUC to make responsible and rational decisions and learn ways to reduce the negative consequences associated with their consumption (11, 12, 20, 27, 29, 31). To this end, HR clarifies the notion of safe substance use that is determined by the interaction of three components: the individual (height, weight, gender, physical and mental health status, state of mind, etc.); the drug

For peer review only

(quantity, frequency of use, tolerance to the product, combination with other products, quality, etc.); and the setting (location, time of day, interpersonal relationships, conflicts, laws, etc.) (4, 20, 27). In addition, HR takes into account the personal characteristics of PWUC (impulsivity, sensation-seeking, etc.) and addresses their potential ambivalence about stopping substance use, their feelings of failure upon relapse, their engagement in treatment, their social skills, their emotional regulation, etc. (20). In the "Lower Risk Cannabis Use Guidelines", Fischer (5) updated the initial recommendations to reduce the harms of cannabis use. These recommendations include delaying the initiation of cannabis use until late adolescence or the completion of puberty, consuming low-potency cannabis products, avoiding deep inhalation, using legal and quality-controlled cannabis products, etc. (5).

HR effectiveness

Interventions based on HR for non-injected drugs have been studied across various populations (e.g., youth, adults, people in housing programs presenting mental health conditions) and have shown promising results in decreasing the negative consequences associated with substance use (20, 28).

Several studies showed the effectiveness of HR strategies and current school-based HR programs (e.g., SHAHRP in the United Kingdom and SCIDUA in Canada) in developing safer attitudes toward substance use and in reducing negative consequences related to use (21, 22, 34, 35). The effectiveness of HR among youth who use cannabis has led the University Institute on Addictions (*Institut universitaire sur les dépendances*) in Quebec to recommend it as an intervention modality among this clientele (36). In addition, effective early interventions targeting college and university students with at-risk cannabis use are those that reduce the harms associated with cannabis use (17, 36-38). Moreover, Palfai (38) found that students participating in a web-based HR intervention (Marijuana eCHECKUP TO GO) showed statistically significant lower results in peer cannabis use after six months ($f^2 = .11$, [B = 7.45 (3.34), $p < .05$]).

HR in cannabis use was also found effective among adults (17, 39). After delivering a brief HR intervention for PWUC, Fischer (17) found significant reductions in risk outcome indicators only among the experimental group. At the 12th month follow-up, a change was maintained for "deep inhalation/breath-holding" (experimental group: Q = 13.1; $p < .05$; control group: Q = 4.8; $p > .05$), and "driving after cannabis use" (experimental group: Q = 9.3; $p < .05$; control group: Q = 0.9; $p >$

For peer review only

.05) (17). Furthermore, without completely abstaining from the substance, a functional improvement can be reached by treatment-seeking adults presenting a cannabis use disorder when they reduce the frequency and/or quantity of cannabis use (39). Reduction in the frequency of cannabis use was associated with a decrease in depression (F = 2.76, p = .04, n_p^2 =.04), anxiety (F = 3.70, p =.01, n_p^2 = .05), and cannabis-related problems (F = 8.95, p < .001, n_p^2 = .12) (39). In addition, a decrease in the quantity of cannabis consumption was associated with a decrease in anxiety (F = 3.02, p = .03, n_p^2 =.04) and cannabis-related problems (F = 3.24, p = .02, n_p^2 = .05) (39). A systematic review also highlighted that the adoption of HR strategies by PWUC acts as a protective factor for people with poor mental health, low self-regulation, high impulsivity, and high negative urgency (22).

Acceptability of harm reduction

Despite its proven effectiveness, the acceptability and applicability of HR by health and social services practitioners remain limited, and various factors facilitate or hinder its adoption (21, 40-42). MacCoun (43) showed that, among practitioners who did not adopt HR, some had based their decision on moral grounds, regardless of its effectiveness. Various barriers limit its use by practitioners. First, ambiguities in its conceptualization play an influential role; for example, some practitioners perceive HR as sending the wrong message, i.e., one of tolerating or even encouraging substance use (27, 28, 30, 44). Some do not perceive total cessation of substance use as a legitimate goal that could be achieved through HR (45). Also, there is often confusion between reducing use (frequency, quantity, etc.) and reducing harm (modifying consumption practices, such as contexts and mixtures, to reduce consequences) (30). These misconceptions point to the need for awareness-raising, training, and supervision of practitioners interested in this approach (28). Second, the adoption of HR can be hindered by ethical dilemmas, as well as by issues related to the personal and collective values of healthcare workers and the therapeutic model of abstinence (30). Indeed, it runs counter to traditional treatments by tolerating risky behaviors and accepting that HR in drug use is a legitimate outcome (30, 44). Practitioners may also fear the emergence of legal, social, and health problems among their clients (28). Third, its adoption may be limited by contextual barriers, such as lack of funding, stigma that undermines demand for care, resistance from local jurisdictions, and lack of services and trained personnel, particularly in the mental health sector (41). Healthcare providers' resistance to applying HR in cannabis use leads to limited

knowledge and utilization of effective HR techniques and guidelines among PWUC (5, 25). Among the study's participants (i.e., PWUC), Kruger (25) found that less than half of the participants believed that the listed HR techniques were effective and reported applying them. However, several factors seen as HR benefits have been found to facilitate its implementation by healthcare providers, such as: broadening the spectrum of acceptable goals, improving clients' decision-making skills, creating positive and quality relationships, and managing relapses (28). A study by Sharp (41) showed that clarifying the positive impacts of HR at the community level (e.g., safety) and ensuring the availability of resources could increase the likelihood of its adoption.

Purpose

Despite its proven effectiveness, several reasons might hinder HR adoption by health professionals among PWUC (30, 41). However, to date, there has been no review of the scientific literature that identifies the factors that facilitate or limit the adoption of HR in cannabis use. To fill this gap, we aim to identify, through a scoping review, facilitators and barriers to healthcare providers' adoption of HR in cannabis use.

METHODS AND ANALYSIS

This study will follow the methodological steps of scoping reviews (46). This type of review has become more prevalent in recent years and is a type of knowledge synthesis review (46, 47). There is no universal definition for scoping reviews; however, a variety of factors distinguish them from other types of knowledge synthesis (48). First, scoping reviews address broad research questions and include studies with different designs and multiple sources of evidence to provide an overview of the available knowledge around a concept (49). On the contrary, a systematic review following Cochrane standards explores more specific research questions based on detailed inclusion and exclusion criteria (50-52). Second, while assessment of the methodological quality of included studies is recommended for scoping reviews, it is not mandatory, whereas assessment of the risk of bias of included studies is required for Cochrane-type systematic reviews (50, 52). Researchers undertake scoping reviews for a variety of reasons: 1) to review research activity in a given area; 2) to determine the feasibility and appropriateness of conducting a systematic review based on Cochrane standards; 3) to summarize and disseminate the results of existing research on a topic; and/or 4) to identify a gap in the literature and draw conclusions regarding a topic (49). Our

For peer review only

methodological choice is underpinned by three of these reasons: once the research activity around the topic has been consulted, the findings will be summarized and used to support a second study aimed at disseminating knowledge to practitioners through a knowledge translation process. This will also allow us to identify gaps in the literature and draw conclusions related to the topic.

Arksey and O'Malley (49) were the first to propose a six-step model for conducting scoping reviews. Our methodology will be guided by this model, which was later refined by Levac (48) and revised by members of the Joanna Briggs Institute (50). The six stages we will follow are:

- Stage 1: Determining the research question and the objective
- Stage 2: Identifying relevant studies
- Stage 3: Selecting studies
- Stage 4: Charting the data
- Stage 5: Collating, summarizing, and reporting the results
- Stage 6: Conducting a consultation exercise

The research protocol will be reported using the Preferred Reporting Items for Systematic Reviews and Meta-Analyses Extension for Scoping Reviews (PRISMA-ScR) grid (47). This grid is an extension of the PRISMA grid originally developed for Cochrane-type systematic reviews and helps to ensure the transparency and reproducibility of the study (47, 50).

Stage 1: Determining the research question and the objective

Arksey and O'Malley's (49) model suggests that scoping reviews should begin not only with identifying research questions but also with clarifying the resulting objectives (48, 50). Our scoping review is exploratory and aims to identify facilitators and barriers to healthcare providers' adoption of HR in cannabis use in OECD countries. Based on the Population–Concept–Context (PCC) model, which allows the broad scope of the study to be respected without specifying restrictive inclusion criteria (50), we formulated the research question: What factors influence providers (population) in the healthcare field (context) to adopt HR in cannabis use (concept)? Specific research questions associated with the components of the PCC model were also identified.

- Question 1, related to the concept and context components: What are the facilitators and barriers to healthcare providers' adoption of HR in cannabis use?
- Question 2, related to the population component: Who is the clientele of the providers identified in the studies?

For peer review only

- Question 3, related to the concept component: What is the definition of HR in cannabis use?

Stage 2: Identifying relevant studies

Search strategy

The search strategy was developed through an iterative process. A senior librarian at the Quebec Addiction Library (*Bibliothèque québécoise sur les dépendances*) first developed three search strategies with different concepts and ran them on the Medline database. The first 50 results of each strategy were consulted, which led us to opt the one that grouped key terms related to the following concepts: harm reduction, clinicians, and cannabis (see Supplemental Appendix 1). The search strategy was then reviewed by a second information professional working in the RENARD Team for Knowledge Translation who, in turn, adapted it to the selected databases. The final search strategy executed on all the databases was then validated by the RENARD Team information specialist. The Peer Review of Electronic Search Strategies (PRESS) tool served as a guide for the librarians in this process (53). The search strategy executed on each database is presented in Supplemental Appendix 1. All search strategies were executed on October 10th, 2022.

Information sources

To identify relevant published and unpublished studies for inclusion in the study, various sources of information will be reviewed (46, 49, 50). With the guidance of the two librarians, the search strategy will be executed on the leading health and intervention databases: Medline, PsycINFO, Cumulative Index to Nursing and Allied Health Literature (CINAHL), Web of Science, Embase, and Sociological Abstracts. To explore the grey literature (e.g., theses, research reports, etc.), the search strategy will be adapted to the Google Web and Google Scholar search engines, as well as the Érudit (French database) and BASE databases. All documents identified will be entered into Zotero software for the research team members to access. To identify any missed publication by the electronic searches, reference lists of selected articles will be manually searched and relevant systematic reviews will be scanned to identify their included studies.

For peer review only

Stage 3: Study selection

After running the search strategy on the selected databases and completing the second stage, all identified duplicates will be removed. The remaining documents will then be entered into Covidence software. Two reviewers will independently select relevant documents for inclusion by reading the titles and abstracts of the identified studies. Their decisions will be based on the specified inclusion and exclusion criteria. The inter-rater agreement between the reviewers will first be calculated and then they will meet regularly to resolve selection conflicts and refine the eligibility criteria as needed. After this first step, the documents selected and deemed potentially relevant will then be the subject of the second step, the full-text reading. Again, the two reviewers will independently record their choices on Covidence. The inter-rater agreement for this step will be calculated and the reviewers will then resolve any new conflicts. A third reviewer will be called upon as needed for any conflict resolution. This will complete the document selection stage, whose steps will be presented in a PRISMA diagram (54).

Inclusion and exclusion criteria

Inclusion and exclusion criteria have been specified and will be fine-tuned as needed to select relevant studies (Table 1). Using this process, empirical studies of quantitative, qualitative, or mixed designs, identifying factors that facilitate or hinder healthcare providers' adoption of HR in cannabis use, will be selected. To facilitate comparison and generalization of the results to the Quebec context, the review will be limited to studies conducted in any of the 38 OECD countries. Articles published between 1990 and October 2022 will be included, as it was early in that decade that HR gained international prominence and its scope of application began to expand. Papers not meeting these inclusion criteria will be excluded. Systematic reviews will be excluded to avoid duplication and ensure equal representation of the selected papers; the executed search strategy might have already captured studies included in a potentially relevant systematic review. However, systematic reviews' reference lists will be examined to identify additional relevant studies that might be selected.

For peer review only

Table 1. Inclusion and exclusion criteria

Criteria	Inclusion criteria	Exclusion criteria
**Type of study**	- Empirical study: quantitative, qualitative, or mixed	- Study that does not present empirical results (e.g., theoretical study, conceptual framework, etc.) or knowledge review (e.g., systematic or literature review)<br>- Interviews
**Type of documents**	- Peer-reviewed scientific articles, research reports, dissertations, theses	- Books and practice guides
**Conceptual framework**	- Harm reduction (HR) in cannabis use<br>- Cannabis risk reduction<br>- Non-abstinence in cannabis use	- Another conceptual framework
**Objective**	- Identification of factors[1] facilitating or hindering practitioners' adoption of HR[2] in cannabis use	- Evaluation of the effectiveness of interventions based on HR<br>OR<br>- Stakeholder perceptions of the use of cannabis as an HR strategy to circumvent the effects of other drugs<br>OR<br>- Attitudes toward decriminalization of cannabis
**Psychoactive substance being studied**	- Marijuana, hashish, or cannabis for non-medical purposes<br>- "Drug" if cannabis is part of its conceptualization in the study	- Any substance other than marijuana, hashish, or non-medical cannabis (e.g., tobacco, alcohol, medical cannabis, MDMA, Ecstasy)<br>- Study that focuses on "performance and image enhancing drugs" or "crack" or "new psychoactive substances"
**Target population**	- Practitioners[3] working in the health field<br>- Practitioners in training	- PWUC[4]
**Country of study**	- OECD countries	- Other countries
**Publication date**	- From 1990 onwards	- Before 1990
**Language**	- French and/or English	- Languages other than French or English or text not available

[1] "Factors" include perceptions, beliefs, facilitators, obstacles, oppositions, attitudes, opinions, barriers, biases, motivations, preferences, determinants, incentives, influences, and perspectives on the adoption of HR in cannabis use, as well as its acceptability and receptivity.

[2] *"Approach" refers to strategies, interventions, practices, services, methods, techniques, treatments, programs, or guides for the HR approach in cannabis use.*

[3] *"Practitioners" include healthcare personnel, professionals, or practitioners, allied healthcare personnel, professionals, or practitioners, social workers, counselors, psychoeducators, educators, nurses, criminologists, psychologists, clinicians, caregivers, therapists, psychotherapists, and physicians.*

[4] *Studies addressing the views of people who use cannabis (PWUC) regarding HR or its adoption by practitioners will be excluded.*

Stage 4: Charting the data

To analyze the selected studies on a common basis, specific variables of interest will be identified based on the research questions (49). These will form the components of summary sheets that will be developed in Microsoft Excel and used to extract results (Table 2). This method is an analytical descriptive recording of the data (49, 50, 55). The first author (RH) will extract the data from the included studies and create the summary sheets. The research supervisor (CD) will validate the summary sheets throughout the process and ensure their alignment with the research questions (50).

Table 2. Summary sheets

General variables	Specific variables
**General characteristics of the study**	Study title<br>Author(s)<br>Language of publication<br>Date of publication<br>Period of publication<br>Journal<br>Type of article<br>Full reference<br>Country of study<br>Psychoactive substance under study<br>The legal status of cannabis in the country of study
**Introduction**	Main concepts<br>Definition of the main concept: HR in cannabis use<br>Research question(s)<br>Objective(s)<br>Hypothesis
**Methodology**	Study design

For peer review only - http://bmjopen.bmj.com/site/about/guidelines.xhtml

	Target population<br>Place of work of the target population<br>Inclusion criteria of participants<br>Recruitment method<br>Sample size<br>Country of origin of participants<br>The clientele of the population recruited<br>Data collection method<br>Analysis steps
**Results**	Sample presentation<br>Key findings: 1) facilitators and 2) barriers to practitioners' adoption of HR in cannabis use<br>Secondary outcomes or other results
**Conclusion**	Study strengths<br>Study limitations<br>Gaps in the literature and future research needs

Stage 5: Collating, summarizing, and reporting the results

Based on the eligibility criteria, studies deemed relevant will be collected, summarized, and reported. They will be subjected to 1) a numerical analysis, and 2) a narrative organization encompassing a descriptive qualitative analysis (47, 49, 50). A numerical analysis of the scope, nature, and distribution of the included studies will be performed to various characteristics: date of publication, country of origin of the studies, and type of document. Subsequently, a narrative organization of the results will be produced to identify the relationships between the data and the research questions. The summary sheets will be combined, tabulated, and synthesized, and will then be subjected to a descriptive qualitative analysis (Table 3).

Table 3. Narrative organization of the included studies

**Data**	**Study 1**	**Study 2**	**Study …**
Type of publication			
Date of publication			
Country of study			
Legal status of cannabis in the country of the study			
Definition of HR to cannabis use			
Design of the study			

For peer review only

Target population
Place of work of the target population
The clientele of the target population
Data collection method
Key findings:<br>- Facilitators or enabling conditions<br>- Barriers or adverse conditions
Secondary outcomes

Stage 6: Consultation exercise

Expert consultation is an optional step that promotes methodological rigor in scoping reviews (49). In this study, the project's supervisor and co-researchers will be solicited as consultants. Members of the RENARD team and researchers involved in the field of substance use and harm reduction will be consulted to help clarify findings and validate the resulting recommendations (49). Consultations will be conducted: 1) after preliminary results have been obtained, and 2) after analyses of the results have been completed.

Patient and public involvement

None.

ETHICS AND DISSEMINATION

To our knowledge, this is the first scoping review on factors that facilitate or hinder healthcare providers' adoption of HR in cannabis use. Other reviews have studied HR interventions in general among practitioners working with a specific population. This study will provide a clear picture of the factors at play when adopting HR, and the results could potentially be generalizable to OECD countries. The present study is exempt from ethics approval because it involves no patient or personal data collection. The results are expected to be ready by March 2024. They will be disseminated, alongside the scoping review protocol, through various activities (e.g., publication in peer-reviewed journals, conferences, webinars, posters, *Three Minute Thesis* competition).

After completing the scoping review, we will be able to conduct a future study aiming to implement a knowledge translation plan among practitioners working with youth in Quebec to enhance and expand their adoption of HR in cannabis use.

For peer review only

Acknowledgments. We wish to thank Karine Bélanger, senior librarian at the *Bibliothèque québécoise sur les dépendances* in Quebec for developing the initial search strategy used in this scoping review. We also wish to thank Julie Desnoyers, the information specialist affiliated with the RENARD Team for Knowledge Translation at Université de Montréal, for validating the search strategy and adapting it to the selected databases. Finally, we want to thank Donna Riley for editing the manuscript.

Contributors. RH, CD, CH, and JSF conceptualized the study. RH drafted the protocol. CD, CH, and JSF critically revised the manuscript. RH wrote the final draft manuscript and all the authors approved it.

Funding. This work was supported by the *Ministère de la Santé et des Services sociaux* (MSSS), *Fonds de recherche du Québec – Santé* (FRQS), and *Fonds de recherche du Québec – Société et culture* (FRQSC) (Grant number: 2023-0PTR-322652). A grant obtained by the *Centre de recherche en santé publique* (CReSP) helped in partially covering the revision and publication fees of this scoping review protocol.

Competing interests. None declared.

For peer review only

FULL REFERENCES

1. World Health Organization. Drugs (psychoactive) [online]; 2022. https://www.who.int/health-topics/drugs-psychoactive#tab=tab_1 (accessed 13 Jul 2023).

2. Adinoff B, Cooper ZD. Cannabis legalization: progress in harm reduction approaches for substance use and misuse. Am J Drug Alcohol Abuse 2019;45(6):707–12. doi: 10.1080/00952990.2019.1680683.

3. Jiloha RC. Prevention, early intervention, and harm reduction of substance use in adolescents. Indian J Psychiatry 2017;59(1):111–8. doi: 10.4103%2F0019-5545.204444.

4. Melamede R. Harm reduction-the cannabis paradox. Harm Reduct J 2005;2(1):17. doi: 10.1186/1477-7517-2-17.

5. Fischer B, Robinson T, Bullen C, Curran V, Jutras-Aswad D, Medina-Mora ME, et al. Lower-Risk Cannabis Use Guidelines (LRCUG) for reducing health harms from non-medical cannabis use: A comprehensive evidence and recommendations update. Int J Drug Policy. 2022;99:103381.

6. Poulin C, Elliott D. Alcohol, tobacco and cannabis use among Nova Scotia adolescents: implications for prevention and harm reduction. CMAJ 1997;156(10):1387–93. PMID: **9164396**.

7. Gouvernement du Québec. Plan d'action interministériel en dépendances 2018-2028 – Prévenir, réduire et traiter les conséquences associées à la consommation de substances psychoactives, à la pratique de jeux de hasard et d'argent et à l'utilisation d'Internet [online]. 2018. https://publications.msss.gouv.qc.ca/msss/document-002078/ (accessed 13 Jul 2023).

8. Zuckermann AM, Gohari MR, Romano I, et al. Changes in cannabis use modes among Canadian youth across recreational cannabis legalization: data from the COMPASS prospective cohort study. Addict Behav 2021;122:107025. doi: 10.1016/j.addbeh.2021.107025.

9. Fischer B, Russell C, Sabioni P, Van Den Brink W, Le Foll B, Hall W, et al. Lower-risk cannabis use guidelines: a comprehensive update of evidence and recommendations. Am J Public Health. 2017;107(8):e1-e12.

For peer review only

10. Government of Canada. Canadian Cannabis Survey 2023: Summary [online]. 2023. https://www.canada.ca/fr/sante-canada/services/drogues-medicaments/cannabis/recherches-donnees/enquete-canadienne-cannabis-2023-sommaire.html#s2-2 (accessed 13 Feb 2024).

11. Hathaway AD, Erickson PG. Drug reform principles and policy debates: harm reduction prospects for cannabis in Canada. J Drug Issues 2003;33(2):465–95. doi: 10.1177/002204260303300209.

12. Swift W, Copeland J, Lenton S. Cannabis and harm reduction. Drug Alcohol Rev 2000;19(1):101–12. doi: 10.1080/09595230096200.

13. Stockings E, Hall WD, Lynskey M, et al. Prevention, early intervention, harm reduction, and treatment of substance use in young people. Lancet Psychiatry 2016;3(3):280–96. doi: 10.1016/S2215-0366(16)00002-X.

14. Englund A, Freeman TP, Murray RM, McGuire P. Can we make cannabis safer? Lancet Psychiatry. 2017;4(8):643-8. doi: 10.1016/S2215-0366(17)30075-5.

15. Lopez-Quintero C, de los Cobos JP, Hasin DS, Okuda M, Wang S, Grant BF, et al. Probability and predictors of transition from first use to dependence on nicotine, alcohol, cannabis, and cocaine: Results of the National Epidemiologic Survey on Alcohol and Related Conditions (NESARC). Drug Alcohol Depend. 2011;115(1-2):120-30. doi: 10.1016/j.drugalcdep.2010.11.004.

16. Hindocha C, Shaban ND, Freeman TP, Das RK, Gale G, Schafer G, et al. Associations between cigarette smoking and cannabis dependence: a longitudinal study of young cannabis users in the United Kingdom. Drug Alcohol Depend. 2015;148:165-71. doi: 10.1016/j.drugalcdep.2015.01.004.

17. Fischer B, Jones W, Shuper P, et al. 12-month follow-up of an exploratory 'brief intervention' for high-frequency cannabis users among Canadian university students. Subst Abuse Treat Prev Policy 2012;7(1):15. doi: 10.1186/1747-597X-7-15.

18. Lemyre A, Poliakova N, Bélanger RE. The relationship between tobacco and cannabis use: a review. Subst Use Misuse. 2019;54(1):130-45. doi: 10.1080/10826084.2018.1512623.

For peer review only

19. Winer JM, Yule AM, Hadland SE, Bagley SM. Addressing adolescent substance use with a public health prevention framework: the case for harm reduction. Ann Med. 2022;54(1):2123-36. doi: 10.1080/07853890.2022.2104922.

20. Bagot KS, Kaminer Y. Harm reduction for youth in treatment for substance use disorders: one size does not fit all. Curr Addict Rep 2018;5(3):379–85. doi: 10.1007/s40429-018-0217-8.

21. Jenkins EK, Slemon A, Haines-Saah RJ. Developing harm reduction in the context of youth substance use: insights from a multi-site qualitative analysis of young people's harm minimization strategies. Harm Reduct J 2017;14(1):53. doi: 10.1186/s12954-017-0180-z.

22. González-Ponce BM, Rojas-Tejada AJ, Carmona-Márquez J, Lozano-Rojas ÓM, Díaz-Batanero C, Fernández-Calderón F. Harm reduction strategies among university students who use alcohol and cannabis, and related psychological variables: a systematic review. J Psychoactive Drugs. 2022;54(5):403-18. doi: 10.1080/02791072.2021.2023240.

23. Buchanan J, Young L. The war on drugs – a war on drug users? Drugs (Abingdon Engl) 2000;7(4):409–22. doi: 10.1080/dep.7.4.409.422.

24. Howe EJ, Bishop LD, Torraville BS, Rowe EC, Kinzel E, Donnan JR. Canadian cannabis education resources to support youth health literacy: A scoping review and environmental scan. Health Educ J. 2023;82(7):766-78. doi: 10.1177/00178969231191085.

25. Kruger JS, Kruger D, Collins RL. Knowledge and practice of harm reduction strategies among people who report frequent cannabis use. Health Promot Pract. 2021;22(1):24-30. doi: 10.1177/1524839920923999.

26. Watson TM, Valleriani J, Hyshka E, Rueda S. Cannabis legalization in the provinces and territories: missing opportunities to effectively educate youth? Can J Public Health. 2019;110:472-5. doi: 10.17269/s41997-019-00209-0.

27. Fallu J-S. Réduction des méfaits: histoire, concepts, pratiques, développements et enjeux [online]. Journée de la toxicomanie. Oral presentation, Journée de la toxicomanie, 23 Nov 2017; St-Liguori, Québec, Canada. https://www.reseautoxicomanie.com/wp-content/uploads/2017/12/Conférence-JS-Fallu_PNMVH-23-septembre-2017.pdf (accessed 13 Jul 2023).

For peer review only

28. Mancini MA, Linhorst DM, Broderick F, et al. Challenges to implementing the harm reduction approach. J Soc Work Pract Addict 2008;8(3):380–408. doi: 10.1080/15332560802224576.

29. Karoll BR. Applying social work approaches, harm reduction, and practice wisdom to better serve those with alcohol and drug use disorders. J Soc Work 2010;10(3):263–81. doi: 10.1177/1468017310363635.

30. Fallu J-S, Brisson P. La réduction des méfaits liés à l'usage des drogues: historique, état des lieux, enjeux. In: Massé R, Mondou I, eds. Réduction des méfaits et tolérance en santé publique: enjeux éthiques et politique. Presses de l'Université Laval 2013:105–27.

31. Ritter A, Cameron J. A review of the efficacy and effectiveness of harm reduction strategies for alcohol, tobacco and illicit drugs. Drug Alcohol Rev 2006;25(6):611–24. doi: 10.1080/09595230600944529.

32. Lau N, Sales P, Averill S, Murphy F, et al. A safer alternative: cannabis substitution as harm reduction. Drug Alcohol Rev 2015;34(6):654–9. doi: 10.1111/dar.12275.

33. Tsemberis S, Gulcur L, Nakae M. Housing first, consumer choice, and harm reduction for homeless individuals with a dual diagnosis. Am J Public Health 2004;94(4):651–6. doi: 10.2105/AJPH.94.4.651.

34. McBride N, Farringdon F, Midford R, et al. Harm minimization in school drug education: Final results of the School Health and Alcohol Harm Reduction Project (SHAHRP). Addiction 2004;99(3):278–91. doi: 10.1111/j.1360-0443.2003.00620.x.

35. Poulin C, Nicholson J. Should harm minimization as an approach to adolescent substance use be embraced by junior and senior high schools?: empirical evidence from an integrated school- and community-based demonstration intervention addressing drug use among adolescents. Int J Drug Policy 2005;16(6):403–14. doi: 10.1016/j.drugpo.2005.11.001.

36. Bertrand K, Blanchette-Martin N, Dufour M, et al. Projet IP-Jeunes : recenser et promouvoir les critères d'efficacité reconnus en intervention précoce auprès des jeunes présentant des comportements à risque quant à la consommation d'alcool et d'autres drogues, à la pratique des jeux de hasard et d'argent et à l'utilisation d'internet. Synthèse des résultats préliminaires, 2019-2020 [online]. IUD: Institut universitaire sur les dépendances; 2020.

For peer review only

https://chairejeunesse.ca/documentation/projet-ip-jeunes-recenser-et-promouvoir-les-criteres-defficacite-reconnus-en-intervention-precoce-aupres-des-jeunes-presentant-des-comportements-a-risque-quant-a-la-consommation-da/ (accessed 13 Jul 2023).

37. Grossbard JR, Mastroleo NR, Kilmer JR, et al. Substance use patterns among first-year college students: secondary effects of a combined alcohol intervention. J Subst Abuse Treat 2010;39(4):384–90. doi: 10.1016/j.jsat.2010.07.001.

38. Palfai TP, Saitz R, Winter M, et al. Web-based screening and brief intervention for student marijuana use in a university health center: pilot study to examine the implementation of eCHECKUP TO GO in different contexts. Addict Behav 2014;39(9):1346–52. doi: 10.1016/j.addbeh.2014.04.025.

39. Sherman BJ, Sofis MJ, Borodovsky JT, Gray KM, McRae-Clark AL, Budney AJ. Evaluating cannabis use risk reduction as an alternative clinical outcome for cannabis use disorder. Psychol Addict Behav. 2022;36(5):505. doi: 10.1037/adb0000760.

40. Fallu J-S, Gilbert-Lambert MH, D'Arcy Dubois L, et al. Prévention de la consommation à risque ou problématique de substances psychoactives chez les jeunes hébergés en institution au Québec [online]. Centre intégré universitaire de santé et de services sociaux du Centre-Sud-de-l'Île-de-Montréal (Québec). Direction de l'enseignement universitaire et de la recherche. Institut universitaire sur les dépendances. 2022. https://iud.quebec/fr/etudes-et-recherche/programme-sur-lusage-et-les-dependances-aux-substances-puds/publications (accessed 13 Jul 2023).

41. Sharp A, Barnett JT, Vroom EB. Community perceptions of harm reduction and its implications for syringe exchange policy. J Drug Issues 2020;50(4):507–23. doi: 10.1177/0022042620932289.

42. Lindenfeld Z, Franz B, Cronin C, Chang JE. Hospital adoption of harm reduction and risk education strategies to address substance use disorders. Am J Drug Alcohol Abuse. 2023;49(2):206-15. doi: 10.1080/00952990.2023.2169832.

43. MacCoun RJ. Toward a psychology of harm reduction. Am Psychol 1998;53(11):1199–1208. doi: 10.1037/0003-066X.53.11.1199.

For peer review only

44. Mancini MA, Wyrick-Waugh W. Consumer and practitioner perceptions of the harm reduction approach in a community mental health setting. Community Ment Health J 2013;49(1):14–24. doi: 10.1007/s10597-011-9451-4.

45. Koutroulis G. "That original tension". Negotiating abstinence in clinicians' accounts of harm reduction in nonresidential treatment of heroin withdrawal. J Subst Abuse Treat 2000;19(1):89–98. doi: 10.1016/S0740-5472(99)00087-2.

46. Peters MDJ, Godfrey CM, Khalil H, et al. Guidance for conducting systematic scoping reviews. Int J Evid Based Healthc 2015;13(3):141–6. doi: 10.1097/XEB.0000000000000050.

47. Tricco AC, Lillie E, Zarin W, et al. PRISMA extension for scoping reviews (PRISMA-ScR): checklist and explanation. Ann Intern Med 2018;169(7):467–73. doi: 10.7326/M18-0850.

48. Levac D, Colquhoun H, O'Brien KK. Scoping studies: advancing the methodology. Implement Sci 2010;5(1):69. doi: 10.1186/1748-5908-5-69.

49. Arksey H, O'Malley L. Scoping studies: towards a methodological framework. Int J Soc Res Methodol 2005;8(1):19–32. doi: 10.1080/1364557032000119616.

50. Peters MDJ, Godfrey C, McInerney P, et al. Chapter 11: Scoping reviews. In: Aromataris E, Munn Z, eds. JBI Manual for Evidence Synthesis. JBI, 2020. doi: 10.46658/JBIMES-20-12.

51. Peterson J, Pearce PF, Ferguson LA, et al. Understanding scoping reviews: definition, purpose, and process. J Am Assoc Nurse Pract 2017;29(1):12–6. doi: 10.1002/2327-6924.12380.

52. Pollock D, Tricco AC, Peters MD, et al. Methodological quality, guidance, and tools in scoping reviews: a scoping review protocol. JBI Evid Synth 2022;20(4):1098–105. doi: 10.11124/JBIES-20-00570.

53. McGowan J, Sampson M, Salzwedel DM, et al. PRESS peer review of electronic search strategies: 2015 guideline statement. J Clin Epidemiol 2016;75:40–6. doi: 10.1016/j.jclinepi.2016.01.021.

54. Gedda M. Traduction française des lignes directrices PRISMA pour l'écriture et la lecture des revues systématiques et des méta-analyses [French translation of the PRISMA Reporting

For peer review only

Guidelines for writing and reading systematic reviews and meta-analyses]. Kinesither Rev 2015;15(157):39–44. doi: 10.1016/j.kine.2014.11.004.

55. Colquhoun HL, Levac D, O'Brien KK, et al. Scoping reviews: time for clarity in definition, methods, and reporting. J Clin Epidemiol 2014;67(12):1291–4. doi: 10.1016/j.jclinepi.2014.03.013.

For peer review only

Facilitators of and barriers to healthcare providers' adoption of harm reduction in cannabis use: a scoping review protocol

Roula Haddad, Christian Dagenais, Christophe Huynh, Jean-Sébastien Fallu

Supplemental Appendix 1: Search strategies executed on all databases

Appendix 1: Medline Search Strategy

#	Concept	Equations	Results
1	Harm reduction	Harm Reduction/	3898
2		Risk Reduction Behavior/	14132
3		(protective adj2 strateg*).ab,kf,ti.	2818
4		((reduc* or minimi*) adj5 (harm? or harmful or risk?)).ab,kf,ti.	239459
5		1 or 2 or 3 or 4	251821
6	Clinicians	exp Health Personnel/	588634
7		Social Workers/	971
8		Counselors/	541
9		exp Health Occupations/	1819039
10		exp Allied Health Occupations/	52626
11		exp Allied Health Personnel/	53169
12		(worker? or psychoeducator? or psycho-educator? or educator? or nurse? or criminologist? or psychologist? or clinician? or practitioner? or physician? or professional? or provider? or co?nselor or co?nselors or caregiver? or giver? or therapist? or psychotherapist? or staff? or personnel? or employee? or doctor?).ab,kf,ti.	2002544
13		6 or 7 or 8 or 9 or 10 or 11 or 12	3625104
14	Cannabis	Cannabis/	12307
15		"Marijuana Use"/	1689
16		Marijuana Abuse/	6905
17		Marijuana Smoking/	5425
18		(mari?uana or cannabis or hashish or Pot or weed or tetrahydrocannabinol or THC or CDB or cannabidiol or cannabinoid?).ab,kf,ti.	100005
19		14 or 15 or 16 or 17 or 18	102988
20	Strategies	("strateg*" or "approach*" or intervention? or prevent* or practice? or service? or "method*" or technique? or tactic? or co?nseling or treatment? or program? or "guide*").ab,kf,ti.	15074452
21	Combination of 3 concepts	5 and 13 and 19	272
22	Limit date	limit 21 to yr="1990 -Current"	272
23	Limit language	limit 22 to (english or french)	263
24	Filter OECD		
25	TOTAL	23 not 24	249
22	Combination of 4 concepts	5 and 13 and 19 and 20	251

For peer review only

Appendix 2: PsycINFO

(((((((title: (reduc*))) OR ((title: (minimi*)))) NEAR/5 (((title: (harm))) OR ((title: (harms))) OR ((title: (harmful))) OR ((title: (risk))) OR ((title: (risks)))) OR (((abstract: (reduc*))) OR ((abstract: (minimi*)))) NEAR/5 (((abstract: (harm))) OR ((abstract: (harms))) OR ((abstract: (harmful))) OR ((abstract: (risk))) OR ((abstract: (risks)))) OR (((Keywords: (reduc*))) OR ((Keywords: (minimi*)))) NEAR/5 (((Keywords: (harm))) OR ((Keywords: (harms))) OR ((Keywords: (harmful))) OR ((Keywords: (risk))) OR ((Keywords: (risks))))) OR ((((title: (protective NEAR/2 strateg*)))) OR (((abstract: (protective NEAR/2 strateg*)))) OR (((Keywords: (protective NEAR/2 strateg*))))) OR ((((MeSH: (Risk Reduction Behavior))))) OR ((((MeSH: (Harm Reduction)))))) AND (((((MeSH: (Health Personnel))))) OR ((((MeSH: (Social Workers))))) OR ((((MeSH: (Counselors))))) OR ((((MeSH: (Health Occupations))))) OR ((((MeSH: (Allied Health Occupations))))) OR ((((MeSH: (Allied Health Personnel))))) OR ((((Keywords: (worker*))) OR ((Keywords: (psychoeducator*))) OR ((Keywords: (psycho-educator*))) OR ((Keywords: (educator*))) OR ((Keywords: (nurse*))) OR ((Keywords: (criminologist*))) OR ((Keywords: (psychologist*))) OR ((Keywords: (clinician*))) OR ((Keywords: (practitioner*))) OR ((Keywords: (physician*))) OR ((Keywords: (professional*))) OR ((Keywords: (provider*))) OR ((Keywords: (conselor))) OR ((Keywords: (conselors))) OR ((Keywords: (counselor))) OR ((Keywords: (counselers))) OR ((Keywords: (caregiver*))) OR ((Keywords: (giver*))) OR ((Keywords: (therapist*))) OR ((Keywords: (psychotherapist*))) OR ((Keywords: (staff*))) OR ((Keywords: (personnel*))) OR ((Keywords: (employee*))) OR ((Keywords: (doctor*)))) OR (((abstract: (worker*))) OR ((abstract: (psychoeducator*))) OR ((abstract: (psycho-educator*))) OR ((abstract: (educator*))) OR ((abstract: (nurse*))) OR ((abstract: (criminologist*))) OR ((abstract: (psychologist*))) OR ((abstract: (clinician*))) OR ((abstract: (practitioner*))) OR ((abstract: (physician*))) OR ((abstract: (professional*))) OR ((abstract: (provider*))) OR ((abstract: (conselor))) OR ((abstract: (conselors))) OR ((abstract: (counselor))) OR ((abstract: (counselers))) OR ((abstract: (caregiver*))) OR ((abstract: (giver*))) OR ((abstract: (therapist*))) OR ((abstract: (psychotherapist*))) OR ((abstract: (staff*))) OR ((abstract: (personnel*))) OR ((abstract: (employee*))) OR ((abstract: (doctor*)))))) AND (((((Keywords: (marijuana))) OR ((Keywords: (marihuana))) OR ((Keywords: (cannabis))) OR ((Keywords: (hashish))) OR ((Keywords: (Pot))) OR ((Keywords: (weed))) OR ((Keywords: (tetrahydrocannabinol))) OR ((Keywords: (THC))) OR ((Keywords: (CDB))) OR ((Keywords: (cannabidiol))) OR ((Keywords: (cannabinoid))) OR ((Keywords: (cannabinoids)))) OR (((abstract: (marijuana))) OR ((abstract: (marihuana))) OR ((abstract: (cannabis))) OR ((abstract: (hashish))) OR ((abstract: (Pot))) OR ((abstract: (weed))) OR ((abstract: (tetrahydrocannabinol))) OR ((abstract: (THC))) OR ((abstract: (CDB))) OR ((abstract: (cannabidiol))) OR ((abstract: (cannabinoid))) OR ((abstract: (cannabinoids))))) OR ((((MeSH: (Marijuana Smoking))))) OR ((((MeSH: (Marijuana Abuse))))) OR ((((MeSH: ("Marijuana Use"))))) OR ((((MeSH: (Cannabis))))))) AND ((Year: [1990 TO 9999]))) NOT ((((Keywords: (afghanistan/)) OR (Keywords: (africa/)) OR (Keywords: (africa, northern/)) OR (Keywords: (africa, central/)) OR (Keywords: (africa, eastern/)) OR (Keywords: ("africa south of the sahara" /)) OR (Keywords: (africa, southern/)) OR (Keywords: (africa, western/)) OR (Keywords: (albania/)) OR (Keywords: (algeria/)) OR (Keywords: (andorra/)) OR (Keywords: (angola/)) OR (Keywords: ("antigua and barbuda" /)) OR (Keywords: (argentina/)) OR (Keywords: (armenia/)) OR (Keywords: (azerbaijan/)) OR (Keywords: (bahamas/)) OR (Keywords: (bahrain/)) OR (Keywords: (bangladesh/)) OR (Keywords: (barbados/)) OR (Keywords: (belize/)) OR (Keywords: (benin/)) OR (Keywords: (bhutan/)) OR (Keywords: (bolivia/)) OR (Keywords: (borneo/)) OR (Keywords: ("bosnia and herzegovina" /)) OR

For peer review only - http://bmjopen.bmj.com/site/about/guidelines.xhtml

(Keywords: (botswana/)) OR (Keywords: (brazil/)) OR (Keywords: (brunei/)) OR (Keywords: (bulgaria/)) OR (Keywords: (burkina faso/)) OR (Keywords: (burundi/)) OR (Keywords: (cabo verde/)) OR (Keywords: (cambodia/)) OR (Keywords: (cameroon/)) OR (Keywords: (central african republic/)) OR (Keywords: (chad/)) OR (Keywords: (exp china/)) OR (Keywords: (comoros/)) OR (Keywords: (congo/)) OR (Keywords: (cote d'ivoire/)) OR (Keywords: (croatia/)) OR (Keywords: (cuba/)) OR (Keywords: ("democratic republic of the congo" /)) OR (Keywords: (cyprus/)) OR (Keywords: (djibouti/)) OR (Keywords: (dominica/)) OR (Keywords: (dominican republic/)) OR (Keywords: (ecuador/)) OR (Keywords: (egypt/)) OR (Keywords: (el salvador/)) OR (Keywords: (equatorial guinea/)) OR (Keywords: (eritrea/)) OR (Keywords: (eswatini/)) OR (Keywords: (ethiopia/)) OR (Keywords: (fiji/)) OR (Keywords: (gabon/)) OR (Keywords: (gambia/)) OR (Keywords: ("georgia (republic)" /)) OR (Keywords: (ghana/)) OR (Keywords: (grenada/)) OR (Keywords: (guatemala/)) OR (Keywords: (guinea/)) OR (Keywords: (guinea-bissau/)) OR (Keywords: (guyana/)) OR (Keywords: (haiti/)) OR (Keywords: (honduras/)) OR (Keywords: (independent state of samoa/)) OR (Keywords: (exp india/)) OR (Keywords: (indian ocean islands/)) OR (Keywords: (indochina/)) OR (Keywords: (indonesia/)) OR (Keywords: (iran/)) OR (Keywords: (iraq/)) OR (Keywords: (jamaica/)) OR (Keywords: (jordan/)) OR (Keywords: (kazakhstan/)) OR (Keywords: (kenya/)) OR (Keywords: (kosovo/)) OR (Keywords: (kuwait/)) OR (Keywords: (kyrgyzstan/)) OR (Keywords: (laos/)) OR (Keywords: (lebanon/)) OR (Keywords: (liechtenstein/)) OR (Keywords: (lesotho/)) OR (Keywords: (liberia/)) OR (Keywords: (libya/)) OR (Keywords: (madagascar/)) OR (Keywords: (malaysia/)) OR (Keywords: (malawi/)) OR (Keywords: (mali/)) OR (Keywords: (malta/)) OR (Keywords: (mauritania/)) OR (Keywords: (mauritius/)) OR (Keywords: (mekong valley/)) OR (Keywords: (melanesia/)) OR (Keywords: (micronesia/)) OR (Keywords: (monaco/)) OR (Keywords: (mongolia/)) OR (Keywords: (montenegro/)) OR (Keywords: (morocco/)) OR (Keywords: (mozambique/)) OR (Keywords: (myanmar/)) OR (Keywords: (namibia/)) OR (Keywords: (nepal/)) OR (Keywords: (nicaragua/)) OR (Keywords: (niger/)) OR (Keywords: (nigeria/)) OR (Keywords: (oman/)) OR (Keywords: (pakistan/)) OR (Keywords: (palau/)) OR (Keywords: (exp panama/)) OR (Keywords: (papua new guinea/)) OR (Keywords: (paraguay/)) OR (Keywords: (peru/)) OR (Keywords: (philippines/)) OR (Keywords: (qatar/)) OR (Keywords: ("republic of belarus" /)) OR (Keywords: ("republic of north macedonia" /)) OR (Keywords: (romania/)) OR (Keywords: (exp russia/)) OR (Keywords: (rwanda/)) OR (Keywords: ("saint kitts and nevis" /)) OR (Keywords: (saint lucia/)) OR (Keywords: ("saint vincent and the grenadines" /)) OR (Keywords: ("sao tome and principe" /)) OR (Keywords: (saudi arabia/)) OR (Keywords: (serbia/)) OR (Keywords: (sierra leone/)) OR (Keywords: (senegal/)) OR (Keywords: (seychelles/)) OR (Keywords: (singapore/)) OR (Keywords: (somalia/)) OR (Keywords: (south africa/)) OR (Keywords: (south sudan/)) OR (Keywords: (sri lanka/)) OR (Keywords: (sudan/)) OR (Keywords: (suriname/)) OR (Keywords: (syria/)) OR (Keywords: (taiwan/)) OR (Keywords: (tajikistan/)) OR (Keywords: (tanzania/)) OR (Keywords: (thailand/)) OR (Keywords: (timor-leste/)) OR (Keywords: (togo/)) OR (Keywords: (tonga/)) OR (Keywords: ("trinidad and tobago" /)) OR (Keywords: (tunisia/)) OR (Keywords: (turkmenistan/)) OR (Keywords: (uganda/)) OR (Keywords: (ukraine/)) OR (Keywords: (united arab emirates/)) OR (Keywords: (uruguay/)) OR (Keywords: (uzbekistan/)) OR (Keywords: (vanuatu/)) OR (Keywords: (venezuela/)) OR (Keywords: (vietnam/)) OR (Keywords: (west indies/)) OR (Keywords: (yemen/)) OR (Keywords: (zambia/)) OR (Keywords: (zimbabwe/)))) AND Any Field: - (((Keywords: (australasia/)) OR (Keywords: (exp australia/)) OR (Keywords: (austria/)) OR (Keywords: (baltic states/)) OR (Keywords: (belgium/)) OR (Keywords: (exp canada/)) OR (Keywords: (chile/)) OR (Keywords: (colombia/)) OR (Keywords: (costa rica/)) OR

For peer review only

(Keywords: (czech republic/)) OR (Keywords: (exp denmark/)) OR (Keywords: (estonia/)) OR (Keywords: (europe/)) OR (Keywords: (finland/)) OR (Keywords: (exp france/)) OR (Keywords: (exp germany/)) OR (Keywords: (greece/)) OR (Keywords: (hungary/)) OR (Keywords: (iceland/)) OR (Keywords: (ireland/)) OR (Keywords: (israel/)) OR (Keywords: (exp italy/)) OR (Keywords: (exp japan/)) OR (Keywords: (korea/)) OR (Keywords: (latvia/)) OR (Keywords: (lithuania/)) OR (Keywords: (luxembourg/)) OR (Keywords: (mexico/)) OR (Keywords: (netherlands/)) OR (Keywords: (new zealand/)) OR (Keywords: (north america/)) OR (Keywords: (exp norway/)) OR (Keywords: (poland/)) OR (Keywords: (portugal/)) OR (Keywords: (exp "republic of korea" /)) OR (Keywords: ("scandinavian and nordic countries" /)) OR (Keywords: (slovakia/)) OR (Keywords: (slovenia/)) OR (Keywords: (spain/)) OR (Keywords: (sweden/)) OR (Keywords: (switzerland/)) OR (Keywords: (turkey/)) OR (Keywords: (exp united kingdom/)) OR (Keywords: (exp united states/)) OR (Keywords: (European Union/)) OR (Keywords: (Developed Countries/)))))

For peer review only

Appendix 3: CINAHL

#	Question	Results
S1	MW "harm# reduction#"	4,889
S2	MW "risk# reduction#"	0
S3	TI protective N2 strateg* OR AB protective N2 strateg* OR SU protective N2 strateg*	1,167
S4	SU ((reduc* or minimi*) N5 (harm# or harmful or risk#)) OR TI ((reduc* or minimi*) N5 (harm# or harmful or risk#)) OR AB ((reduc* or minimi*) N5 (harm# or harmful or risk#))	96,037
S5	S1 OR S2 OR S3 OR S4	97,005
S6	MW "Health Personnel#"	112,080
S7	MW "Social Worker#"	11,786
S8	MW Counselors	4,471
S9	MW "Health Occupation#"	5,719
S10	MW "Allied Health Occupation#"	0
S11	MW "Allied Health Personnel#"	4,781
S12	TI (worker or psychoeducator or psycho-educator or educator or nurse or criminologist or psychologist or clinician or practitioner or physician or professional or provider or counse#lor or counse#lors or caregiver or giver or therapist or psychotherapist or staff or personnel or employee or doctor) OR AB (worker or psychoeducator or psycho-educator or educator or nurse or criminologist or psychologist or clinician or practitioner or physician or professional or provider or counse#lor or counse#lors or caregiver or giver or therapist or psychotherapist or staff or personnel or employee or doctor) OR SU (worker or psychoeducator or psycho-educator or educator or nurse or criminologist or psychologist or clinician or practitioner or physician or professional or provider or counse#lor or counse#lors or caregiver or giver or therapist or psychotherapist or staff or personnel or employee or doctor)	1,762,879
S13	S6 OR S7 OR S8 OR S9 OR S10 OR S11 OR S12	1,765,867

For peer review only

S14	MW Cannabis	11,138
S15	MW Marijuana	2,219
S16	TI (mari#uana or cannabis or hashish or Pot or weed or tetrahydrocannabinol or THC or CDB or cannabidiol or cannabinoid#) OR AB (mari#uana or cannabis or hashish or Pot or weed or tetrahydrocannabinol or THC or CDB or cannabidiol or cannabinoid#) OR SU (mari#uana or cannabis or hashish or Pot or weed or tetrahydrocannabinol or THC or CDB or cannabidiol or cannabinoid#)	26,526
S17	S14 OR S15 OR S16	26,526
S18	S5 AND S13 AND S17	147
S19	S5 AND S13 AND S17	147
S20	S5 AND S13 AND S17	147
S21	S5 AND S13 AND S17	72
S22	SU afghanistan/ or africa/ or africa, northern/ or africa, central/ or africa, eastern/ or "africa south of the sahara"/ or africa, southern/ or africa, western/ or albania/ or algeria/ or andorra/ or angola/ or "antigua and barbuda"/ or argentina/ or armenia/ or azerbaijan/ or bahamas/ or bahrain/ or bangladesh/ or barbados/ or belize/ or benin/ or bhutan/ or bolivia/ or borneo/ or "bosnia and herzegovina"/ or botswana/ or brazil/ or brunei/ or bulgaria/ or burkina faso/ or burundi/ or cabo verde/ or cambodia/ or cameroon/ or central african republic/ or chad/ or exp china/ or comoros/ or congo/ or cote d'ivoire/ or croatia/ or cuba/ or "democratic republic of the congo"/ or cyprus/ or djibouti/ or dominica/ or dominican republic/ or ecuador/ or egypt/ or el salvador/ or equatorial guinea/ or eritrea/ or eswatini/ or ethiopia/ or fiji/ or gabon/ or gambia/ or "georgia (republic)"/ or ghana/ or grenada/ or guatemala/ or guinea/ or guinea-bissau/ or guyana/ or haiti/ or honduras/ or independent state of samoa/ or exp india/ or indian ocean islands/ or indochina/ or indonesia/ or iran/ or iraq/ or jamaica/ or jordan/ or kazakhstan/ or kenya/ or kosovo/ or kuwait/ or kyrgyzstan/ or laos/ or lebanon/ or liechtenstein/ or lesotho/ or liberia/ or libya/ or madagascar/ or malaysia/ or malawi/ or	159,916

For peer review only

	mali/ or malta/ or mauritania/ or mauritius/ or mekong valley/ or melanesia/ or micronesia/ or monaco/ or mongolia/ or montenegro/ or morocco/ or mozambique/ or myanmar/ or namibia/ or nepal/ or nicaragua/ or niger/ or nigeria/ or oman/ or pakistan/ or palau/ or exp panama/ or papua new guinea/ or paraguay/ or peru/ or philippines/ or qatar/ or "republic of belarus"/ or "republic of north macedonia"/ or romania/ or exp russia/ or rwanda/ or "saint kitts and nevis"/ or saint lucia/ or "saint vincent and the grenadines"/ or "sao tome and principe"/ or saudi arabia/ or serbia/ or sierra leone/ or senegal/ or seychelles/ or singapore/ or somalia/ or south africa/ or south sudan/ or sri lanka/ or sudan/ or suriname/ or syria/ or taiwan/ or tajikistan/ or tanzania/ or thailand/ or timor-leste/ or togo/ or tonga/ or "trinidad and tobago"/ or tunisia/ or turkmenistan/ or uganda/ or ukraine/ or united arab emirates/ or uruguay/ or uzbekistan/ or vanuatu/ or venezuela/ or vietnam/ or west indies/ or yemen/ or zambia/ or zimbabwe/	
S23	SU australasia/ or exp australia/ or austria/ or baltic states/ or belgium/ or exp canada/ or chile/ or colombia/ or costa rica/ or czech republic/ or exp denmark/ or estonia/ or europe/ or finland/ or exp france/ or exp germany/ or greece/ or hungary/ or iceland/ or ireland/ or israel/ or exp italy/ or exp japan/ or korea/ or latvia/ or lithuania/ or luxembourg/ or mexico/ or netherlands/ or new zealand/ or north america/ or exp norway/ or poland/ or portugal/ or exp "republic of korea"/ or "scandinavian and nordic countries"/ or slovakia/ or slovenia/ or spain/ or sweden/ or switzerland/ or turkey/ or exp united kingdom/ or exp united states/ OR European Union/ OR Developed Countries/	158,684
S24	S22 NOT S23	154,938
S25	S21 NOT S24	66

For peer review only

Appendix 4: Web of Science

#	Question	Results
#1	TS=(harm reduction OR risk reduction behavior OR (protective NEAR/2 strateg*) OR ((reduc* OR minimi*) NEAR/5 (harm OR harms OR harmful OR risk OR risks)))	329,523
#2	TS=(cannabis OR marijuana OR mari$uana OR hashish OR pot OR weed OR tetrahydrocannabinol OR THC OR CDB OR cannabidiol OR cannabinoid$)	305,377
#3	TS=(health personnel OR social worker$ OR counselor$ OR health occupation$ OR worker$ OR psychoeducator$ OR psycho-educator$ OR nurse$ OR criminologist$ OR psychologist$ OR clinician$ OR practitioner$ OR physician$ OR professional$ OR provider$ OR co$nselor OR co$nselors OR caregiver$ OR giver$ OR therapist$ OR psychotherapist$ OR staff$ OR personnel$ OR employee$ OR doctor$)	2,593,408
#4	#1 AND #2 AND #3	331
#5	#4 and English or French (Languages)	324
#6	#5 and [Countries/Regions filter: pays de l'OCDE]	300

For peer review only

Appendix 5: Embase

1	Harm Reduction/	8189
2	Risk Reduction Behavior/	115695
3	(protective adj2 strateg*).ab,kf,ti.	3987
4	((reduc* or minimi*) adj5 (harm? or harmful or risk?)).ab,kf,ti.	345174
**5**	1 or 2 or 3 or 4	414447
6	exp Health Personnel/	1843814
7	Social Workers/	12409
8	Counselors/	3692
9	exp Health Occupations/	24456
10	exp Allied Health Occupations/	392205
11	exp Allied Health Personnel/	567029
12	(worker? or (psychoeducator? or psycho-educator?) or educator? or nurse? or criminologist? or psychologist? or clinician? or practitioner? or physician? or professional? or provider? or co?nselor or co?nselors or caregiver? or giver? or therapist? or psychotherapist? or staff? or personnel? or employee? or doctor?).ab,kf,ti.	2640146
**13**	6 or 7 or 8 or 9 or 10 or 11 or 12	3761559
14	Cannabis/	40937
15	"Marijuana Use"/	12636
16	Marijuana Abuse/	6445
17	Marijuana Smoking/	4026
18	(mari?uana or cannabis or has?hish or Pot or weed or tetrahydrocannabinol or THC or CDB or cannabidiol or cannabinoid?).ab,kf,ti.	129932
**19**	14 or 15 or 16 or 17 or 18	142957
20	5 and 13 and 19	569
21	limit 32 to yr="1990 -Current"	569
22	limit 21 to (english or french)	560
23	Filtre OCDE	520

For peer review only

Appendix 6: Sociological Abstracts

(((ti(health personnel OR social worker OR social workers OR counselor OR counselors OR conselor OR conselors OR health occupation OR health occupations OR worker* OR psychoeducator* OR psycho-educator* OR educator* OR nurse* OR criminologist* OR psychologist* OR clinician* OR professional* OR provider* OR caregiver* OR giver* OR therapist* OR psychotherapist* OR staff* OR personnel* OR employee* OR doctor*) OR ab(health personnel OR social worker OR social workers OR counselor OR counselors OR conselor OR conselors OR health occupation OR health occupations OR worker* OR psychoeducator* OR psycho-educator* OR educator* OR nurse* OR criminologist* OR psychologist* OR clinician* OR professional* OR provider* OR caregiver* OR giver* OR therapist* OR psychotherapist* OR staff* OR personnel* OR employee* OR doctor*) OR su(health personnel OR social worker OR social workers OR counselor OR counselors OR conselor OR conselors OR health occupation OR health occupations OR worker* OR psychoeducator* OR psycho-educator* OR educator* OR nurse* OR criminologist* OR psychologist* OR clinician* OR professional* OR provider* OR caregiver* OR giver* OR therapist* OR psychotherapist* OR staff* OR personnel* OR employee* OR doctor*)) AND (ti(cannabis OR marijuana OR mari*uana OR hashish OR pot OR weed OR tetrahydrocannabinol OR THC OR CDB OR cannabidiol OR cannabinoid*) OR ab(cannabis OR marijuana OR mari*uana OR hashish OR pot OR weed OR tetrahydrocannabinol OR THC OR CDB OR cannabidiol OR cannabinoid*) OR su(cannabis OR marijuana OR mari*uana OR hashish OR pot OR weed OR tetrahydrocannabinol OR THC OR CDB OR cannabidiol OR cannabinoid*)) AND ((ti((reduc* OR minimi*) NEAR/5 (harm OR harms OR harmful OR risk OR risks)) OR ab((reduc* OR minimi*) NEAR/5 (harm OR harms OR harmful OR risk OR risks)) OR su((reduc* OR minimi*) NEAR/5 (harm OR harms OR harmful OR risk OR risks))) OR (ti(protective NEAR/2 strateg*) OR ab(protective NEAR/2 strateg*) OR su(protective NEAR/2 strateg*)))) AND la.exact("ENG")) AND yr(1990-2029)

For peer review only

Appendix 7: Google Scholar

cannabis intitle:nurse OR intitle:clinician OR intitle:practitioner OR intitle:physician OR intitle:caregiver OR intitle:giver OR intitle:doctor "harm reduction" = 105

cannabis intitle:nurses OR intitle:clinicians OR intitle:practitioners OR intitle:physicians OR intitle:caregivers OR intitle:givers OR intitle:doctors "harm reduction" = 242

cannabis intitle:psychoeducator OR intitle:educator OR intitle:therapist OR intitle:psychotherapist OR intitle:criminologist OR intitle:psychologist "harm reduction" = 19

cannabis intitle:psychoeducators OR intitle:educators OR intitle:therapists OR intitle:psychotherapists OR intitle:criminologists OR intitle:psychologists "harm reduction" = 53

cannabis intitle:worker OR intitle:staff OR intitle:personnel OR intitle:employee OR intitle:professional OR intitle:provider OR intitle:counselor OR intitle:conselor "harm reduction" = 136

cannabis intitle: workers OR intitle:staffs OR intitle:personnels OR intitle:employees OR intitle:professionals OR intitle:providers OR intitle:counselors OR intitle:conselors "harm reduction" = 5

cannabis intitle:infirmier OR intitle:médecin OR intitle:clinicien OR intitle:psychoéducateur OR intitle:éducateur OR intitle:thérapeute OR intitle:psychothérapeute OR intitle:criminologue OR intitle:psychologue “reduction des méfaits” = 1

cannabis intitle:travailleur OR intitle:personnel OR intitle:employé OR intitle:professionnel OR intitle:conseiller OR intitle:intervenant “reduction des méfaits” = 5

cannabis intitle:infirmiers OR intitle:médecins OR intitle:cliniciens OR intitle:psychoéducateurs OR intitle:éducateurs OR intitle:thérapeutes OR intitle:psychothérapeutes OR intitle:criminologues OR intitle:psychologues “reduction des méfaits” = 3

cannabis intitle:travailleurs OR intitle:personnels OR intitle:employés OR intitle:professionnels OR intitle:conseillers OR intitle:intervenants “reduction des méfaits” = 12

cannabis intitle:infirmière OR intitle:cliniciennne OR intitle:psychoéducatrice OR intitle:éducatrice OR intitle:criminologue OR intitle:psychologue “reduction des méfaits” = 2

cannabis intitle:travailleuse OR intitle:employée OR intitle:professionnelle OR intitle:conseillère OR intitle:intervenante “reduction des méfaits” = 1

cannabis intitle:infirmières OR intitle:cliniciennes OR intitle:psychoéducatrices OR intitle:éducatrices OR intitle:criminologues OR intitle:psychologues “reduction des méfaits” = 2

cannabis intitle:travailleuses OR intitle:employées OR intitle:professionnelles OR intitle:conseillères OR intitle:intervenantes “reduction des méfaits” = 2

marijuana intitle:nurse OR intitle:clinician OR intitle:practitioner OR intitle:physician OR intitle:caregiver OR intitle:giver OR intitle:doctor "harm reduction" = 139

marijuana intitle:nurses OR intitle:clinicians OR intitle:practitioners OR intitle:physicians OR intitle:caregivers OR intitle:givers OR intitle:doctors "harm reduction" = 238

marijuana intitle:psychoeducator OR intitle:educator OR intitle:therapist OR intitle:psychotherapist OR intitle:criminologist OR intitle:psychologist "harm reduction" = 19

For peer review only

marijuana intitle:psychoeducators OR intitle:educators OR intitle:therapists OR intitle:psychotherapists OR intitle:criminologists OR intitle:psychologists "harm reduction" = 48

marijuana intitle:worker OR intitle:staff OR intitle:personnel OR intitle:employee OR intitle:professional OR intitle:provider OR intitle:counselor OR intitle:conselor "harm reduction" = 140

marijuana intitle:workers OR intitle:staffs OR intitle:personnels OR intitle:employees OR intitle:professionals OR intitle:providers OR intitle:counselors OR intitle:conselors "harm reduction" = 573

marijuana intitle:infirmier OR intitle:médecin OR intitle:clinicien OR intitle:psychoéducateur OR intitle:éducateur OR intitle:thérapeute OR intitle:psychothérapeute OR intitle:criminologue OR intitle:psychologue “reduction des méfaits” = 1

marijuana intitle:travailleur OR intitle:personnel OR intitle:employé OR intitle:professionnel OR intitle:conseiller OR intitle:intervenant “reduction des méfaits” = 5

marijuana intitle:infirmiers OR intitle:médecins OR intitle:cliniciens OR intitle:psychoéducateurs OR intitle:éducateurs OR intitle:thérapeutes OR intitle:psychothérapeutes OR intitle:criminologues OR intitle:psychologues “reduction des méfaits” = 3

marijuana intitle:travailleurs OR intitle:personnels OR intitle:employés OR intitle:professionnels OR intitle:conseillers OR intitle:intervenants “reduction des méfaits” = 12

marijuana intitle:infirmière OR intitle:clinicienne OR intitle:psychoéducatrice OR intitle:éducatrice OR intitle:criminologue OR intitle:psychologue “reduction des méfaits” = 2

marijuana intitle:travailleuse OR intitle:employée OR intitle:professionnelle OR intitle:conseillère OR intitle:intervenante “reduction des méfaits” = 1

marijuana intitle:infirmières OR intitle:cliniciennes OR intitle:psychoéducatrices OR intitle:éducatrices OR intitle:criminologues OR intitle:psychologues “reduction des méfaits” = 2

marijuana intitle:travailleuses OR intitle:employées OR intitle:professionnelles OR intitle:conseillères OR intitle:intervenantes “reduction des méfaits” = 2

For peer review only

Appendix 8: Google Web

cannabis intitle:nurse OR intitle:clinician OR intitle:practitioner OR intitle:physician OR intitle:caregiver OR intitle:giver OR intitle:doctor "harm reduction" filetype:pdf =

cannabis intitle:nurses OR intitle:clinicians OR intitle:practitioners OR intitle:physicians OR intitle:caregivers OR intitle:givers OR intitle:doctors "harm reduction" filetype:pdf =

cannabis intitle:psychoeducator OR intitle:educator OR intitle:therapist OR intitle:psychotherapist OR intitle:criminologist OR intitle:psychologist "harm reduction" filetype:pdf =

cannabis intitle:psychoeducators OR intitle:educators OR intitle:therapists OR intitle:psychotherapists OR intitle:criminologists OR intitle:psychologists "harm reduction" filetype:pdf =

cannabis intitle:worker OR intitle:staff OR intitle:personnel OR intitle:employee OR intitle:professional OR intitle:provider OR intitle:counselor OR intitle:conselor "harm reduction" filetype:pdf =

cannabis intitle: workers OR intitle:staffs OR intitle:personnels OR intitle:employees OR intitle:professionals OR intitle:providers OR intitle:counselors OR intitle:conselors "harm reduction" filetype:pdf =

cannabis intitle:infirmier OR intitle:médecin OR intitle:clinicien OR intitle:psychoéducateur OR intitle:éducateur OR intitle:thérapeute OR intitle:psychothérapeute OR intitle:criminologue OR intitle:psychologue “reduction des méfaits” filetype:pdf =

cannabis intitle:travailleur OR intitle:personnel OR intitle:employé OR intitle:professionnel OR intitle:conseiller OR intitle:intervenant “reduction des méfaits” filetype:pdf =

cannabis intitle:infirmiers OR intitle:médecins OR intitle:cliniciens OR intitle:psychoéducateurs OR intitle:éducateurs OR intitle:thérapeutes OR intitle:psychothérapeutes OR intitle:criminologues OR intitle:psychologues “reduction des méfaits” filetype:pdf =

cannabis intitle:travailleurs OR intitle:personnels OR intitle:employés OR intitle:professionnels OR intitle:conseillers OR intitle:intervenants “reduction des méfaits” filetype:pdf =

cannabis intitle:infirmière OR intitle:clinicienne OR intitle:psychoéducatrice OR intitle:éducatrice OR intitle:criminologue OR intitle:psychologue “reduction des méfaits” filetype:pdf =

cannabis intitle:travailleuse OR intitle:employée OR intitle:professionnelle OR intitle:conseillère OR intitle:intervenante “reduction des méfaits” filetype:pdf =

cannabis intitle:infirmières OR intitle:cliniciennes OR intitle:psychoéducatrices OR intitle:éducatrices OR intitle:criminologues OR intitle:psychologues “reduction des méfaits” filetype:pdf =

cannabis intitle:travailleuses OR intitle:employées OR intitle:professionnelles OR intitle:conseillères OR intitle:intervenantes “reduction des méfaits” filetype:pdf =

marijuana intitle:nurse OR intitle:clinician OR intitle:practitioner OR intitle:physician OR intitle:caregiver OR intitle:giver OR intitle:doctor "harm reduction" filetype:pdf =

marijuana intitle:nurses OR intitle:clinicians OR intitle:practitioners OR intitle:physicians OR intitle:caregivers OR intitle:givers OR intitle:doctors "harm reduction" filetype:pdf =

For peer review only

marijuana intitle:psychoeducator OR intitle:educator OR intitle:therapist OR intitle:psychotherapist OR intitle:criminologist OR intitle:psychologist "harm reduction" filetype:pdf =

marijuana intitle:psychoeducators OR intitle:educators OR intitle:therapists OR intitle:psychotherapists OR intitle:criminologists OR intitle:psychologists "harm reduction" filetype:pdf =

marijuana intitle:worker OR intitle:staff OR intitle:personnel OR intitle:employee OR intitle:professional OR intitle:provider OR intitle:counselor OR intitle:conselor "harm reduction" filetype:pdf =

marijuana intitle:workers OR intitle:staffs OR intitle:personnels OR intitle:employees OR intitle:professionals OR intitle:providers OR intitle:counselors OR intitle:conselors "harm reduction" filetype:pdf =

marijuana intitle:infirmier OR intitle:médecin OR intitle:clinicien OR intitle:psychoéducateur OR intitle:éducateur OR intitle:thérapeute OR intitle:psychothérapeute OR intitle:criminologue OR intitle:psychologue “reduction des méfaits” filetype:pdf =

marijuana intitle:travailleur OR intitle:personnel OR intitle:employé OR intitle:professionnel OR intitle:conseiller OR intitle:intervenant “reduction des méfaits” filetype:pdf =

marijuana intitle:infirmiers OR intitle:médecins OR intitle:cliniciens OR intitle:psychoéducateurs OR intitle:éducateurs OR intitle:thérapeutes OR intitle:psychothérapeutes OR intitle:criminologues OR intitle:psychologues “reduction des méfaits” filetype:pdf =

marijuana intitle:travailleurs OR intitle:personnels OR intitle:employés OR intitle:professionnels OR intitle:conseillers OR intitle:intervenants “reduction des méfaits” filetype:pdf =

marijuana intitle:infirmière OR intitle:clinicienne OR intitle:psychoéducatrice OR intitle:éducatrice OR intitle:criminologue OR intitle:psychologue “reduction des méfaits” filetype:pdf =

marijuana intitle:travailleuse OR intitle:employée OR intitle:professionnelle OR intitle:conseillère OR intitle:intervenante “reduction des méfaits” filetype:pdf =

marijuana intitle:infirmières OR intitle:cliniciennes OR intitle:psychoéducatrices OR intitle:éducatrices OR intitle:criminologues OR intitle:psychologues “reduction des méfaits” filetype:pdf =

marijuana intitle:travailleuses OR intitle:employées OR intitle:professionnelles OR intitle:conseillères OR intitle:intervenantes “reduction des méfaits” filetype:pdf =

For peer review only

Appendix 9: BASE

tit:(nurse* clinician* practitioner* physician* caregiver* giver* doctor* psychoeducator* educator* therapist* psychotherapist* criminologist* psychologist* worker* staff* personnel* employee* professional* provider* counselor*) subj:(cannabis marijuana) subj:"harm reduction" = 4

tit:(infirmier* OU médecin* OU clinicien* OU psychoéducat* OU éducat* OU criminologue* OU psychologue* OU travailleu* OU employé* OU professionnel* OU conseill* OU intervenant*) subj:cannabis subj:marijuana subj:"réduction des méfaits" = 0

For peer review only

Appendix 10: Érudit

(Titre, résumé, mots-clés : harm reduction OU harm minimisation OU risk reduction OU risks reduction OU risk minimisation OU réduction des méfaits OU réduction des risques) ET (Titre, résumé, mots-clés : cannabis OU marijuana) ET (Titre, résumé, mots-clés : nurse* OU clinician* OU practitioner* OU physician* OU caregiver* OU giver* OU doctor* OU psychoeducator* OU educator* OU therapist* OU psychotherapist* OU criminologist* OU psychologist* OU worker* OU staff* OU personnel* OU employee* OU professional* OU provider* OU counselor* OU infirmier* OU médecin* OU clinicien* OU psychoéducat* OU éducat* OU criminologue* OU psychologue* OU travailleu* OU employé* OU professionnel* OU conseill* OU intervenant*) = 27

For peer review only

BMJ Open

Facilitators of and barriers to healthcare providers' adoption of harm reduction in cannabis use: a scoping review protocol

Journal:	*BMJ Open*
Manuscript ID	bmjopen-2023-078427.R2
Article Type:	Protocol
Date Submitted by the Author:	19-Mar-2024
Complete List of Authors:	Haddad, Roula; Université de Montréal Faculté des Arts et des Sciences, Department of Psychology<br>Dagenais, Christian; Université de Montréal Faculté des Arts et des Sciences, Department of Psychology<br>Huynh, Christophe ; Institut Universitaire sur les Dépendances, Centre intégré universitaire de santé et de services sociaux du Centre-Sud-de-l'Île-de-Montréal ; Université de Montreal Département de Psychiatrie<br>Fallu, Jean-Sébastien; University of Montreal, Psychoeducation; Centre intégré universitaire de santé et de services sociaux du Centre-Sud-de-l'Île-de-Montréal du Québec, Centre de recherche en santé publique (CReSP)
**Primary Subject Heading**:	Addiction
Secondary Subject Heading:	Public health, Research methods
Keywords:	PUBLIC HEALTH, MENTAL HEALTH, Substance misuse < PSYCHIATRY, Health Services

SCHOLARONE™
Manuscripts

Facilitators of and barriers to healthcare providers' adoption of harm reduction in cannabis use: a scoping review protocol

Roula Haddad[1], Christian Dagenais[1], Christophe Huynh[2,3], Jean-Sébastien Fallu[2,4,5]

[1] Department of Psychology, Université de Montréal

[2] University Institute on Addictions, Centre intégré universitaire de santé et de services sociaux du Centre-Sud-de-l'Île-de-Montréal

[3] Department of Psychiatry and Addictology, Université de Montréal

[4] School of Psychoeducation, Université de Montréal

[5] Centre de recherche en santé publique (CReSP), Centre intégré universitaire de santé et de services sociaux du Centre-Sud-de-l'Île-de-Montréal

Correspondence to:
Roula Haddad
roula.haddad@umontreal.ca

ABSTRACT

Introduction: The high prevalence of cannabis use and the potential for negative effects indicate the need for effective prevention strategies and treatment of people who use cannabis (PWUC). Studies show that harm reduction (HR) in cannabis use is effective in minimising the harmful consequences of the substance. However, health professionals often misunderstand it and resist its adoption due to various obstacles. To our knowledge, there has been no review of the scientific literature on the factors that facilitate or hinder practitioners' adoption of HR in cannabis use. To fill this gap, we aim to identify, through a scoping review, facilitators and barriers to healthcare providers' adoption of HR in cannabis use in Organisation for Economic Cooperation and Development (OECD) countries.

Methods and analysis: Our methodology will be guided by the six-step model initially proposed by Arksey and O'Malley (2005). The search strategy will be executed on different databases (Medline, PsycINFO, CINAHL, Web of Science, Embase, Sociological Abstracts, Érudit, BASE, Google Web, and Google Scholar) and will cover articles published between 1990 and October

For peer review only

2022. Empirical studies published in French or English in an OECD country and identifying factors that facilitate or hinder healthcare providers' adoption of HR in cannabis use, will be included. Reference lists of the selected articles as well as relevant systematic reviews will be scanned to identify any missed publications by the electronic searches.

Ethics and dissemination: Ethics approval is not required. The results will be disseminated through various activities (e.g., publication in peer-reviewed journals, conferences, webinars, and knowledge translation activities). The results will also allow us to conduct a future study aiming to develop and implement a knowledge translation process among healthcare practitioners working with youth in Quebec in order to enhance their adoption of HR in cannabis use.

Strengths and limitations of this study

- The search strategy was co-developed by two information management specialists in the addiction and knowledge translation fields.
- The search strategy will aim to retrieve published articles on several health databases and unpublished studies found in the grey literature.
- Two reviewers will independently select the studies to be included in the scoping review throughout the entire study selection stage.
- The included studies will be limited to those published in French or English.
- The included studies will be limited to those published in OECD countries.

Keywords: *harm reduction; cannabis; knowledge translation; healthcare*

For peer review only

INTRODUCTION

Cannabis use

Psychoactive substances (e.g., cannabis, alcohol, nicotine) are defined as substances whose use affects mental processes (e.g., perception, cognition, emotions, mood) and behaviors without necessarily leading to addiction (1). After tobacco and alcohol, cannabis represents the third most consumed psychoactive substance globally among adults and youth (2-5). Canada remains among the developed countries with the highest rates of cannabis use among young people and adults, with high prevalence in Quebec and multiple patterns of use (e.g., smoking, eating, vaporising, vaping) (2, 5-9). In 2023, 26% of those 16 years of age and older reported using cannabis in the 12 months prior to the study, with a higher prevalence among those aged between 20 to 24 years old (10).

The lifetime cumulative probability of transitioning from use to dependence was found to be the lowest for people who use cannabis (PWUC) (8.9%) compared to other substances such as nicotine (67.5%), alcohol (22.7%), and cocaine (20.9%); this can be due to several factors such as infrequency in use, consumption of cannabis with low rates of δ-9-tetrahydrocannabinol (THC), availability, legality, and social acceptability (9, 11-15). Even with long-term exposure to cannabis, PWUC do not necessarily develop severe problems or a cannabis-dependence (5, 15, 16).

However, PWUC intensively or at-risk populations (i.e., pregnant persons, people presenting respiratory problems, a mental health comorbidity, having an early onset of continued tobacco use or concurrently smoking tobacco and cannabis) may develop a cannabis use disorder or experience harm at several levels (5, 12, 16-19). These harms may include a decreased academic or professional performance, a cognitive impairment, a deterioration of mental health (e.g., development of psychosis or depression), an increased occurrence of risky behaviors (e.g., cannabis-impaired driving), etc. (3-5, 12, 14, 18, 20-22). It is important to specify that a severe cannabis-induced mental health condition (e.g., psychosis) might occur among only 2% of PWUC (5). Despite the minimal probability of leading to potentially serious adverse consequences, the high prevalence of cannabis use as well as the potential harms that might be experienced, make it essential to implement effective intervention programs among PWUC (7, 12, 15, 17).

For peer review only

Abstinence approach

To address this reality, prevention and treatment programs based on the abstinence approach (i.e., total elimination of cannabis use), have been widely implemented (11, 13). The emergence of these programs has been also influenced by policies such as the "War on Drugs" (23). The abstinence-based model forms the basis of many programs developed to prevent or treat problematic cannabis use and has been applied with at-risk or marginalised populations (e.g., youth in the foster care system) (21, 24). Despite its potential to decrease the frequency or amount of substance use, the abstinence approach presents limited evidence to support its effectiveness and has been criticised for various reasons (21, 24-26). First, it does not provide PWUC with the necessary skills to identify and mitigate the harms associated with their use (21, 27). Second, the abstinence approach tends to focus more on the negative consequences of use through strategies that evoke fear, without necessarily taking into consideration the social context of cannabis use (20, 21). Third, the risks of relapse and dropout in these programs are also found to be high, leaving a significant number of PWUC for whom this goal remains unattainable (20, 28). Given these limitations of abstinence-oriented programs, other alternative and more flexible treatments, such as harm reduction (HR), are essential to reduce and mitigate cannabis-related harms (14, 21, 28, 29).

Harm reduction (HR)

Description

HR in cannabis use aims to minimise the harmful consequences of the substance at the individual, psychological, legal, and social levels among PWUC (19, 20, 28, 29). It offers a public health framework based on values of pragmatism and humanism, as it does not view substance use through a moral lens, but as an inevitable societal fact of long-standing (4, 11, 30, 31). Whether among adults or adolescents, elimination of substance use is unrealistic at the population level and should be the individual's choice without being imposed, as it represents an unwanted and impractical goal for some (e.g., in case of recreational or occasional use, in case of dual diagnosis combining psychiatric and substance use disorders) (27-29, 32, 33). HR in cannabis use also seeks to equip PWUC to make responsible and rational decisions and learn ways to reduce the negative consequences associated with their consumption (11, 12, 20, 27, 29, 31). To this end, HR clarifies the notion of safe substance use that is determined by the interaction of three components: the individual (height, weight, gender, physical and mental health status, state of mind, etc.); the drug

For peer review only

(quantity, frequency of use, tolerance to the product, combination with other products, quality, etc.); and the setting (location, time of day, interpersonal relationships, conflicts, laws, etc.) (4, 20, 27). In addition, HR takes into account the personal characteristics of PWUC (impulsivity, sensation-seeking, etc.) and addresses their potential ambivalence about stopping substance use, their feelings of failure upon relapse, their engagement in treatment, their social skills, their emotional regulation, etc. (20). In the "Lower Risk Cannabis Use Guidelines", Fischer (5) updated the initial recommendations to reduce the harms of cannabis use. These recommendations include delaying the initiation of cannabis use until late adolescence or the completion of puberty, consuming low-potency cannabis products, avoiding deep inhalation, using legal and quality-controlled cannabis products, etc. (5).

HR effectiveness

Interventions based on HR for non-injected drugs have been studied across various populations (e.g., youth, adults, people in housing programs presenting mental health conditions) and have shown promising results in decreasing the negative consequences associated with substance use (20, 28).

Several studies showed the effectiveness of HR strategies and current school-based HR programs (e.g., SHAHRP in the United Kingdom and SCIDUA in Canada) in developing safer attitudes toward substance use and in reducing negative consequences related to use (21, 22, 34, 35). The effectiveness of HR among youth who use cannabis has led the University Institute on Addictions (*Institut universitaire sur les dépendances*) in Quebec to recommend it as an intervention modality among this clientele (36). In addition, effective early interventions targeting college and university students with at-risk cannabis use are those that reduce the harms associated with cannabis use (17, 36-38). Moreover, Palfai (38) found that students participating in a web-based HR intervention (Marijuana eCHECKUP TO GO) showed statistically significant lower results in peer cannabis use after six months ($f^2 = .11$, [B = 7.45 (3.34), $p < .05$]).

HR in cannabis use was also found effective among adults (17, 39). After delivering a brief HR intervention for PWUC, Fischer (17) found significant reductions in risk outcome indicators only among the experimental group. At the 12th month follow-up, a change was maintained for "deep inhalation/breath-holding" (experimental group: Q = 13.1; $p < .05$; control group: Q = 4.8; $p > .05$), and "driving after cannabis use" (experimental group: Q = 9.3; $p < .05$; control group: Q = 0.9; $p >$

For peer review only

.05) (17). Furthermore, without completely abstaining from the substance, a functional improvement can be reached by treatment-seeking adults presenting a cannabis use disorder when they reduce the frequency and/or quantity of cannabis use (39). Reduction in the frequency of cannabis use was associated with a decrease in depression (F = 2.76, p = .04, n_p^2 =.04), anxiety (F = 3.70, p =.01, n_p^2 = .05), and cannabis-related problems (F = 8.95, p < .001, n_p^2 = .12) (39). In addition, a decrease in the quantity of cannabis consumption was associated with a decrease in anxiety (F = 3.02, p = .03, n_p^2 =.04) and cannabis-related problems (F = 3.24, p = .02, n_p^2 = .05) (39). A systematic review also highlighted that the adoption of HR strategies by PWUC acts as a protective factor for people with poor mental health, low self-regulation, high impulsivity, and high negative urgency (22).

Acceptability of harm reduction

Despite its proven effectiveness, the acceptability and applicability of HR by health and social services practitioners remain limited, and various factors facilitate or hinder its adoption (21, 40-42). MacCoun (43) showed that, among practitioners who did not adopt HR, some had based their decision on moral grounds, regardless of its effectiveness. Various barriers limit its use by practitioners. First, ambiguities in its conceptualisation play an influential role; for example, some practitioners perceive HR as sending the wrong message, i.e., one of tolerating or even encouraging substance use (27, 28, 30, 44). Some do not perceive total cessation of substance use as a legitimate goal that could be achieved through HR (45). Also, there is often confusion between reducing use (frequency, quantity, etc.) and reducing harm (modifying consumption practices, such as contexts and mixtures, to reduce consequences) (30). These misconceptions point to the need for awareness-raising, training, and supervision of practitioners interested in this approach (28). Second, the adoption of HR can be hindered by ethical dilemmas, as well as by issues related to the personal and collective values of healthcare workers and the therapeutic model of abstinence (30). Indeed, it runs counter to traditional treatments by tolerating risky behaviors and accepting that HR in drug use is a legitimate outcome (30, 44). Practitioners may also fear the emergence of legal, social, and health problems among their clients (28). Third, its adoption may be limited by contextual barriers, such as lack of funding, stigma that undermines demand for care, resistance from local jurisdictions, and lack of services and trained personnel, particularly in the mental health sector (41). Healthcare providers' resistance to applying HR in cannabis use leads to limited

For peer review only

knowledge and utilisation of effective HR techniques and guidelines among PWUC (5, 25). Among the study's participants (i.e., PWUC), Kruger (25) found that less than half of the participants believed that the listed HR techniques were effective and reported applying them. However, several factors seen as HR benefits have been found to facilitate its implementation by healthcare providers, such as: broadening the spectrum of acceptable goals, improving clients' decision-making skills, creating positive and quality relationships, and managing relapses (28). A study by Sharp (41) showed that clarifying the positive impacts of HR at the community level (e.g., safety) and ensuring the availability of resources could increase the likelihood of its adoption.

Purpose

Despite its proven effectiveness, several reasons might hinder HR adoption by health professionals among PWUC (30, 41). However, to date, there has been no review of the scientific literature that identifies the factors that facilitate or limit the adoption of HR in cannabis use. To fill this gap, we aim to identify, through a scoping review, facilitators and barriers to healthcare providers' adoption of HR in cannabis use.

METHODS AND ANALYSIS

This study will follow the methodological steps of scoping reviews (46). This type of review has become more prevalent in recent years and is a type of knowledge synthesis review (46, 47). There is no universal definition for scoping reviews; however, a variety of factors distinguish them from other types of knowledge synthesis (48). First, scoping reviews address broad research questions and include studies with different designs and multiple sources of evidence to provide an overview of the available knowledge around a concept (49). On the contrary, a systematic review following Cochrane standards explores more specific research questions based on detailed inclusion and exclusion criteria (50-52). Second, while assessment of the methodological quality of included studies is recommended for scoping reviews, it is not mandatory, whereas assessment of the risk of bias of included studies is required for Cochrane-type systematic reviews (50, 52). Researchers undertake scoping reviews for a variety of reasons: 1) to review research activity in a given area; 2) to determine the feasibility and appropriateness of conducting a systematic review based on Cochrane standards; 3) to summarise and disseminate the results of existing research on a topic; and/or 4) to identify a gap in the literature and draw conclusions regarding a topic (49). Our

For peer review only

methodological choice is underpinned by three of these reasons: once the research activity around the topic has been consulted, the findings will be summarised and used to support a second study aimed at disseminating knowledge to practitioners through a knowledge translation process. This will also allow us to identify gaps in the literature and draw conclusions related to the topic.

Arksey and O'Malley (49) were the first to propose a six-step model for conducting scoping reviews. Our methodology will be guided by this model, which was later refined by Levac (48) and revised by members of the Joanna Briggs Institute (50). The six stages we will follow are:

- Stage 1: Determining the research question and the objective
- Stage 2: Identifying relevant studies
- Stage 3: Selecting studies
- Stage 4: Charting the data
- Stage 5: Collating, summarising, and reporting the results
- Stage 6: Conducting a consultation exercise

The research protocol will be reported using the Preferred Reporting Items for Systematic Reviews and Meta-Analyses Extension for Scoping Reviews (PRISMA-ScR) grid (47). This grid is an extension of the PRISMA grid originally developed for Cochrane-type systematic reviews and helps to ensure the transparency and reproducibility of the study (47, 50).

Stage 1: Determining the research question and the objective

Arksey and O'Malley's (49) model suggests that scoping reviews should begin not only with identifying research questions but also with clarifying the resulting objectives (48, 50). Our scoping review is exploratory and aims to identify facilitators and barriers to healthcare providers' adoption of HR in cannabis use in OECD countries. Based on the Population–Concept–Context (PCC) model, which allows the broad scope of the study to be respected without specifying restrictive inclusion criteria (50), we formulated the research question: What factors influence providers (population) in the healthcare field (context) to adopt HR in cannabis use (concept)? Specific research questions associated with the components of the PCC model were also identified.

- Question 1, related to the concept and context components: What are the facilitators and barriers to healthcare providers' adoption of HR in cannabis use?
- Question 2, related to the population component: Who is the clientele of the providers identified in the studies?

For peer review only

- Question 3, related to the concept component: What is the definition of HR in cannabis use?

Stage 2: Identifying relevant studies

Search strategy

The search strategy was developed through an iterative process. A senior librarian at the Quebec Addiction Library (*Bibliothèque québécoise sur les dépendances*) first developed three search strategies with different concepts and ran them on the Medline database. The first 50 results of each strategy were consulted, which led us to opt the one that grouped key terms related to the following concepts: harm reduction, clinicians, and cannabis (see Supplemental Appendix). The search strategy was then reviewed by a second information professional working in the RENARD Team for Knowledge Translation who, in turn, adapted it to the selected databases. The final search strategy executed on all the databases was then validated by the RENARD Team information specialist. The Peer Review of Electronic Search Strategies (PRESS) tool served as a guide for the librarians in this process (53). The search strategy executed on each database is presented in Supplemental Appendix. All search strategies were executed on October 10th, 2022.

Information sources

To identify relevant published and unpublished studies for inclusion in the study, various sources of information will be reviewed (46, 49, 50). With the guidance of the two librarians, the search strategy will be executed on the leading health and intervention databases: Medline, PsycINFO, Cumulative Index to Nursing and Allied Health Literature (CINAHL), Web of Science, Embase, and Sociological Abstracts. To explore the grey literature (e.g., theses, research reports, etc.), the search strategy will be adapted to the Google Web and Google Scholar search engines, as well as the Érudit (French database) and BASE databases. All documents identified will be entered into Zotero software for the research team members to access. To identify any missed publication by the electronic searches, reference lists of selected articles will be manually searched and relevant systematic reviews will be scanned to identify their included studies.

For peer review only

Stage 3: Study selection

After running the search strategy on the selected databases and completing the second stage, all identified duplicates will be removed. The remaining documents will then be entered into Covidence software. Two reviewers will independently select relevant documents for inclusion by reading the titles and abstracts of the identified studies. Their decisions will be based on the specified inclusion and exclusion criteria. The inter-rater agreement between the reviewers will first be calculated and then they will meet regularly to resolve selection conflicts and refine the eligibility criteria as needed. After this first step, the documents selected and deemed potentially relevant will then be the subject of the second step, the full-text reading. Again, the two reviewers will independently record their choices on Covidence. The inter-rater agreement for this step will be calculated and the reviewers will then resolve any new conflicts. A third reviewer will be called upon as needed for any conflict resolution. This will complete the document selection stage, whose steps will be presented in a PRISMA diagram (54).

Inclusion and exclusion criteria

Inclusion and exclusion criteria have been specified and will be fine-tuned as needed to select relevant studies (Table 1). Using this process, empirical studies of quantitative, qualitative, or mixed designs, identifying factors that facilitate or hinder healthcare providers' adoption of HR in cannabis use, will be selected. To facilitate comparison and generalisation of the results to the Quebec context, the review will be limited to studies conducted in any of the 38 OECD countries. Articles published between 1990 and October 2022 will be included, as it was early in the 1990s that HR gained international prominence and its scope of application began to expand. Papers not meeting these inclusion criteria will be excluded. Systematic reviews will be excluded to avoid duplication and ensure equal representation of the selected papers; the executed search strategy might have already captured studies included in a potentially relevant systematic review. However, systematic reviews' reference lists will be examined to identify additional relevant studies that might be selected.

For peer review only

Table 1. Inclusion and exclusion criteria

Criteria	Inclusion criteria	Exclusion criteria
**Type of study**	- Empirical study: quantitative, qualitative, or mixed	- Study that does not present empirical results (e.g., theoretical study, conceptual framework, etc.) or knowledge review (e.g., systematic or literature review)<br>- Interviews
**Type of documents**	- Peer-reviewed scientific articles, research reports, dissertations, theses	- Books and practice guides
**Conceptual framework**	- Harm reduction (HR) in cannabis use<br>- Cannabis risk reduction<br>- Non-abstinence in cannabis use	- Another conceptual framework
**Objective**	- Identification of factors[1] facilitating or hindering practitioners' adoption of HR[2] in cannabis use	- Evaluation of the effectiveness of interventions based on HR<br>OR<br>- Stakeholder perceptions of the use of cannabis as an HR strategy to circumvent the effects of other drugs<br>OR<br>- Attitudes toward decriminalisation of cannabis
**Psychoactive substance being studied**	- Marijuana, hashish, or cannabis for non-medical purposes<br>- "Drug" if cannabis is part of its conceptualisation in the study	- Any substance other than marijuana, hashish, or non-medical cannabis (e.g., tobacco, alcohol, medical cannabis, MDMA, Ecstasy)<br>- Study that focuses on "performance and image enhancing drugs" or "crack" or "new psychoactive substances"
**Target population**	- Practitioners[3] working in the health field<br>- Practitioners in training	- PWUC[4]
**Country of study**	- OECD countries	- Other countries
**Publication date**	- From 1990 onwards	- Before 1990
**Language**	- French and/or English	- Languages other than French or English or text not available

[1] "Factors" include perceptions, beliefs, facilitators, obstacles, oppositions, attitudes, opinions, barriers, biases, motivations, preferences, determinants, incentives, influences, and perspectives on the adoption of HR in cannabis use, as well as its acceptability and receptivity.

[2] "Approach" refers to strategies, interventions, practices, services, methods, techniques, treatments, programs, or guides for the HR approach in cannabis use.

[3] "Practitioners" include healthcare personnel, professionals, or practitioners, allied healthcare personnel, professionals, or practitioners, social workers, counselors, psychoeducators, educators, nurses, criminologists, psychologists, clinicians, caregivers, therapists, psychotherapists, and physicians.

[4] Studies addressing the views of people who use cannabis (PWUC) regarding HR or its adoption by practitioners will be excluded.

Stage 4: Charting the data

To analyse the selected studies on a common basis, specific variables of interest will be identified based on the research questions (49). These will form the components of summary sheets that will be developed in Microsoft Excel and used to extract results (Table 2). This method is an analytical descriptive recording of the data (49, 50, 55). The first author (RH) will extract the data from the included studies and create the summary sheets. The research supervisor (CD) will validate the summary sheets throughout the process and ensure their alignment with the research questions (50).

Table 2. Summary sheets

General variables	Specific variables
**General characteristics of the study**	Study title
	Author(s)
	Language of publication
	Date of publication
	Period of publication
	Journal
	Type of article
	Full reference
	Country of study
	Psychoactive substance under study
	The legal status of cannabis in the country of study
**Introduction**	Main concepts
	Definition of the main concept: HR in cannabis use
	Research question(s)
	Objective(s)
	Hypothesis
**Methodology**	Study design

For peer review only - http://bmjopen.bmj.com/site/about/guidelines.xhtml

	Target population<br>Place of work of the target population<br>Inclusion criteria of participants<br>Recruitment method<br>Sample size<br>Country of origin of participants<br>The clientele of the population recruited<br>Data collection method<br>Analysis steps
**Results**	Sample presentation<br>Key findings: 1) facilitators and 2) barriers to practitioners' adoption of HR in cannabis use<br>Secondary outcomes or other results
**Conclusion**	Study strengths<br>Study limitations<br>Gaps in the literature and future research needs

Stage 5: Collating, summarising, and reporting the results

Based on the eligibility criteria, studies deemed relevant will be collected, summarised, and reported. They will be subjected to 1) a numerical analysis, and 2) a narrative organisation encompassing a descriptive qualitative analysis (47, 49, 50). A numerical analysis of the scope, nature, and distribution of the included studies will be performed to various characteristics: date of publication, country of origin of the studies, and type of document. Subsequently, a narrative organisation of the results will be produced to identify the relationships between the data and the research questions. The summary sheets will be combined, tabulated, and synthesised, and will then be subjected to a descriptive qualitative analysis (Table 3).

Table 3. Narrative organisation of the included studies

Data	Study 1	Study 2	Study …
Type of publication			
Date of publication			
Country of study			
Legal status of cannabis in the country of the study			
Definition of HR to cannabis use			
Design of the study			

For peer review only

Target population
Place of work of the target population
The clientele of the target population
Data collection method
Key findings:<br>- Facilitators or enabling conditions<br>- Barriers or adverse conditions
Secondary outcomes

Stage 6: Consultation exercise

Expert consultation is an optional step that promotes methodological rigor in scoping reviews (49). In this study, the project's supervisor and co-researchers will be solicited as consultants. Members of the RENARD team and researchers involved in the field of substance use and harm reduction will be consulted to help clarify findings and validate the resulting recommendations (49). Consultations will be conducted: 1) after preliminary results have been obtained, and 2) after analyses of the results have been completed.

Patient and public involvement

None.

ETHICS AND DISSEMINATION

To our knowledge, this is the first scoping review on factors that facilitate or hinder healthcare providers' adoption of HR in cannabis use. Other reviews have studied HR interventions in general among practitioners working with a specific population. This study will provide a clear picture of the factors at play when adopting HR, and the results could potentially be generalisable to OECD countries. The present study is exempt from ethics approval because it involves no patient or personal data collection. The results are expected to be ready by March 2024. They will be disseminated, alongside the scoping review protocol, through various activities (e.g., publication in peer-reviewed journals, conferences, webinars, posters, *Three Minute Thesis* competition).

After completing the scoping review, we will be able to conduct a future study aiming to implement a knowledge translation plan among practitioners working with youth in Quebec to enhance and expand their adoption of HR in cannabis use.

For peer review only

Acknowledgements: We wish to thank Karine Bélanger, senior librarian at the *Bibliothèque québécoise sur les dépendances* in Quebec for developing the initial search strategy used in this scoping review. We also wish to thank Julie Desnoyers, the information specialist affiliated with the RENARD Team for Knowledge Translation at Université de Montréal, for validating the search strategy and adapting it to the selected databases. Finally, we want to thank Donna Riley for editing the manuscript.

Contributors: RH, CD, CH, and JSF conceptualised the study. RH drafted the protocol. CD, CH, and JSF critically revised the manuscript. RH wrote the final draft manuscript and all the authors approved it.

Funding: This work was supported by the *Ministère de la Santé et des Services sociaux* (MSSS), *Fonds de recherche du Québec – Santé* (FRQS), and *Fonds de recherche du Québec – Société et culture* (FRQSC) (Grant number: 2023-0PTR-322652). A grant obtained by the *Centre de recherche en santé publique* (CReSP) helped in partially covering the revision and publication fees of this scoping review protocol.

Competing interests: None declared.

For peer review only

REFERENCES

1. World Health Organization. Drugs (psychoactive) [online]; 2022. https://www.who.int/health-topics/drugs-psychoactive#tab=tab_1 (accessed 13 Jul 2023).

2. Adinoff B, Cooper ZD. Cannabis legalization: progress in harm reduction approaches for substance use and misuse. Am J Drug Alcohol Abuse 2019;45(6):707–12. doi: 10.1080/00952990.2019.1680683.

3. Jiloha RC. Prevention, early intervention, and harm reduction of substance use in adolescents. Indian J Psychiatry 2017;59(1):111–8. doi: 10.4103%2F0019-5545.204444.

4. Melamede R. Harm reduction-the cannabis paradox. Harm Reduct J 2005;2(1):17. doi: 10.1186/1477-7517-2-17.

5. Fischer B, Robinson T, Bullen C, Curran V, Jutras-Aswad D, Medina-Mora ME, et al. Lower-Risk Cannabis Use Guidelines (LRCUG) for reducing health harms from non-medical cannabis use: A comprehensive evidence and recommendations update. Int J Drug Policy. 2022;99:103381.

6. Poulin C, Elliott D. Alcohol, tobacco and cannabis use among Nova Scotia adolescents: implications for prevention and harm reduction. CMAJ 1997;156(10):1387–93. PMID: **9164396**.

7. Gouvernement du Québec. Plan d'action interministériel en dépendances 2018-2028 – Prévenir, réduire et traiter les conséquences associées à la consommation de substances psychoactives, à la pratique de jeux de hasard et d'argent et à l'utilisation d'Internet [online]. 2018. https://publications.msss.gouv.qc.ca/msss/document-002078/ (accessed 13 Jul 2023).

8. Zuckermann AM, Gohari MR, Romano I, et al. Changes in cannabis use modes among Canadian youth across recreational cannabis legalization: data from the COMPASS prospective cohort study. Addict Behav 2021;122:107025. doi: 10.1016/j.addbeh.2021.107025.

9. Fischer B, Russell C, Sabioni P, Van Den Brink W, Le Foll B, Hall W, et al. Lower-risk cannabis use guidelines: a comprehensive update of evidence and recommendations. Am J Public Health. 2017;107(8):e1-e12.

For peer review only

10. Government of Canada. Canadian Cannabis Survey 2023: Summary [online]. 2023. https://www.canada.ca/fr/sante-canada/services/drogues-medicaments/cannabis/recherches-donnees/enquete-canadienne-cannabis-2023-sommaire.html#s2-2 (accessed 13 Feb 2024).

11. Hathaway AD, Erickson PG. Drug reform principles and policy debates: harm reduction prospects for cannabis in Canada. J Drug Issues 2003;33(2):465–95. doi: 10.1177/002204260303300209.

12. Swift W, Copeland J, Lenton S. Cannabis and harm reduction. Drug Alcohol Rev 2000;19(1):101–12. doi: 10.1080/09595230096200.

13. Stockings E, Hall WD, Lynskey M, et al. Prevention, early intervention, harm reduction, and treatment of substance use in young people. Lancet Psychiatry 2016;3(3):280–96. doi: 10.1016/S2215-0366(16)00002-X.

14. Englund A, Freeman TP, Murray RM, McGuire P. Can we make cannabis safer? Lancet Psychiatry. 2017;4(8):643-8. doi: 10.1016/S2215-0366(17)30075-5.

15. Lopez-Quintero C, de los Cobos JP, Hasin DS, Okuda M, Wang S, Grant BF, et al. Probability and predictors of transition from first use to dependence on nicotine, alcohol, cannabis, and cocaine: Results of the National Epidemiologic Survey on Alcohol and Related Conditions (NESARC). Drug Alcohol Depend. 2011;115(1-2):120-30. doi: 10.1016/j.drugalcdep.2010.11.004.

16. Hindocha C, Shaban ND, Freeman TP, Das RK, Gale G, Schafer G, et al. Associations between cigarette smoking and cannabis dependence: a longitudinal study of young cannabis users in the United Kingdom. Drug Alcohol Depend. 2015;148:165-71. doi: 10.1016/j.drugalcdep.2015.01.004.

17. Fischer B, Jones W, Shuper P, et al. 12-month follow-up of an exploratory 'brief intervention' for high-frequency cannabis users among Canadian university students. Subst Abuse Treat Prev Policy 2012;7(1):15. doi: 10.1186/1747-597X-7-15.

18. Lemyre A, Poliakova N, Bélanger RE. The relationship between tobacco and cannabis use: a review. Subst Use Misuse. 2019;54(1):130-45. doi: 10.1080/10826084.2018.1512623.

For peer review only

19. Winer JM, Yule AM, Hadland SE, Bagley SM. Addressing adolescent substance use with a public health prevention framework: the case for harm reduction. Ann Med. 2022;54(1):2123-36. doi: 10.1080/07853890.2022.2104922.

20. Bagot KS, Kaminer Y. Harm reduction for youth in treatment for substance use disorders: one size does not fit all. Curr Addict Rep 2018;5(3):379–85. doi: 10.1007/s40429-018-0217-8.

21. Jenkins EK, Slemon A, Haines-Saah RJ. Developing harm reduction in the context of youth substance use: insights from a multi-site qualitative analysis of young people's harm minimization strategies. Harm Reduct J 2017;14(1):53. doi: 10.1186/s12954-017-0180-z.

22. González-Ponce BM, Rojas-Tejada AJ, Carmona-Márquez J, Lozano-Rojas ÓM, Díaz-Batanero C, Fernández-Calderón F. Harm reduction strategies among university students who use alcohol and cannabis, and related psychological variables: a systematic review. J Psychoactive Drugs. 2022;54(5):403-18. doi: 10.1080/02791072.2021.2023240.

23. Buchanan J, Young L. The war on drugs – a war on drug users? Drugs (Abingdon Engl) 2000;7(4):409–22. doi: 10.1080/dep.7.4.409.422.

24. Howe EJ, Bishop LD, Torraville BS, Rowe EC, Kinzel E, Donnan JR. Canadian cannabis education resources to support youth health literacy: A scoping review and environmental scan. Health Educ J. 2023;82(7):766-78. doi: 10.1177/00178969231191085.

25. Kruger JS, Kruger D, Collins RL. Knowledge and practice of harm reduction strategies among people who report frequent cannabis use. Health Promot Pract. 2021;22(1):24-30. doi: 10.1177/1524839920923999.

26. Watson TM, Valleriani J, Hyshka E, Rueda S. Cannabis legalization in the provinces and territories: missing opportunities to effectively educate youth? Can J Public Health. 2019;110:472-5. doi: 10.17269/s41997-019-00209-0.

27. Fallu J-S. Réduction des méfaits: histoire, concepts, pratiques, développements et enjeux [online]. Journée de la toxicomanie. Oral presentation, Journée de la toxicomanie, 23 Nov 2017; St-Liguori, Québec, Canada. https://www.reseautoxicomanie.com/wp-content/uploads/2017/12/Conférence-JS-Fallu_PNMVH-23-septembre-2017.pdf (accessed 13 Jul 2023).

For peer review only

28. Mancini MA, Linhorst DM, Broderick F, et al. Challenges to implementing the harm reduction approach. J Soc Work Pract Addict 2008;8(3):380–408. doi: 10.1080/15332560802224576.

29. Karoll BR. Applying social work approaches, harm reduction, and practice wisdom to better serve those with alcohol and drug use disorders. J Soc Work 2010;10(3):263–81. doi: 10.1177/1468017310363635.

30. Fallu J-S, Brisson P. La réduction des méfaits liés à l'usage des drogues: historique, état des lieux, enjeux. In: Massé R, Mondou I, eds. Réduction des méfaits et tolérance en santé publique: enjeux éthiques et politique. Presses de l'Université Laval 2013:105–27.

31. Ritter A, Cameron J. A review of the efficacy and effectiveness of harm reduction strategies for alcohol, tobacco and illicit drugs. Drug Alcohol Rev 2006;25(6):611–24. doi: 10.1080/09595230600944529.

32. Lau N, Sales P, Averill S, Murphy F, et al. A safer alternative: cannabis substitution as harm reduction. Drug Alcohol Rev 2015;34(6):654–9. doi: 10.1111/dar.12275.

33. Tsemberis S, Gulcur L, Nakae M. Housing first, consumer choice, and harm reduction for homeless individuals with a dual diagnosis. Am J Public Health 2004;94(4):651–6. doi: 10.2105/AJPH.94.4.651.

34. McBride N, Farringdon F, Midford R, et al. Harm minimization in school drug education: Final results of the School Health and Alcohol Harm Reduction Project (SHAHRP). Addiction 2004;99(3):278–91. doi: 10.1111/j.1360-0443.2003.00620.x.

35. Poulin C, Nicholson J. Should harm minimization as an approach to adolescent substance use be embraced by junior and senior high schools?: empirical evidence from an integrated school- and community-based demonstration intervention addressing drug use among adolescents. Int J Drug Policy 2005;16(6):403–14. doi: 10.1016/j.drugpo.2005.11.001.

36. Bertrand K, Blanchette-Martin N, Dufour M, et al. Projet IP-Jeunes : recenser et promouvoir les critères d'efficacité reconnus en intervention précoce auprès des jeunes présentant des comportements à risque quant à la consommation d'alcool et d'autres drogues, à la pratique des jeux de hasard et d'argent et à l'utilisation d'internet. Synthèse des résultats préliminaires, 2019-2020 [online]. IUD: Institut universitaire sur les dépendances; 2020.

For peer review only

https://chairejeunesse.ca/documentation/projet-ip-jeunes-recenser-et-promouvoir-les-criteres-defficacite-reconnus-en-intervention-precoce-aupres-des-jeunes-presentant-des-comportements-a-risque-quant-a-la-consommation-da/ (accessed 13 Jul 2023).

37. Grossbard JR, Mastroleo NR, Kilmer JR, et al. Substance use patterns among first-year college students: secondary effects of a combined alcohol intervention. J Subst Abuse Treat 2010;39(4):384–90. doi: 10.1016/j.jsat.2010.07.001.

38. Palfai TP, Saitz R, Winter M, et al. Web-based screening and brief intervention for student marijuana use in a university health center: pilot study to examine the implementation of eCHECKUP TO GO in different contexts. Addict Behav 2014;39(9):1346–52. doi: 10.1016/j.addbeh.2014.04.025.

39. Sherman BJ, Sofis MJ, Borodovsky JT, Gray KM, McRae-Clark AL, Budney AJ. Evaluating cannabis use risk reduction as an alternative clinical outcome for cannabis use disorder. Psychol Addict Behav. 2022;36(5):505. doi: 10.1037/adb0000760.

40. Fallu J-S, Gilbert-Lambert MH, D'Arcy Dubois L, et al. Prévention de la consommation à risque ou problématique de substances psychoactives chez les jeunes hébergés en institution au Québec [online]. Centre intégré universitaire de santé et de services sociaux du Centre-Sud-de-l'Île-de-Montréal (Québec). Direction de l'enseignement universitaire et de la recherche. Institut universitaire sur les dépendances. 2022. https://iud.quebec/fr/etudes-et-recherche/programme-sur-lusage-et-les-dependances-aux-substances-puds/publications (accessed 13 Jul 2023).

41. Sharp A, Barnett JT, Vroom EB. Community perceptions of harm reduction and its implications for syringe exchange policy. J Drug Issues 2020;50(4):507–23. doi: 10.1177/0022042620932289.

42. Lindenfeld Z, Franz B, Cronin C, Chang JE. Hospital adoption of harm reduction and risk education strategies to address substance use disorders. Am J Drug Alcohol Abuse. 2023;49(2):206-15. doi: 10.1080/00952990.2023.2169832.

43. MacCoun RJ. Toward a psychology of harm reduction. Am Psychol 1998;53(11):1199–1208. doi: 10.1037/0003-066X.53.11.1199.

For peer review only

44. Mancini MA, Wyrick-Waugh W. Consumer and practitioner perceptions of the harm reduction approach in a community mental health setting. Community Ment Health J 2013;49(1):14–24. doi: 10.1007/s10597-011-9451-4.

45. Koutroulis G. “That original tension”. Negotiating abstinence in clinicians’ accounts of harm reduction in nonresidential treatment of heroin withdrawal. J Subst Abuse Treat 2000;19(1):89–98. doi: 10.1016/S0740-5472(99)00087-2.

46. Peters MDJ, Godfrey CM, Khalil H, et al. Guidance for conducting systematic scoping reviews. Int J Evid Based Healthc 2015;13(3):141–6. doi: 10.1097/XEB.0000000000000050.

47. Tricco AC, Lillie E, Zarin W, et al. PRISMA extension for scoping reviews (PRISMA-ScR): checklist and explanation. Ann Intern Med 2018;169(7):467–73. doi: 10.7326/M18-0850.

48. Levac D, Colquhoun H, O’Brien KK. Scoping studies: advancing the methodology. Implement Sci 2010;5(1):69. doi: 10.1186/1748-5908-5-69.

49. Arksey H, O’Malley L. Scoping studies: towards a methodological framework. Int J Soc Res Methodol 2005;8(1):19–32. doi: 10.1080/1364557032000119616.

50. Peters MDJ, Godfrey C, McInerney P, et al. Chapter 11: Scoping reviews. In: Aromataris E, Munn Z, eds. JBI Manual for Evidence Synthesis. JBI, 2020. doi: 10.46658/JBIMES-20-12.

51. Peterson J, Pearce PF, Ferguson LA, et al. Understanding scoping reviews: definition, purpose, and process. J Am Assoc Nurse Pract 2017;29(1):12–6. doi: 10.1002/2327-6924.12380.

52. Pollock D, Tricco AC, Peters MD, et al. Methodological quality, guidance, and tools in scoping reviews: a scoping review protocol. JBI Evid Synth 2022;20(4):1098–105. doi: 10.11124/JBIES-20-00570.

53. McGowan J, Sampson M, Salzwedel DM, et al. PRESS peer review of electronic search strategies: 2015 guideline statement. J Clin Epidemiol 2016;75:40–6. doi: 10.1016/j.jclinepi.2016.01.021.

54. Gedda M. Traduction française des lignes directrices PRISMA pour l’écriture et la lecture des revues systématiques et des méta-analyses [French translation of the PRISMA Reporting

For peer review only

Guidelines for writing and reading systematic reviews and meta-analyses]. Kinesither Rev 2015;15(157):39–44. doi: 10.1016/j.kine.2014.11.004.

55. Colquhoun HL, Levac D, O'Brien KK, et al. Scoping reviews: time for clarity in definition, methods, and reporting. J Clin Epidemiol 2014;67(12):1291–4. doi: 10.1016/j.jclinepi.2014.03.013.

For peer review only

Facilitators of and barriers to healthcare providers' adoption of harm reduction in cannabis use: a scoping review protocol

Roula Haddad, Christian Dagenais, Christophe Huynh, Jean-Sébastien Fallu

Supplemental Appendix: Search strategies executed on all databases

Appendix 1: Medline Search Strategy

#	Concept	Equations	Results
1	Harm reduction	Harm Reduction/	3898
2		Risk Reduction Behavior/	14132
3		(protective adj2 strateg*).ab,kf,ti.	2818
4		((reduc* or minimi*) adj5 (harm? or harmful or risk?)).ab,kf,ti.	239459
5		1 or 2 or 3 or 4	251821
6	Clinicians	exp Health Personnel/	588634
7		Social Workers/	971
8		Counselors/	541
9		exp Health Occupations/	1819039
10		exp Allied Health Occupations/	52626
11		exp Allied Health Personnel/	53169
12		(worker? or psychoeducator? or psycho-educator? or educator? or nurse? or criminologist? or psychologist? or clinician? or practitioner? or physician? or professional? or provider? or co?nselor or co?nselors or caregiver? or giver? or therapist? or psychotherapist? or staff? or personnel? or employee? or doctor?).ab,kf,ti.	2002544
13		6 or 7 or 8 or 9 or 10 or 11 or 12	3625104
14	Cannabis	Cannabis/	12307
15		"Marijuana Use"/	1689
16		Marijuana Abuse/	6905
17		Marijuana Smoking/	5425
18		(mari?uana or cannabis or hashish or Pot or weed or tetrahydrocannabinol or THC or CDB or cannabidiol or cannabinoid?).ab,kf,ti.	100005
19		14 or 15 or 16 or 17 or 18	102988
20	Strategies	("strateg*" or "approach*" or intervention? or prevent* or practice? or service? or "method*" or technique? or tactic? or co?nseling or treatment? or program? or "guide*").ab,kf,ti.	15074452
21	Combination of 3 concepts	5 and 13 and 19	272
22	Limit date	limit 21 to yr="1990 -Current"	272
23	Limit language	limit 22 to (english or french)	263
24	Filter OECD		
25	TOTAL	23 not 24	249
22	Combination of 4 concepts	5 and 13 and 19 and 20	251

Appendix 2: PsycINFO

(((((((title: (reduc*))) OR ((title: (minimi*)))) NEAR/5 (((title: (harm))) OR ((title: (harms))) OR ((title: (harmful))) OR ((title: (risk))) OR ((title: (risks)))) OR (((abstract: (reduc*))) OR ((abstract: (minimi*)))) NEAR/5 (((abstract: (harm))) OR ((abstract: (harms))) OR ((abstract: (harmful))) OR ((abstract: (risk))) OR ((abstract: (risks)))) OR (((Keywords: (reduc*))) OR ((Keywords: (minimi*)))) NEAR/5 (((Keywords: (harm))) OR ((Keywords: (harms))) OR ((Keywords: (harmful))) OR ((Keywords: (risk))) OR ((Keywords: (risks))))) OR ((((title: (protective NEAR/2 strateg*)))) OR (((abstract: (protective NEAR/2 strateg*)))) OR (((Keywords: (protective NEAR/2 strateg*))))) OR ((((MeSH: (Risk Reduction Behavior))))) OR ((((MeSH: (Harm Reduction)))))) AND (((((MeSH: (Health Personnel))))) OR ((((MeSH: (Social Workers))))) OR ((((MeSH: (Counselors))))) OR ((((MeSH: (Health Occupations))))) OR ((((MeSH: (Allied Health Occupations))))) OR ((((MeSH: (Allied Health Personnel))))) OR ((((Keywords: (worker*))) OR ((Keywords: (psychoeducator*))) OR ((Keywords: (psycho-educator*))) OR ((Keywords: (educator*))) OR ((Keywords: (nurse*))) OR ((Keywords: (criminologist*))) OR ((Keywords: (psychologist*))) OR ((Keywords: (clinician*))) OR ((Keywords: (practitioner*))) OR ((Keywords: (physician*))) OR ((Keywords: (professional*))) OR ((Keywords: (provider*))) OR ((Keywords: (conselor))) OR ((Keywords: (conselors))) OR ((Keywords: (counselor))) OR ((Keywords: (counselers))) OR ((Keywords: (caregiver*))) OR ((Keywords: (giver*))) OR ((Keywords: (therapist*))) OR ((Keywords: (psychotherapist*))) OR ((Keywords: (staff*))) OR ((Keywords: (personnel*))) OR ((Keywords: (employee*))) OR ((Keywords: (doctor*)))) OR (((abstract: (worker*))) OR ((abstract: (psychoeducator*))) OR ((abstract: (psycho-educator*))) OR ((abstract: (educator*))) OR ((abstract: (nurse*))) OR ((abstract: (criminologist*))) OR ((abstract: (psychologist*))) OR ((abstract: (clinician*))) OR ((abstract: (practitioner*))) OR ((abstract: (physician*))) OR ((abstract: (professional*))) OR ((abstract: (provider*))) OR ((abstract: (conselor))) OR ((abstract: (conselors))) OR ((abstract: (counselor))) OR ((abstract: (counselers))) OR ((abstract: (caregiver*))) OR ((abstract: (giver*))) OR ((abstract: (therapist*))) OR ((abstract: (psychotherapist*))) OR ((abstract: (staff*))) OR ((abstract: (personnel*))) OR ((abstract: (employee*))) OR ((abstract: (doctor*)))))) AND (((((Keywords: (marijuana))) OR ((Keywords: (marihuana))) OR ((Keywords: (cannabis))) OR ((Keywords: (hashish))) OR ((Keywords: (Pot))) OR ((Keywords: (weed))) OR ((Keywords: (tetrahydrocannabinol))) OR ((Keywords: (THC))) OR ((Keywords: (CDB))) OR ((Keywords: (cannabidiol))) OR ((Keywords: (cannabinoid))) OR ((Keywords: (cannabinoids)))) OR (((abstract: (marijuana))) OR ((abstract: (marihuana))) OR ((abstract: (cannabis))) OR ((abstract: (hashish))) OR ((abstract: (Pot))) OR ((abstract: (weed))) OR ((abstract: (tetrahydrocannabinol))) OR ((abstract: (THC))) OR ((abstract: (CDB))) OR ((abstract: (cannabidiol))) OR ((abstract: (cannabinoid))) OR ((abstract: (cannabinoids))))) OR ((((MeSH: (Marijuana Smoking))))) OR ((((MeSH: (Marijuana Abuse))))) OR ((((MeSH: ("Marijuana Use"))))) OR ((((MeSH: (Cannabis))))))) AND ((Year: [1990 TO 9999]))) NOT ((((Keywords: (afghanistan/)) OR (Keywords: (africa/)) OR (Keywords: (africa, northern/)) OR (Keywords: (africa, central/)) OR (Keywords: (africa, eastern/)) OR (Keywords: ("africa south of the sahara" /)) OR (Keywords: (africa, southern/)) OR (Keywords: (africa, western/)) OR (Keywords: (albania/)) OR (Keywords: (algeria/)) OR (Keywords: (andorra/)) OR (Keywords: (angola/)) OR (Keywords: ("antigua and barbuda" /)) OR (Keywords: (argentina/)) OR (Keywords: (armenia/)) OR (Keywords: (azerbaijan/)) OR (Keywords: (bahamas/)) OR (Keywords: (bahrain/)) OR (Keywords: (bangladesh/)) OR (Keywords: (barbados/)) OR (Keywords: (belize/)) OR (Keywords: (benin/)) OR (Keywords: (bhutan/)) OR (Keywords: (bolivia/)) OR (Keywords: (borneo/)) OR (Keywords: ("bosnia and herzegovina" /)) OR

(Keywords: (botswana/)) OR (Keywords: (brazil/)) OR (Keywords: (brunei/)) OR (Keywords: (bulgaria/)) OR (Keywords: (burkina faso/)) OR (Keywords: (burundi/)) OR (Keywords: (cabo verde/)) OR (Keywords: (cambodia/)) OR (Keywords: (cameroon/)) OR (Keywords: (central african republic/)) OR (Keywords: (chad/)) OR (Keywords: (exp china/)) OR (Keywords: (comoros/)) OR (Keywords: (congo/)) OR (Keywords: (cote d'ivoire/)) OR (Keywords: (croatia/)) OR (Keywords: (cuba/)) OR (Keywords: ("democratic republic of the congo" /)) OR (Keywords: (cyprus/)) OR (Keywords: (djibouti/)) OR (Keywords: (dominica/)) OR (Keywords: (dominican republic/)) OR (Keywords: (ecuador/)) OR (Keywords: (egypt/)) OR (Keywords: (el salvador/)) OR (Keywords: (equatorial guinea/)) OR (Keywords: (eritrea/)) OR (Keywords: (eswatini/)) OR (Keywords: (ethiopia/)) OR (Keywords: (fiji/)) OR (Keywords: (gabon/)) OR (Keywords: (gambia/)) OR (Keywords: ("georgia (republic)" /)) OR (Keywords: (ghana/)) OR (Keywords: (grenada/)) OR (Keywords: (guatemala/)) OR (Keywords: (guinea/)) OR (Keywords: (guinea-bissau/)) OR (Keywords: (guyana/)) OR (Keywords: (haiti/)) OR (Keywords: (honduras/)) OR (Keywords: (independent state of samoa/)) OR (Keywords: (exp india/)) OR (Keywords: (indian ocean islands/)) OR (Keywords: (indochina/)) OR (Keywords: (indonesia/)) OR (Keywords: (iran/)) OR (Keywords: (iraq/)) OR (Keywords: (jamaica/)) OR (Keywords: (jordan/)) OR (Keywords: (kazakhstan/)) OR (Keywords: (kenya/)) OR (Keywords: (kosovo/)) OR (Keywords: (kuwait/)) OR (Keywords: (kyrgyzstan/)) OR (Keywords: (laos/)) OR (Keywords: (lebanon/)) OR (Keywords: (liechtenstein/)) OR (Keywords: (lesotho/)) OR (Keywords: (liberia/)) OR (Keywords: (libya/)) OR (Keywords: (madagascar/)) OR (Keywords: (malaysia/)) OR (Keywords: (malawi/)) OR (Keywords: (mali/)) OR (Keywords: (malta/)) OR (Keywords: (mauritania/)) OR (Keywords: (mauritius/)) OR (Keywords: (mekong valley/)) OR (Keywords: (melanesia/)) OR (Keywords: (micronesia/)) OR (Keywords: (monaco/)) OR (Keywords: (mongolia/)) OR (Keywords: (montenegro/)) OR (Keywords: (morocco/)) OR (Keywords: (mozambique/)) OR (Keywords: (myanmar/)) OR (Keywords: (namibia/)) OR (Keywords: (nepal/)) OR (Keywords: (nicaragua/)) OR (Keywords: (niger/)) OR (Keywords: (nigeria/)) OR (Keywords: (oman/)) OR (Keywords: (pakistan/)) OR (Keywords: (palau/)) OR (Keywords: (exp panama/)) OR (Keywords: (papua new guinea/)) OR (Keywords: (paraguay/)) OR (Keywords: (peru/)) OR (Keywords: (philippines/)) OR (Keywords: (qatar/)) OR (Keywords: ("republic of belarus" /)) OR (Keywords: ("republic of north macedonia" /)) OR (Keywords: (romania/)) OR (Keywords: (exp russia/)) OR (Keywords: (rwanda/)) OR (Keywords: ("saint kitts and nevis" /)) OR (Keywords: (saint lucia/)) OR (Keywords: ("saint vincent and the grenadines" /)) OR (Keywords: ("sao tome and principe" /)) OR (Keywords: (saudi arabia/)) OR (Keywords: (serbia/)) OR (Keywords: (sierra leone/)) OR (Keywords: (senegal/)) OR (Keywords: (seychelles/)) OR (Keywords: (singapore/)) OR (Keywords: (somalia/)) OR (Keywords: (south africa/)) OR (Keywords: (south sudan/)) OR (Keywords: (sri lanka/)) OR (Keywords: (sudan/)) OR (Keywords: (suriname/)) OR (Keywords: (syria/)) OR (Keywords: (taiwan/)) OR (Keywords: (tajikistan/)) OR (Keywords: (tanzania/)) OR (Keywords: (thailand/)) OR (Keywords: (timor-leste/)) OR (Keywords: (togo/)) OR (Keywords: (tonga/)) OR (Keywords: ("trinidad and tobago" /)) OR (Keywords: (tunisia/)) OR (Keywords: (turkmenistan/)) OR (Keywords: (uganda/)) OR (Keywords: (ukraine/)) OR (Keywords: (united arab emirates/)) OR (Keywords: (uruguay/)) OR (Keywords: (uzbekistan/)) OR (Keywords: (vanuatu/)) OR (Keywords: (venezuela/)) OR (Keywords: (vietnam/)) OR (Keywords: (west indies/)) OR (Keywords: (yemen/)) OR (Keywords: (zambia/)) OR (Keywords: (zimbabwe/)))) AND Any Field: - (((Keywords: (australasia/)) OR (Keywords: (exp australia/)) OR (Keywords: (austria/)) OR (Keywords: (baltic states/)) OR (Keywords: (belgium/)) OR (Keywords: (exp canada/)) OR (Keywords: (chile/)) OR (Keywords: (colombia/)) OR (Keywords: (costa rica/)) OR

(Keywords: (czech republic/)) OR (Keywords: (exp denmark/)) OR (Keywords: (estonia/)) OR (Keywords: (europe/)) OR (Keywords: (finland/)) OR (Keywords: (exp france/)) OR (Keywords: (exp germany/)) OR (Keywords: (greece/)) OR (Keywords: (hungary/)) OR (Keywords: (iceland/)) OR (Keywords: (ireland/)) OR (Keywords: (israel/)) OR (Keywords: (exp italy/)) OR (Keywords: (exp japan/)) OR (Keywords: (korea/)) OR (Keywords: (latvia/)) OR (Keywords: (lithuania/)) OR (Keywords: (luxembourg/)) OR (Keywords: (mexico/)) OR (Keywords: (netherlands/)) OR (Keywords: (new zealand/)) OR (Keywords: (north america/)) OR (Keywords: (exp norway/)) OR (Keywords: (poland/)) OR (Keywords: (portugal/)) OR (Keywords: (exp "republic of korea" /)) OR (Keywords: ("scandinavian and nordic countries" /)) OR (Keywords: (slovakia/)) OR (Keywords: (slovenia/)) OR (Keywords: (spain/)) OR (Keywords: (sweden/)) OR (Keywords: (switzerland/)) OR (Keywords: (turkey/)) OR (Keywords: (exp united kingdom/)) OR (Keywords: (exp united states/)) OR (Keywords: (European Union/)) OR (Keywords: (Developed Countries/)))))

For peer review only

Appendix 3: CINAHL

#	Question	Results
S1	MW "harm# reduction#"	4,889
S2	MW "risk# reduction#"	0
S3	TI protective N2 strateg* OR AB protective N2 strateg* OR SU protective N2 strateg*	1,167
S4	SU ((reduc* or minimi*) N5 (harm# or harmful or risk#)) OR TI ((reduc* or minimi*) N5 (harm# or harmful or risk#)) OR AB ((reduc* or minimi*) N5 (harm# or harmful or risk#))	96,037
S5	S1 OR S2 OR S3 OR S4	97,005
S6	MW "Health Personnel#"	112,080
S7	MW "Social Worker#"	11,786
S8	MW Counselors	4,471
S9	MW "Health Occupation#"	5,719
S10	MW "Allied Health Occupation#"	0
S11	MW "Allied Health Personnel#"	4,781
S12	TI (worker or psychoeducator or psycho-educator or educator or nurse or criminologist or psychologist or clinician or practitioner or physician or professional or provider or counse#lor or counse#lors or caregiver or giver or therapist or psychotherapist or staff or personnel or employee or doctor) OR AB (worker or psychoeducator or psycho-educator or educator or nurse or criminologist or psychologist or clinician or practitioner or physician or professional or provider or counse#lor or counse#lors or caregiver or giver or therapist or psychotherapist or staff or personnel or employee or doctor) OR SU (worker or psychoeducator or psycho-educator or educator or nurse or criminologist or psychologist or clinician or practitioner or physician or professional or provider or counse#lor or counse#lors or caregiver or giver or therapist or psychotherapist or staff or personnel or employee or doctor)	1,762,879
S13	S6 OR S7 OR S8 OR S9 OR S10 OR S11 OR S12	1,765,867

For peer review only

S14	MW Cannabis	11,138
S15	MW Marijuana	2,219
S16	TI (mari#uana or cannabis or hashish or Pot or weed or tetrahydrocannabinol or THC or CDB or cannabidiol or cannabinoid#) OR AB (mari#uana or cannabis or hashish or Pot or weed or tetrahydrocannabinol or THC or CDB or cannabidiol or cannabinoid#) OR SU (mari#uana or cannabis or hashish or Pot or weed or tetrahydrocannabinol or THC or CDB or cannabidiol or cannabinoid#)	26,526
S17	S14 OR S15 OR S16	26,526
S18	S5 AND S13 AND S17	147
S19	S5 AND S13 AND S17	147
S20	S5 AND S13 AND S17	147
S21	S5 AND S13 AND S17	72
S22	SU afghanistan/ or africa/ or africa, northern/ or africa, central/ or africa, eastern/ or "africa south of the sahara"/ or africa, southern/ or africa, western/ or albania/ or algeria/ or andorra/ or angola/ or "antigua and barbuda"/ or argentina/ or armenia/ or azerbaijan/ or bahamas/ or bahrain/ or bangladesh/ or barbados/ or belize/ or benin/ or bhutan/ or bolivia/ or borneo/ or "bosnia and herzegovina"/ or botswana/ or brazil/ or brunei/ or bulgaria/ or burkina faso/ or burundi/ or cabo verde/ or cambodia/ or cameroon/ or central african republic/ or chad/ or exp china/ or comoros/ or congo/ or cote d'ivoire/ or croatia/ or cuba/ or "democratic republic of the congo"/ or cyprus/ or djibouti/ or dominica/ or dominican republic/ or ecuador/ or egypt/ or el salvador/ or equatorial guinea/ or eritrea/ or eswatini/ or ethiopia/ or fiji/ or gabon/ or gambia/ or "georgia (republic)"/ or ghana/ or grenada/ or guatemala/ or guinea/ or guinea-bissau/ or guyana/ or haiti/ or honduras/ or independent state of samoa/ or exp india/ or indian ocean islands/ or indochina/ or indonesia/ or iran/ or iraq/ or jamaica/ or jordan/ or kazakhstan/ or kenya/ or kosovo/ or kuwait/ or kyrgyzstan/ or laos/ or lebanon/ or liechtenstein/ or lesotho/ or liberia/ or libya/ or madagascar/ or malaysia/ or malawi/ or	159,916

For peer review only

	mali/ or malta/ or mauritania/ or mauritius/ or mekong valley/ or melanesia/ or micronesia/ or monaco/ or mongolia/ or montenegro/ or morocco/ or mozambique/ or myanmar/ or namibia/ or nepal/ or nicaragua/ or niger/ or nigeria/ or oman/ or pakistan/ or palau/ or exp panama/ or papua new guinea/ or paraguay/ or peru/ or philippines/ or qatar/ or "republic of belarus"/ or "republic of north macedonia"/ or romania/ or exp russia/ or rwanda/ or "saint kitts and nevis"/ or saint lucia/ or "saint vincent and the grenadines"/ or "sao tome and principe"/ or saudi arabia/ or serbia/ or sierra leone/ or senegal/ or seychelles/ or singapore/ or somalia/ or south africa/ or south sudan/ or sri lanka/ or sudan/ or suriname/ or syria/ or taiwan/ or tajikistan/ or tanzania/ or thailand/ or timor-leste/ or togo/ or tonga/ or "trinidad and tobago"/ or tunisia/ or turkmenistan/ or uganda/ or ukraine/ or united arab emirates/ or uruguay/ or uzbekistan/ or vanuatu/ or venezuela/ or vietnam/ or west indies/ or yemen/ or zambia/ or zimbabwe/	
S23	SU australasia/ or exp australia/ or austria/ or baltic states/ or belgium/ or exp canada/ or chile/ or colombia/ or costa rica/ or czech republic/ or exp denmark/ or estonia/ or europe/ or finland/ or exp france/ or exp germany/ or greece/ or hungary/ or iceland/ or ireland/ or israel/ or exp italy/ or exp japan/ or korea/ or latvia/ or lithuania/ or luxembourg/ or mexico/ or netherlands/ or new zealand/ or north america/ or exp norway/ or poland/ or portugal/ or exp "republic of korea"/ or "scandinavian and nordic countries"/ or slovakia/ or slovenia/ or spain/ or sweden/ or switzerland/ or turkey/ or exp united kingdom/ or exp united states/ OR European Union/ OR Developed Countries/	158,684
S24	S22 NOT S23	154,938
S25	S21 NOT S24	66

For peer review only

Appendix 4: Web of Science

#	Question	Results
#1	TS=(harm reduction OR risk reduction behavior OR (protective NEAR/2 strateg*) OR ((reduc* OR minimi*) NEAR/5 (harm OR harms OR harmful OR risk OR risks)))	329,523
#2	TS=(cannabis OR marijuana OR mari$uana OR hashish OR pot OR weed OR tetrahydrocannabinol OR THC OR CDB OR cannabidiol OR cannabinoid$)	305,377
#3	TS=(health personnel OR social worker$ OR counselor$ OR health occupation$ OR worker$ OR psychoeducator$ OR psycho-educator$ OR nurse$ OR criminologist$ OR psychologist$ OR clinician$ OR practitioner$ OR physician$ OR professional$ OR provider$ OR co$nselor OR co$nselors OR caregiver$ OR giver$ OR therapist$ OR psychotherapist$ OR staff$ OR personnel$ OR employee$ OR doctor$)	2,593,408
#4	#1 AND #2 AND #3	331
#5	#4 and English or French (Languages)	324
#6	#5 and [Countries/Regions filter: pays de l'OCDE]	300

For peer review only

Appendix 5: Embase

1	Harm Reduction/	8189
2	Risk Reduction Behavior/	115695
3	(protective adj2 strateg*).ab,kf,ti.	3987
4	((reduc* or minimi*) adj5 (harm? or harmful or risk?)).ab,kf,ti.	345174
**5**	1 or 2 or 3 or 4	414447
6	exp Health Personnel/	1843814
7	Social Workers/	12409
8	Counselors/	3692
9	exp Health Occupations/	24456
10	exp Allied Health Occupations/	392205
11	exp Allied Health Personnel/	567029
12	(worker? or (psychoeducator? or psycho-educator?) or educator? or nurse? or criminologist? or psychologist? or clinician? or practitioner? or physician? or professional? or provider? or co?nselor or co?nselors or caregiver? or giver? or therapist? or psychotherapist? or staff? or personnel? or employee? or doctor?).ab,kf,ti.	2640146
**13**	6 or 7 or 8 or 9 or 10 or 11 or 12	3761559
14	Cannabis/	40937
15	"Marijuana Use"/	12636
16	Marijuana Abuse/	6445
17	Marijuana Smoking/	4026
18	(mari?uana or cannabis or has?hish or Pot or weed or tetrahydrocannabinol or THC or CDB or cannabidiol or cannabinoid?).ab,kf,ti.	129932
**19**	14 or 15 or 16 or 17 or 18	142957
20	5 and 13 and 19	569
21	limit 32 to yr="1990 -Current"	569
22	limit 21 to (english or french)	560
23	Filtre OCDE	520

For peer review only

Appendix 6: Sociological Abstracts

(((ti(health personnel OR social worker OR social workers OR counselor OR counselors OR conselor OR conselors OR health occupation OR health occupations OR worker* OR psychoeducator* OR psycho-educator* OR educator* OR nurse* OR criminologist* OR psychologist* OR clinician* OR professional* OR provider* OR caregiver* OR giver* OR therapist* OR psychotherapist* OR staff* OR personnel* OR employee* OR doctor*) OR ab(health personnel OR social worker OR social workers OR counselor OR counselors OR conselor OR conselors OR health occupation OR health occupations OR worker* OR psychoeducator* OR psycho-educator* OR educator* OR nurse* OR criminologist* OR psychologist* OR clinician* OR professional* OR provider* OR caregiver* OR giver* OR therapist* OR psychotherapist* OR staff* OR personnel* OR employee* OR doctor*) OR su(health personnel OR social worker OR social workers OR counselor OR counselors OR conselor OR conselors OR health occupation OR health occupations OR worker* OR psychoeducator* OR psycho-educator* OR educator* OR nurse* OR criminologist* OR psychologist* OR clinician* OR professional* OR provider* OR caregiver* OR giver* OR therapist* OR psychotherapist* OR staff* OR personnel* OR employee* OR doctor*)) AND (ti(cannabis OR marijuana OR mari*uana OR hashish OR pot OR weed OR tetrahydrocannabinol OR THC OR CDB OR cannabidiol OR cannabinoid*) OR ab(cannabis OR marijuana OR mari*uana OR hashish OR pot OR weed OR tetrahydrocannabinol OR THC OR CDB OR cannabidiol OR cannabinoid*) OR su(cannabis OR marijuana OR mari*uana OR hashish OR pot OR weed OR tetrahydrocannabinol OR THC OR CDB OR cannabidiol OR cannabinoid*)) AND ((ti((reduc* OR minimi*) NEAR/5 (harm OR harms OR harmful OR risk OR risks)) OR ab((reduc* OR minimi*) NEAR/5 (harm OR harms OR harmful OR risk OR risks)) OR su((reduc* OR minimi*) NEAR/5 (harm OR harms OR harmful OR risk OR risks))) OR (ti(protective NEAR/2 strateg*) OR ab(protective NEAR/2 strateg*) OR su(protective NEAR/2 strateg*)))) AND la.exact("ENG")) AND yr(1990-2029)

For peer review only

Appendix 7: Google Scholar

cannabis intitle:nurse OR intitle:clinician OR intitle:practitioner OR intitle:physician OR intitle:caregiver OR intitle:giver OR intitle:doctor "harm reduction" = 105

cannabis intitle:nurses OR intitle:clinicians OR intitle:practitioners OR intitle:physicians OR intitle:caregivers OR intitle:givers OR intitle:doctors "harm reduction" = 242

cannabis intitle:psychoeducator OR intitle:educator OR intitle:therapist OR intitle:psychotherapist OR intitle:criminologist OR intitle:psychologist "harm reduction" = 19

cannabis intitle:psychoeducators OR intitle:educators OR intitle:therapists OR intitle:psychotherapists OR intitle:criminologists OR intitle:psychologists "harm reduction" = 53

cannabis intitle:worker OR intitle:staff OR intitle:personnel OR intitle:employee OR intitle:professional OR intitle:provider OR intitle:counselor OR intitle:conselor "harm reduction" = 136

cannabis intitle: workers OR intitle:staffs OR intitle:personnels OR intitle:employees OR intitle:professionals OR intitle:providers OR intitle:counselors OR intitle:conselors "harm reduction" = 5

cannabis intitle:infirmier OR intitle:médecin OR intitle:clinicien OR intitle:psychoéducateur OR intitle:éducateur OR intitle:thérapeute OR intitle:psychothérapeute OR intitle:criminologue OR intitle:psychologue “reduction des méfaits” = 1

cannabis intitle:travailleur OR intitle:personnel OR intitle:employé OR intitle:professionnel OR intitle:conseiller OR intitle:intervenant “reduction des méfaits” = 5

cannabis intitle:infirmiers OR intitle:médecins OR intitle:cliniciens OR intitle:psychoéducateurs OR intitle:éducateurs OR intitle:thérapeutes OR intitle:psychothérapeutes OR intitle:criminologues OR intitle:psychologues “reduction des méfaits” = 3

cannabis intitle:travailleurs OR intitle:personnels OR intitle:employés OR intitle:professionnels OR intitle:conseillers OR intitle:intervenants “reduction des méfaits” = 12

cannabis intitle:infirmière OR intitle:cliniciennne OR intitle:psychoéducatrice OR intitle:éducatrice OR intitle:criminologue OR intitle:psychologue “reduction des méfaits” = 2

cannabis intitle:travailleuse OR intitle:employée OR intitle:professionnelle OR intitle:conseillère OR intitle:intervenante “reduction des méfaits” = 1

cannabis intitle:infirmières OR intitle:cliniciennes OR intitle:psychoéducatrices OR intitle:éducatrices OR intitle:criminologues OR intitle:psychologues “reduction des méfaits” = 2

cannabis intitle:travailleuses OR intitle:employées OR intitle:professionnelles OR intitle:conseillères OR intitle:intervenantes “reduction des méfaits” = 2

marijuana intitle:nurse OR intitle:clinician OR intitle:practitioner OR intitle:physician OR intitle:caregiver OR intitle:giver OR intitle:doctor "harm reduction" = 139

marijuana intitle:nurses OR intitle:clinicians OR intitle:practitioners OR intitle:physicians OR intitle:caregivers OR intitle:givers OR intitle:doctors "harm reduction" = 238

marijuana intitle:psychoeducator OR intitle:educator OR intitle:therapist OR intitle:psychotherapist OR intitle:criminologist OR intitle:psychologist "harm reduction" = 19

For peer review only

marijuana intitle:psychoeducators OR intitle:educators OR intitle:therapists OR intitle:psychotherapists OR intitle:criminologists OR intitle:psychologists "harm reduction" = 48

marijuana intitle:worker OR intitle:staff OR intitle:personnel OR intitle:employee OR intitle:professional OR intitle:provider OR intitle:counselor OR intitle:conselor "harm reduction" = 140

marijuana intitle:workers OR intitle:staffs OR intitle:personnels OR intitle:employees OR intitle:professionals OR intitle:providers OR intitle:counselors OR intitle:conselors "harm reduction" = 573

marijuana intitle:infirmier OR intitle:médecin OR intitle:clinicien OR intitle:psychoéducateur OR intitle:éducateur OR intitle:thérapeute OR intitle:psychothérapeute OR intitle:criminologue OR intitle:psychologue “reduction des méfaits” = 1

marijuana intitle:travailleur OR intitle:personnel OR intitle:employé OR intitle:professionnel OR intitle:conseiller OR intitle:intervenant “reduction des méfaits” = 5

marijuana intitle:infirmiers OR intitle:médecins OR intitle:cliniciens OR intitle:psychoéducateurs OR intitle:éducateurs OR intitle:thérapeutes OR intitle:psychothérapeutes OR intitle:criminologues OR intitle:psychologues “reduction des méfaits” = 3

marijuana intitle:travailleurs OR intitle:personnels OR intitle:employés OR intitle:professionnels OR intitle:conseillers OR intitle:intervenants “reduction des méfaits” = 12

marijuana intitle:infirmière OR intitle:clinicienne OR intitle:psychoéducatrice OR intitle:éducatrice OR intitle:criminologue OR intitle:psychologue “reduction des méfaits” = 2

marijuana intitle:travailleuse OR intitle:employée OR intitle:professionnelle OR intitle:conseillère OR intitle:intervenante “reduction des méfaits” = 1

marijuana intitle:infirmières OR intitle:cliniciennes OR intitle:psychoéducatrices OR intitle:éducatrices OR intitle:criminologues OR intitle:psychologues “reduction des méfaits” = 2

marijuana intitle:travailleuses OR intitle:employées OR intitle:professionnelles OR intitle:conseillères OR intitle:intervenantes “reduction des méfaits” = 2

For peer review only

Appendix 8: Google Web

cannabis intitle:nurse OR intitle:clinician OR intitle:practitioner OR intitle:physician OR intitle:caregiver OR intitle:giver OR intitle:doctor "harm reduction" filetype:pdf =

cannabis intitle:nurses OR intitle:clinicians OR intitle:practitioners OR intitle:physicians OR intitle:caregivers OR intitle:givers OR intitle:doctors "harm reduction" filetype:pdf =

cannabis intitle:psychoeducator OR intitle:educator OR intitle:therapist OR intitle:psychotherapist OR intitle:criminologist OR intitle:psychologist "harm reduction" filetype:pdf =

cannabis intitle:psychoeducators OR intitle:educators OR intitle:therapists OR intitle:psychotherapists OR intitle:criminologists OR intitle:psychologists "harm reduction" filetype:pdf =

cannabis intitle:worker OR intitle:staff OR intitle:personnel OR intitle:employee OR intitle:professional OR intitle:provider OR intitle:counselor OR intitle:conselor "harm reduction" filetype:pdf =

cannabis intitle: workers OR intitle:staffs OR intitle:personnels OR intitle:employees OR intitle:professionals OR intitle:providers OR intitle:counselors OR intitle:conselors "harm reduction" filetype:pdf =

cannabis intitle:infirmier OR intitle:médecin OR intitle:clinicien OR intitle:psychoéducateur OR intitle:éducateur OR intitle:thérapeute OR intitle:psychothérapeute OR intitle:criminologue OR intitle:psychologue “reduction des méfaits” filetype:pdf =

cannabis intitle:travailleur OR intitle:personnel OR intitle:employé OR intitle:professionnel OR intitle:conseiller OR intitle:intervenant “reduction des méfaits” filetype:pdf =

cannabis intitle:infirmiers OR intitle:médecins OR intitle:cliniciens OR intitle:psychoéducateurs OR intitle:éducateurs OR intitle:thérapeutes OR intitle:psychothérapeutes OR intitle:criminologues OR intitle:psychologues “reduction des méfaits” filetype:pdf =

cannabis intitle:travailleurs OR intitle:personnels OR intitle:employés OR intitle:professionnels OR intitle:conseillers OR intitle:intervenants “reduction des méfaits” filetype:pdf =

cannabis intitle:infirmière OR intitle:clinicìennne OR intitle:psychoéducatrice OR intitle:éducatrice OR intitle:criminologue OR intitle:psychologue “reduction des méfaits” filetype:pdf =

cannabis intitle:travailleuse OR intitle:employée OR intitle:professionnelle OR intitle:conseillère OR intitle:intervenante “reduction des méfaits” filetype:pdf =

cannabis intitle:infirmières OR intitle:cliniciennes OR intitle:psychoéducatrices OR intitle:éducatrices OR intitle:criminologues OR intitle:psychologues “reduction des méfaits” filetype:pdf =

cannabis intitle:travailleuses OR intitle:employées OR intitle:professionnelles OR intitle:conseillères OR intitle:intervenantes “reduction des méfaits” filetype:pdf =

marijuana intitle:nurse OR intitle:clinician OR intitle:practitioner OR intitle:physician OR intitle:caregiver OR intitle:giver OR intitle:doctor "harm reduction" filetype:pdf =

marijuana intitle:nurses OR intitle:clinicians OR intitle:practitioners OR intitle:physicians OR intitle:caregivers OR intitle:givers OR intitle:doctors "harm reduction" filetype:pdf =

For peer review only

marijuana intitle:psychoeducator OR intitle:educator OR intitle:therapist OR intitle:psychotherapist OR intitle:criminologist OR intitle:psychologist "harm reduction" filetype:pdf =

marijuana intitle:psychoeducators OR intitle:educators OR intitle:therapists OR intitle:psychotherapists OR intitle:criminologists OR intitle:psychologists "harm reduction" filetype:pdf =

marijuana intitle:worker OR intitle:staff OR intitle:personnel OR intitle:employee OR intitle:professional OR intitle:provider OR intitle:counselor OR intitle:conselor "harm reduction" filetype:pdf =

marijuana intitle:workers OR intitle:staffs OR intitle:personnels OR intitle:employees OR intitle:professionals OR intitle:providers OR intitle:counselors OR intitle:conselors "harm reduction" filetype:pdf =

marijuana intitle:infirmier OR intitle:médecin OR intitle:clinicien OR intitle:psychoéducateur OR intitle:éducateur OR intitle:thérapeute OR intitle:psychothérapeute OR intitle:criminologue OR intitle:psychologue “reduction des méfaits” filetype:pdf =

marijuana intitle:travailleur OR intitle:personnel OR intitle:employé OR intitle:professionnel OR intitle:conseiller OR intitle:intervenant “reduction des méfaits” filetype:pdf =

marijuana intitle:infirmiers OR intitle:médecins OR intitle:cliniciens OR intitle:psychoéducateurs OR intitle:éducateurs OR intitle:thérapeutes OR intitle:psychothérapeutes OR intitle:criminologues OR intitle:psychologues “reduction des méfaits” filetype:pdf =

marijuana intitle:travailleurs OR intitle:personnels OR intitle:employés OR intitle:professionnels OR intitle:conseillers OR intitle:intervenants “reduction des méfaits” filetype:pdf =

marijuana intitle:infirmière OR intitle:clinicienne OR intitle:psychoéducatrice OR intitle:éducatrice OR intitle:criminologue OR intitle:psychologue “reduction des méfaits” filetype:pdf =

marijuana intitle:travailleuse OR intitle:employée OR intitle:professionnelle OR intitle:conseillère OR intitle:intervenante “reduction des méfaits” filetype:pdf =

marijuana intitle:infirmières OR intitle:cliniciennes OR intitle:psychoéducatrices OR intitle:éducatrices OR intitle:criminologues OR intitle:psychologues “reduction des méfaits” filetype:pdf =

marijuana intitle:travailleuses OR intitle:employées OR intitle:professionnelles OR intitle:conseillères OR intitle:intervenantes “reduction des méfaits” filetype:pdf =

For peer review only

Appendix 9: BASE

tit:(nurse* clinician* practitioner* physician* caregiver* giver* doctor* psychoeducator* educator* therapist* psychotherapist* criminologist* psychologist* worker* staff* personnel* employee* professional* provider* counselor*) subj:(cannabis marijuana) subj:"harm reduction" = 4

tit:(infirmier* OU médecin* OU clinicien* OU psychoéducat* OU éducat* OU criminologue* OU psychologue* OU travailleu* OU employé* OU professionnel* OU conseill* OU intervenant*) subj:cannabis subj:marijuana subj:"réduction des méfaits" = 0

For peer review only

Appendix 10: Érudit

(Titre, résumé, mots-clés : harm reduction OU harm minimisation OU risk reduction OU risks reduction OU risk minimisation OU réduction des méfaits OU réduction des risques) ET (Titre, résumé, mots-clés : cannabis OU marijuana) ET (Titre, résumé, mots-clés : nurse* OU clinician* OU practitioner* OU physician* OU caregiver* OU giver* OU doctor* OU psychoeducator* OU educator* OU therapist* OU psychotherapist* OU criminologist* OU psychologist* OU worker* OU staff* OU personnel* OU employee* OU professional* OU provider* OU counselor* OU infirmier* OU médecin* OU clinicien* OU psychoéducat* OU éducat* OU criminologue* OU psychologue* OU travailleu* OU employé* OU professionnel* OU conseill* OU intervenant*) = 27

For peer review only
